# Unraveling the energetic significance of chemical events in enzyme catalysis via machine-learning based regression approach

**DOI:** 10.1038/s42004-020-00379-w

**Published:** 2020-10-08

**Authors:** Zilin Song, Hongyu Zhou, Hao Tian, Xinlei Wang, Peng Tao

**Affiliations:** 1 Department of Chemistry, Center for Research Computing, Center for Drug Discovery, Design, and Delivery (CD4), Southern Methodist University, Dallas, TX 75275 USA; 2 Department of Statistical Science, Southern Methodist University, Dallas, TX 75275 USA

**Keywords:** Enzyme mechanisms, Method development, Computational chemistry

## Abstract

The bacterial enzyme class of β-lactamases are involved in benzylpenicillin acylation reactions, which are currently being revisited using hybrid quantum mechanical molecular mechanical (QM/MM) chain-of-states pathway optimizations. Minimum energy pathways are sampled by reoptimizing pathway geometry under different representative protein environments obtained through constrained molecular dynamics simulations. Predictive potential energy surface models in the reaction space are trained with machine-learning regression techniques. Herein, using TEM-1/benzylpenicillin acylation reaction as the model system, we introduce two model-independent criteria for delineating the energetic contributions and correlations in the predicted reaction space. Both methods are demonstrated to effectively quantify the energetic contribution of each chemical process and identify the rate limiting step of enzymatic reaction with high degrees of freedom. The consistency of the current workflow is tested under seven levels of quantum chemistry theory and three non-linear machine-learning regression models. The proposed approaches are validated to provide qualitative compliance with experimental mutagenesis studies.

## Abstract (web summary)

In light of bacterial resistance to β-lactam antibiotic drugs, understanding the hydrolysis reaction of β-lactamases is crucial. Here the authors use machine learning based regression algorithms to analyze the catalytic energy landscape of TEM-1.

## Introduction

Bacteria resistance to β-lactam antibiotic drugs poses severe threat to the global health^1^. One of the major causes of antibiotic resistance is the bacteria-produced enzymes, β-lactamases, which could effectively hydrolyze many types of β-lactam antibiotics^2,3^. Numerous studies have provided great insights into the mechanism behind the hydrolysis reaction of β-lactamases against β-lactam substrates. β-Lactamases are generally classified into four groups (Classes A, B, C, and D) based on their sequence similarity^4^. Class A, C, and D are serine-based β-lactamases (SβLs), and class B is zinc-based β-lactamases.

**Fig. 1:**
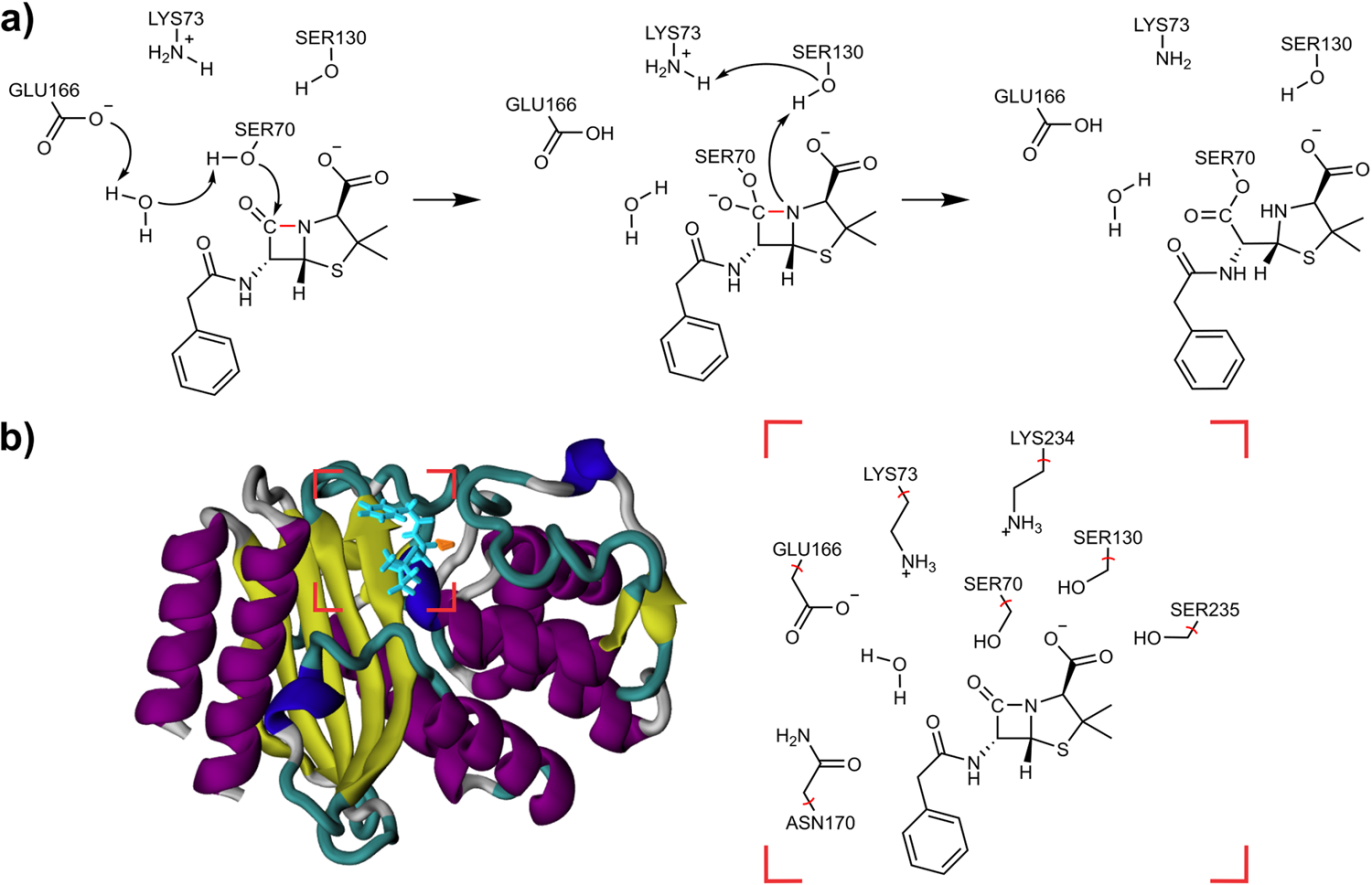
Acylation mechanism of Class A β-lactamases and the structure of TEM-1/benzylpenicillin Michaelis complex. **a** Acylation mechanism of TEM-1 and benzylpenicillin with Glu166 acting as a general base. The β-lactam scissile bond is noted in red; **b** Crystal structure of TEM-1 complexed with benzylpenicillin and the selection of QM atoms.

Class A β-lactamases is the dominant group and poses serious threat against a wide range of substrates^5^. TEM-1 is a representative class A SβL and the most common β-lactamase among Gram-negative bacteria strains. Numerous experimental studies have been carried out to delineate the functions of the residues at the catalytic binding site^6–15^. Based on these studies, one widely accepted mechanism was proposed that Glu166 acts as a general base during the acylation process of benzylpenicillin hydrolysis (Fig. 1a)^7^. The hydroxyl group of Ser70 first attacks the β-lactam carbonyl carbon to form a tetrahedral intermediate, with its proton delivered to the bridging catalytic water. The bridging water in turn donates a proton to the deprotonated carboxyl group of Glu166. Upon the formation of the tetrahedral intermediate, the fully protonated Lys73 activates the nearby Ser130 to protonate the β-lactam nitrogen, which cleaves the β-lactam scissile bond and completes the acylation half of β-lactam hydrolysis. Other residues including Asn170 and Ser235 were also validated to contribute hydrogen bonding interactions that are critical for the formation of the Michaelis complex between TEM-1 and the benzylpenicillin substrate^8,9^.

Computational methods have been employed to further illustrate the detailed TEM-1 catalytic mechanism^16–23^. Hybrid quantum mechanics/molecular mechanics (QM/MM) and molecular dynamics (MD) studies have validated the acylation mechanism and provided reaction pathways on the potential energy surface (PES)^16–18^. However, limitations persist as well as other computational efforts focusing on biochemical catalytic reactions in geometrical spaces with high degrees of freedom. As a single pathway within a fixed external MM potential may not well represent the overall enzymatic reaction mechanism, a comprehensive description using multiple potential pathways under various MM potential fields is generally preferred. In addition, free energy simulations have provided accurate energetic barrier profiles for similar β-lactamases systems^24^.

Chain-of-states (CoS) pathways optimization methods could provide minimum energy pathways (MEPs) at a reasonable computational cost. Using this method, reoptimizing the reaction pathway under modified external MM potential fields is feasible. However, further analysis on this collection of reaction pathways is hindered by the massive correlations between the geometrical degrees of freedom along the reaction progress. Machine-learning-based techniques have been shown to be the plausible methods to model the systems with high dimensionality. It has also been successfully employed in various subjects, including protein allosteric analysis^25–28^, drug discovery^29,30^, and accelerating QM/MM calculations^31–34^. In this regard, machine-learning-based regression algorithms could be utilized to predict reaction pathway energetic profiles with sufficient training data. As a predictive PES model could be trained on structural descriptors as input features, the resulted model should reflect the underlying correlations among those features. Therefore, the model could be used to quantify the functional importance of chemical properties associated with the structural descriptors. Many generalized methods for quantifying feature importance or variable contribution were proposed for linear models, but few are available for non-linear models^35^.

To develop quantitative models that correlate enzyme catalysis activity with each chemical event, we applied machine-learning-based non-linear regression methods to analyze multiple minimum energy pathways representing the enzyme catalytic landscape. The minimum energy pathways are generated using CoS approach under various MM external potentials sampled from constrained MD simulations.

## Results

### Benzylpenicillin acylation pathways

**Fig. 2:**
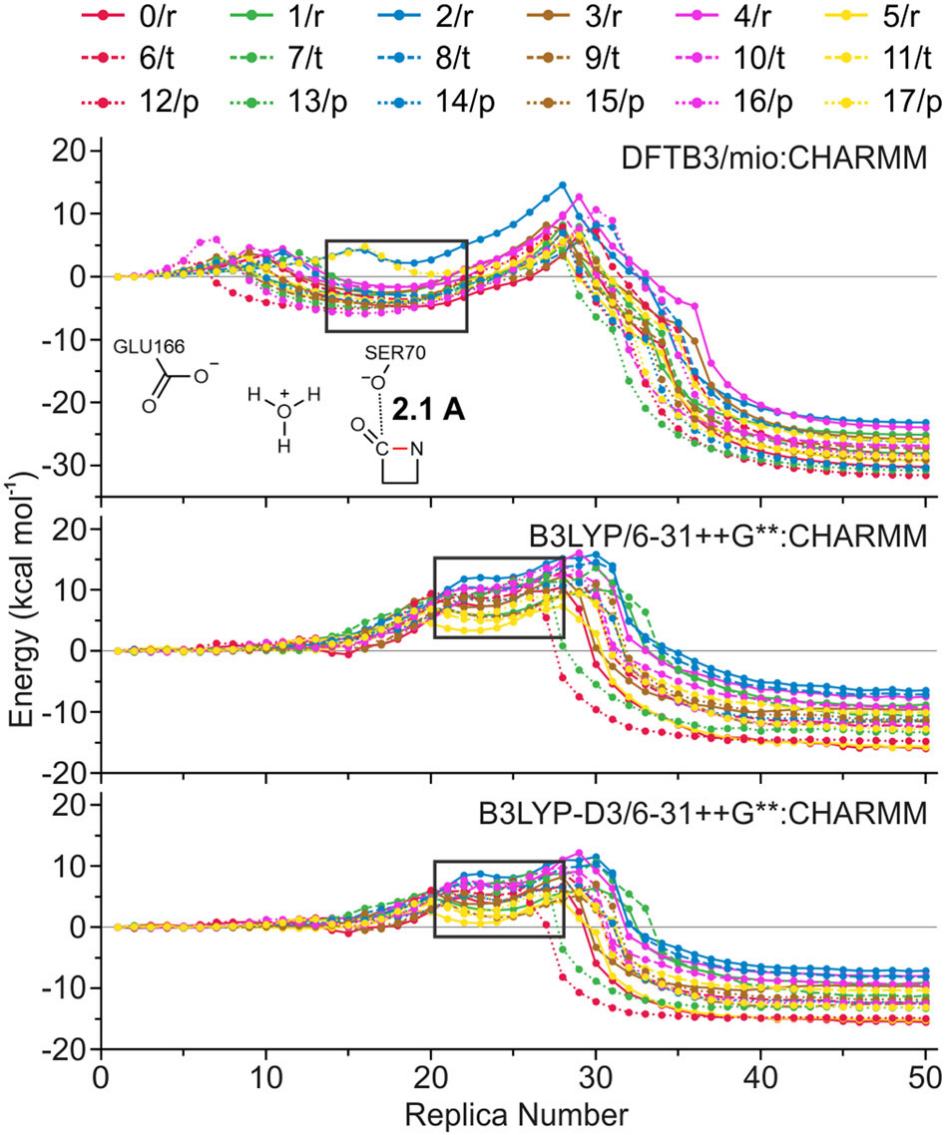
QM/MM chain-of-states pathway profiles. Reaction pathways calculated from DFTB3/mio:CHARMM, B3LYP/6–31++G**:CHARMM, and B3LYP-D3/6–31++G**:CHARMM levels of theory. The black rectangles highlight the tetrahedral intermediates region along the energy profiles.

The roles of active site residues of TEM-1 have been thoroughly studied by previous experimental and computational studies^8–11,16–18^. Accordingly, we selected a QM region of 92 atoms, including key catalytic residues as shown in Fig. 1b. In order to obtain MEPs in various external MM fields, three configurations from an initial pathway were selected and used as the start points for independent MD simulations (see “Methods” section, Supplementary Figs. 1 and 2). During each MD run, the QM atoms were fixed in their position while the MM atoms were allowed to move freely. Eighteen representative conformations were selected from the MD trajectories, on which the QM/MM geometry optimizations were later performed. Based on those representative configurations, 18 reaction pathways were optimized using the RPATh with constraints^36–39^ CoS method implemented in CHARMM^40^. Two QM levels of theory were adopted for geometry and pathway optimizations in this study: The Third Order Density Functional Tight Binding theory (DFTB3) with the mio parameter set (DFTB3/mio:CHARMM)^41,42^, and the density functional theory (DFT) B3LYP hybrid functional with the 6–31G basis set (B3LYP/6–31G:CHARMM)^43,44^. The single point energies on the B3LYP optimized pathways were further refined with three larger basis sets: 6–31+G*, 6–31++G**, and 6–311++G**. Whereas the dispersion effect has been validated to play a vital role in enzymatic reactions^45^, the empirical dispersion corrected B3LYP functional^46^, B3LYP-D3, is also introduced for single point energy refinement: B3LYP-D3/6–31++G**:CHARMM, and B3LYP-D3/6–311++G**:CHARMM. A total number of seven different QM levels of theory were tested in the current study. All calculated pathway energetic profiles are presented in Fig. 2 and Supplementary Figs. 3–6.

Two energy barriers separated by an intermediate state are identified in all calculated pathways. In the acylation mechanism that Glu166 acts as a general base, a previous debate focuses on whether the tetrahedral intermediate is more stable than the Michaelis reactant^16–18^. According to the B3LYP/6–31+G*:CHARMM calculations by Hermann et al.^17^, this intermediate is lower in energy by 15.5 kcal mol^−1^ comparing to the reactant. In Meroueh and coworker’s^18^ computational study, it was shown that the tetrahedral is higher by 12.0 kcal mol^−1^ than the Michaelis complex.

As highlighted in the black rectangles in Fig. 2, the carbonyl tetrahedral intermediate state could be obtained from all B3LYP reaction pathway optimizations. However, 16 out of 18 DFTB3 optimized pathways demonstrated that the intermediates are lower in energy than the reactant, whereas all B3LYP pathways show that the energies of tetrahedral states are well elevated from the reactant. In addition, the tetrahedral intermediates from our DFTB3 calculations are structurally different from Hermann et al.^17^. As shown in Fig. 2, the average distance between Ser70 Oγ and the carbonyl carbon is 2.1 Å, comparing to 1.45 Å reported by Hermann et al. Also, it is noted that tetrahedral intermediates from our DFTB3 calculations are accompanied by a hydronium formed by the catalytic water and negatively charged Glu166, whereas Hermann et al. observed a neutral catalytic water and protonated Glu166. Such disagreement could originate from the fundamental difference between the QM methodologies. Although the DFTB3/mio:CHARMM optimized pathways provide acylation barriers that are in good agreements with experiments, the configurational changes along the chain-of-replicas may not be reliable. The selection of QM region or the initial configuration used in the QM/MM calculations could also lead to a different conclusion. On the other hand, our B3LYP optimized reaction pathways agree with the results reported by Meroueh et al.^18^, showing that the potential energies of the tetrahedral intermediate are elevated from the reactant. Detailed barrier results of the acylation are compared with previous computational and experimental studies in Table 1. Moreover, albeit our B3LYP/6–311++G** single point energies give the most realistic average reaction barriers comparing to the experimental results, they are also shown to have the largest deviations among individual profiles (Supplementary Fig. 5). We also note that, the dispersion corrected B3LYP calculations generally led to 3–5 kcal mol^−1^ decrease of the activation barriers during the acylation, which is consistent with previous observations^47^.

**Table 1:** Comparison of acylation energy barriers of the current and previous works.

Source^a^	Energy barriers (kcal mol^−1^)^b^			Method^c^
	MC-TI	TI-AE	Overall	
This study	3.6(3)	11.4(1)	11.4(1)	DFTB3/mio:CHARMM, CoS
This study	4.4(5)	3.3(1)	7.1(5)	B3LYP/6–31G:CHARMM, CoS
This study	7.9(7)	3.8(2)	10.9(4)	B3LYP/6–31+G*:CHARMM, CoS
**This study**	**8.6(9)**	**3.8(7)**	**11.9(4)**	**B3LYP/6–31**++G**:CHARMM, CoS
This study	5.7(8)	3.4(3)	8.0(3)	B3LYP-D3/6–31++G**:CHARMM, CoS
This study	9.1(3)	3.9(7)	12.7(3)	B3LYP/6–311++G**:CHARMM, CoS
This study	6.2(4)	3.6(8)	9.0(1)	B3LYP-D3/6–311++G**:CHARMM, CoS
Pitarch et al.^16^^,d^	18.2(9)	12.9(1)	18.2(9)	AM1:CHARMM, IRC
Hermann et al.^17^	19.6	16.4	19.6	AM1:CHARMM, PESs
Hermann et al.^17^	8.7	7.1	8.7	B3LYP/6–31+G*:CHARMM, PESs
Meroueh et al.^18^^,d^	22.0	N/D^e^	22.0	MP2/6–31+G*:AMBER, PESs
Gibson et al.^12^	N/A	N/A	12.6(7)	293.15 K, Exp
Sirot et al.^13^	N/A	N/A	13.0(5)	310.15 K, Exp
Cheong et al.^14^	N/A	N/A	12.7(0)	293.15 K, Exp

^a^Computational acylation reaction profiles are constructed for the mechanism with Glu166 as a general base. The computational result with the best experimental compliance is marked in bold.

^b^MC-TI: Michaelis complex to tetrahedral intermediate; TI-AE: tetrahedral intermediate collapsing to acyl-enzyme product;

^c^*CoS* chain-of-states calculation, the reported barrier is the average value over 18 pathways; *IRC* intrinsic reaction coordinate calculation, *PESs* potential energy surface scan, *Exp* derived from experimental *k*_cat_ under the specified temperature;

^d^This study uses penicillanic acid instead of benzylpenicillin, the experimental acylation barrier of penicillanic acid is estimated to be 16–17 kcal mol^−1^;

^e^Barrier was reported to be “inconsequentially small”.

Although previous studies^17,18^ concluded that the formation of the tetrahedral intermediate is the rate limiting step during the acylation, our results do not necessarily comply with such conclusion. The B3LYP/6–31++G**:CHARMM pathways show that the Ser70 added tetrahedral intermediates are meta-stable states (black rectangles in Fig. 2). The total energies of these intermediates are 0.6(1) kcal mol^−1^ lower on average than the transition states of tetrahedral formation. Furthermore, as shown in Table 1, the optimized B3LYP/6–31++G** energy profiles present an average tetrahedral collapsing barrier of 3.8(7) kcal mol^−1^. Intuitively, such evidence suggests that the acylation is most likely a concerted one-step reaction.

### Regression model training

**Fig. 3:**
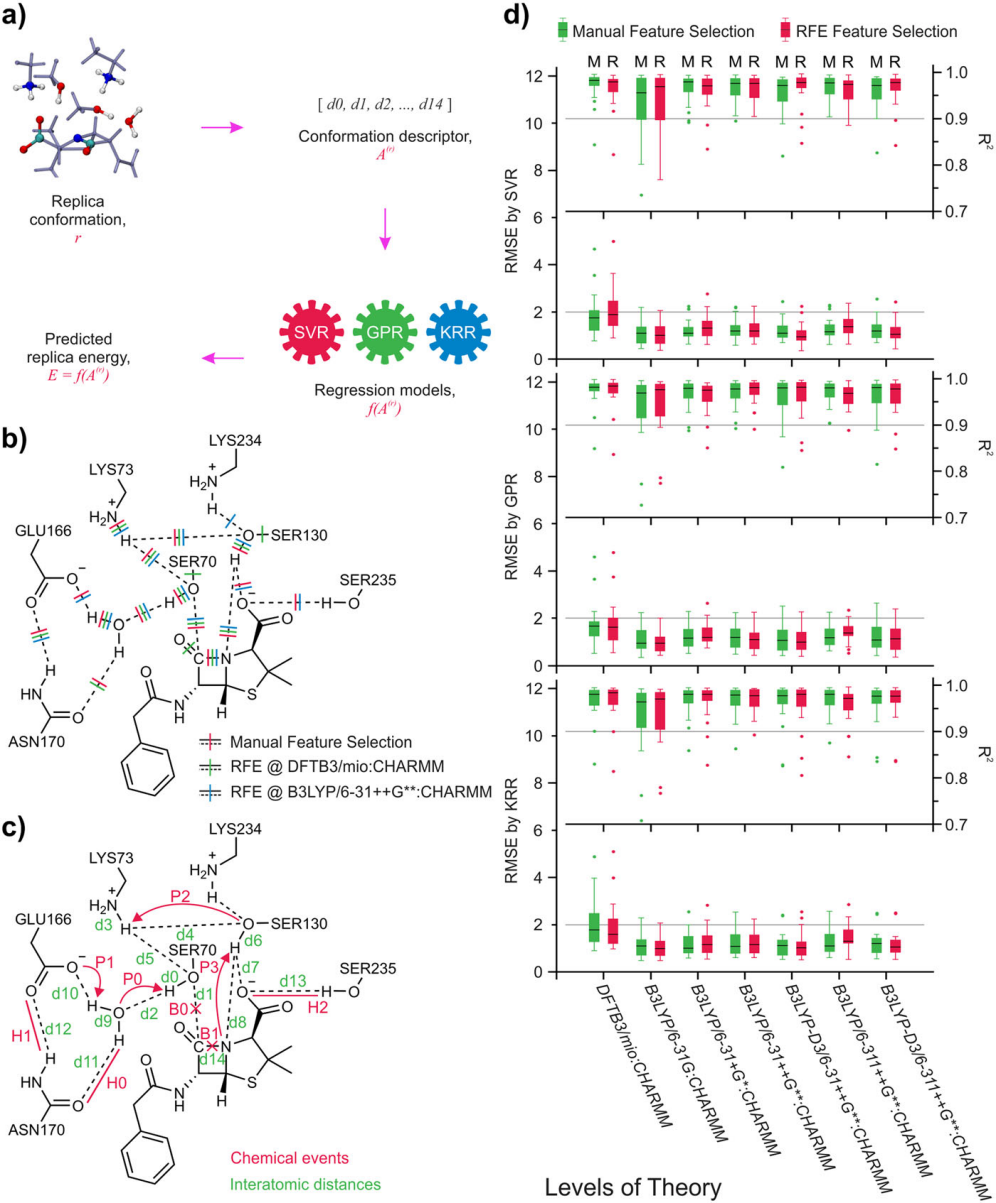
Description of the predictive PES models, the results of feature selection, and the benchmark of the regression models. **a** Schematic representation of the input/output data of the predictive PES models; **b** Features selected from RFE process with linear-kernel SVR and from manual selection, see also Supplementary Figs. 7 and 8; **c** Representations of interatomic distances used as the input feature vectors and the notation of chemical events; **d** Precision benchmark of regression models trained on datasets from various QM levels of theory. The “M” and “R” labels note the manual and RFE feature selected feature sets, respectively. Each box contains *n* = 18 prediction cases, the interquartile range (IQR) noted by the boxes are divided by the median (black lines), and the whiskers marks the first datum that are larger than 1.5 * IQR.

In order to decompose the energy contributions to each chemical event and determine the actual rate limiting step, predictive PES models were trained to bridge the conformational descriptors of each replica to its corresponding energy, as shown in Fig. 3a. Notably, since the reaction energy profiles are the relative energies with regard to the reactant, the interatomic distances used as input features are also the relative values from the corresponding reactant state. An appropriate selection of features is critical for the performance of machine-learning predictions. In our case, a total of 105 pairwise distances between bonded atoms—either through chemical bonding or hydrogen bonding—in the QM region are considered as initial features. Additionally, we note that some pioneer studies^33,34^ combining QM/MM and machine-learning techniques included the configurations of the MM atoms in the feature vectors as well. In our approach, the endpoints (i.e., the reactant and the acyl-enzyme product) as well as the chain-of-replicas were optimized in the selected external MM environment. The configurational difference between the optimized replica chains essentially reflects the contributions from the configuration of MM environments. Therefore, configurations of the MM atoms were omitted from the feature vector in the current study.

As the size of the dataset (900 replicas) is relatively small compared to the dimension (105 features), regression models are expected to fit poorly and unstably. In order to reduce the dimension of feature vectors, a recursive feature elimination (RFE) analysis using support vector regression (SVR) model with linear-kernel function was first performed on both DFTB3/mio:CHARMM and B3LYP/6–31++G**:CHARMM pathways. A total number of 15 most critical features were retained according to the acylation energy profiles from both levels of QM theory, as illustrated in Fig. 3b. Obviously, the RFE process could distinguish the key interatomic distances closely related with the reaction progress. Based on the RFE selected features and our prior knowledge with TEM-1/Benzylpenicillin hydrolysis, 15 interatomic distances were selected and used to construct the feature vector (Fig. 3c).

The performance of the regression models on predicting reaction energetic profiles were evaluated on the RFE and manual feature selections. Three machine-learning-based non-linear regression models were applied: support vector regression (SVR)^48^, Gaussian process regression (GPR)^49^, and kernel ridge regression (KRR)^50^. In the regression models, the DFTB3 optimized replica geometries were used as input to predict the DFTB3 replica energies. The B3LYP and B3LYP-D3 single point energies were predicted with the replica geometries from the B3LYP/6–31G:CHARMM pathway calculations. A total number of 18 rounds of cross-prediction are carried out recursively using 17 pathways as the training set and the remaining as the testing set. The predictive accuracy is assessed by root-mean-square error (RMSE) between the calculated and predicted energy profiles of the testing pathway (Fig. 3d), which is defined as:

1 $${\mathrm{RMSE}} = \left( {\mathop {\sum }\limits_{{\mathrm{r}} = 1}^{\mathrm{R}} \frac{{\left( {E_{{\mathrm{QM/MM}}}^{{\mathrm{(r)}}} - f\left( {{\mathbf{A}}^{\left( r \right)}} \right)} \right)^2}}{R}} \right)^{\!\!\frac{1}{2}},$$

where *R* is the total number of replicas on each pathway; $E_{{\mathrm{QM/MM}}}^{{\mathrm{(r)}}}$ is the QM/MM single point energy of the r-th replica; *f* is the trained regression model; **A**^(*r*)^ is the input feature vector at the r-th replica.

**Fig. 4:**
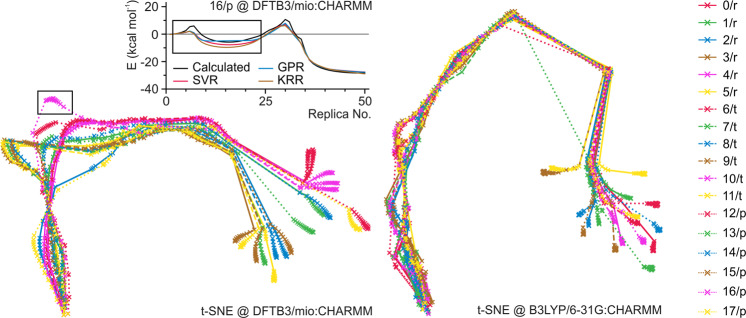
2D t-SNE dimensionality reduction of pathway geometries. 2D t-SNE dimensionality-reduced results of feature vectors from DFTB3/mio:CHARMM and B3LYP/6–31G:CHARMM optimized pathway geometries.

The overall prediction quality of regression models on B3LYP pathways are promising with the RMSE values lower than 2.0 kcal mol^–1^. The fitting quality on DFTB3/mio:CHARMM pathways is worse than that on B3LYP pathways regardless of the model used. The difference between the performance on B3LYP and DFTB3 pathways was explored through the analysis of the training input distribution by two-dimensional (2D) t-distributed stochastic neighbor embedding (2D t-SNE) method (Fig. 4). The reduced replica configurations of the 18 pathways from B3LYP pathways are more uniformly distributed along the reaction progress than the DFTB3 calculations. This suggests that the configurational changes along the DFTB3 pathways are more flexible and diverse compared to the B3LYP pathways. As a consequence, the variable space of the DFTB3 training sets is larger. Therefore, extra sampling is needed to achieve compatible fitting performance as the B3LYP training sets. The worst prediction comes from the pathway 16/p at DFTB3/mio:CHARMM level of theory. On this pathway, 2D t-SNE analysis shows that the training-validation set does not carry information in the region marked by the black rectangle (Fig. 4). The regression models are therefore under-fitted in this prediction space.

### Intrinsic energy contribution

Before assessing the energy contribution in the predictive models, the features are first grouped into feature subsets to reflect the joint contribution from critical chemical events (Fig. 3c). A detailed explanation of the chemical events is provided in Supplementary Table 1. One universal criterion to measure variable contribution is the decrease in prediction performance when a certain feature is dropped out from the model. Practically, we measure the joint contribution of feature subset by the difference between the fitting performance of a predictive model trained from full input feature set and the same model trained with the target feature subset set to zero. In this regard, the intrinsic energy contribution is defined as the RMSE between the predicted energetic pathway profiles of the two models:

2 $$I_{{\mathrm{a,intrinsic}}} = \left( {\mathop {\sum }\limits_{{\mathrm{r}} = 1}^{\mathrm{R}} \frac{{({f( {{\mathbf{A}}^{( r )}}) - f_{{\mathrm{a}} = 0}({{\mathbf{A}}^{(r)}})})^2}}{R}} \right)^{\frac{1}{2}},$$

where *R* is the total number of replicas on each pathway; *f* is the trained regression model; *f*_*a* = 0_ is the same model trained from input data with the target feature subset set to zero; **A**^(r)^ is the input feature vector at the r-th replica. For numerical comparisons between different regression models, the measurement used is the percentage of each intrinsic contribution over the sum of all feature subgroups (see also Supplementary Fig. 9).

**Fig. 5:**
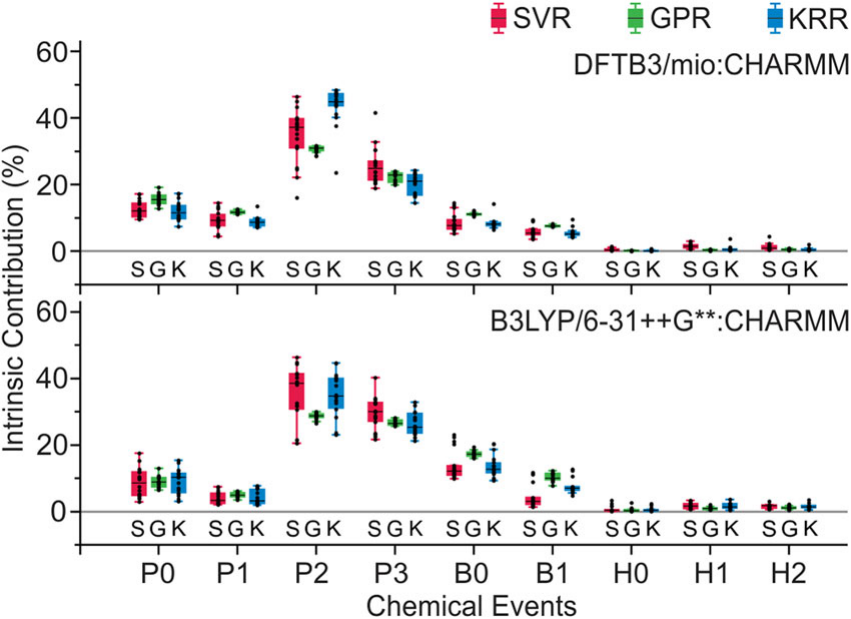
Intrinsic energy contribution. Intrinsic energy contribution measured on DFTB3/mio:CHARMM and B3LYP/6–31++G**:CHARMM reaction pathway profiles. The “S”, “G”, and “K” labels represent results from SVR, GPR, and KRR models, respectively. Each box contains *n* = 18 testing cases, the IQR noted by the boxes are divided by the median (black lines), and the whiskers mark the first datum that are larger than 1.5 * IQR. Joint contributions are measured for feature subgroups as defined in Supplementary Table 1. See also Supplementary Figs. 9, 10, and 143.

The intrinsic contribution provides a quantitative insight into the energy contribution of each chemical event to the overall energetic profile. The intrinsic energy contribution is calculated for all testing pathways from each level of QM theory and plotted in Fig. 5 and Supplementary Fig. 10. Generally, all regression models give the same statistical rankings of the energy contributions from each chemical event: P2 and P3 are the decisive processes during the reaction; P0, P1, B0, and B1 pose less impact to the overall energetic; Hydrogen bonds (H0, H1, and H2) are considered to be the least critical events. The intrinsic energy contribution measured using the GPR model is the most numerically stable, whereas the SVR model gives the largest deviation among the testing cases. As for pathway profiles decomposed at different QM levels of theory, the intrinsic contributions are compatible to each other.

### Reaction pathway geometry analysis

For the model system in this study, previous computational studies^17,18^ have demonstrated the detailed energetic landscape of the two concerted dual-proton transfer processes before and after the tetrahedral formation. Yet those works have reported different data on the height of stepwise activation barriers or the thermal stability of meta-stable intermediates. Herein, we revisited the acylation of TEM-1/benzylpenicillin catalysis and attempt to provide an explanation to the origin of this deviation. We have shown that the DFTB3/mio:CHARMM optimized reaction pathways gave unreliable configurational changes during the acylation pathway; thus the following analysis focus on the B3LYP optimized pathways.

**Fig. 6:**
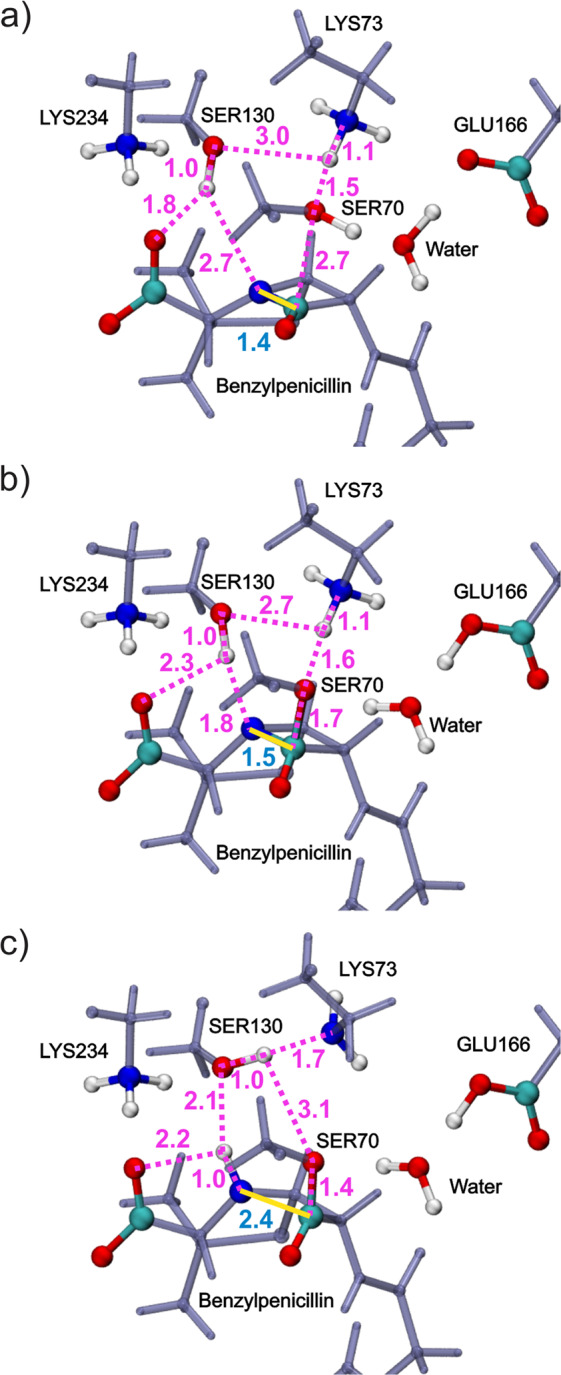
Interatomic distances averaged over all B3LYP/6–31G:CHARMM optimized pathways at each state. **a** Michaelis complex reactant; **b** Tetrahedral intermediate; **c** Acyl-enzyme product. Spheres in white, cyan, red, and blue represent H, C, O, and N atoms, respectively. The β-lactam scissile bond is marked by the yellow solid line and its bond length are noted in blue.

Upon the initial reactant state, the hydroxyl group of Ser130 is positioned within hydrogen bonding distances of the β-lactam nitrogen and the penicillin carboxylate group, with an average distance of 1.8 and 2.7 Å, respectively (Fig. 6a). During Ser70 addition (Fig. 6b), the hydroxyl group on Ser130 is also activated and moves closely to the thiazolidine nitrogen. The Ser130 proton that migrates to β-lactam nitrogen is then readily activated and eventually cleaves the scissile β-lactam bond (Fig. 6c). The conformational changes evidently show that the proton transfer from Ser130 hydroxyl group to the β-lactam nitrogen is also concerted to the formation of the tetrahedral intermediate. Omitting this reaction coordinate (RC) from the PES scan during the tetrahedral formation would consequently yield different results, which depend on the starting configurations where the PES calculations were initialized.

The reaction pathways from the chain-of-states calculations demonstrate that the acylation mechanism with Glu166 as a general base undergoes a concerted four-proton transfer process. The energy barriers during the acylation are correlated and inseparable. Previous high-level QM/MM calculations showed that the rate determining step of the hydrolysis takes place during the acylation^20^, enabling the comparison between the acylation reaction energy barriers with the experimental rate of hydrolysis^51^. Despite its barrier-underestimating nature, our CoS B3LYP/6–31++G**:CHARMM calculated energy barriers give the best agreement with experimental values (Table 1). It should be pointed out that the exclusion of correlated RCs will not impact the general mechanistic insights from the above-mentioned studies^16–18^, as it will be shown that these pioneer works actually built their PES based on the chemical events with the highest energetic contribution at each stage of the acylation.

### Dynamic energy contribution

The intrinsic energy contribution reflects the overall energetic contribution of a certain chemical process to the energetic profile. Alternatively, a dynamic energy contribution along the reaction progress could be determined by the partial derivatives with respect to each feature subset. In this measurement, all 18 pathways were used as the training-validation set, and the dynamic contribution was computed at each replica. Such numerical importance is not chemically interpretable, since the dynamic energy contributions would actually be the correlation between the distance variance and the energy profile. In this case, disregarding the differences among the domain sizes of distance variables could misestimate the contribution measurement of those features with small variance. Practically, dynamic energy contributions on static hydrogen interactions (H0, H1, H2) would be significantly higher than those events with larger variable variance, such as proton transfers (P0, P1, P2, P3) or bond formation and cleavage (B0, B1). Therefore, the partial derivative must be scaled by a weighting factor *w* that balances the domain size of each feature in the variable space. In addition, the correlation among the features must also be considered to ensure that the perturbation is applied parallelly to the progress of the reaction profile, which is described by a local correlation matrix **Γ**.

In the present study, the dynamic energy contribution is defined as:

3 $$I_{{\mathrm{a}},{\mathrm{dynamic}}}^{({\mathrm{r}})} \,	= w^{({\mathrm{r}})} \cdot I_{{\mathrm{a}},{\mathrm{dynamic}}}^{({\mathrm{r}})} \ast \\ \, 	= \left| (f({{\mathbf{A}}^{({\mathrm{r}})} + {\mathbf{{{E}}}}^{({\mathrm{r}})}{\mathbf{{\Gamma} }}^{({\mathrm{r}})}}) - f({{\mathbf{A}}^{({\mathrm{r}})} - {\mathbf{{{E}}}}^{({\mathrm{r}})}{\mathbf{{\Gamma} }}^{({\mathrm{r}})}})) \right|,$$

where

4 $$w^{({\mathrm{r}})} = D({{\mathbf{A}}^{({\mathrm{r}})} + {\mathbf{{{E}}}}^{({\mathrm{r}})}{\mathbf{{\Gamma} }}^{({\mathrm{r}})},{\mathbf{A}}^{({\mathrm{r}})} - {\mathbf{{{E}}}}^{({\mathrm{r}})}{\mathbf{{\Gamma} }}^{({\mathrm{r}})}})$$

5 $$I_{{\mathrm{a}},{\mathrm{dynamic}}}^{({\mathrm{r}})} \ast = \frac{{\left| {\left( {f\left( {{\mathbf{A}}^{({\mathrm{r}})} + {\mathbf{{{E}}}}^{({\mathrm{r}})}{\mathbf{{\Gamma} }}^{({\mathrm{r}})}} \right) - f\left( {{\mathbf{A}}^{({\mathrm{r}})} - {\mathbf{{{E}}}}^{({\mathrm{r}})}{\mathbf{{\Gamma} }}^{({\mathrm{r}})}} \right)} \right)} \right|}}{{D\left( {{\mathbf{A}}^{({\mathrm{r}})} + {\mathbf{{{E}}}}^{({\mathrm{r}})}{\mathbf{{\Gamma} }}^{({\mathrm{r}})},{\mathbf{A}}^{({\mathrm{r}})} - {\mathbf{{{E}}}}^{({\mathrm{r}})}{\mathbf{{\Gamma} }}^{({\mathrm{r}})}} \right)}}$$

*w*^(r)^ and ${\mathbf{A}}^{\left( {\mathrm{r}} \right)}$ are the weighing factor and the feature vector at the r-th replica, respectively; *D*(**A**, **B**) is the Euclidean distance between feature vectors **A** and **B**; ${\mathbf{{\Gamma} }}^{({\mathrm{r}})}$, the local correlation matrix at r-th replica, is defined as

6 $${\mathbf{{\Gamma} }}^{({\mathrm{r}})} = \left[ {\begin{array}{*{20}{c}} {\gamma _1^{\left( {\mathrm{r}} \right)}} & \cdots & 0 \\ \vdots & \ddots & \vdots \\ 0 & \cdots & {\gamma _{\mathrm{n}}^{\left( {\mathrm{r}} \right)}} \end{array}} \right],$$

7 $$\gamma _{\mathrm{i}}^{\left( {\mathrm{r}} \right)} = \left\{\begin{array}{*{20}{ll}} - 1,& \frac{{\partial f\left( {{\mathbf{A}}^{\left( {\mathrm{r}} \right)}} \right)}}{{\partial a_{\mathrm{i}}^{\left( {\mathrm{r}} \right)}}}\, <\, 0\;and\;a_{\mathrm{i}}^{\left( {\mathrm{r}} \right)} \in {\mathbf{a}}^{\left( {\mathrm{r}} \right)} \\ 0,&\!\!\!\!\!\! \frac{{\partial f\left( {{\mathbf{A}}^{\left( {\mathrm{r}} \right)}} \right)}}{{\partial a_{\mathrm{i}}^{\left( {\mathrm{r}} \right)}}} = 0\;or\;a_{\mathrm{i}}^{\left( {\mathrm{r}} \right)} \notin {\mathbf{a}}^{\left( {\mathrm{r}} \right)} \\ 1,& \frac{{\partial f\left( {{\mathbf{A}}^{\left( {\mathrm{r}} \right)}} \right)}}{{\partial a_{\mathrm{i}}^{\left( {\mathrm{r}} \right)}}} \,> \, 0\;and\;a_{\mathrm{i}}^{\left( {\mathrm{r}} \right)} \in {\mathbf{a}}^{\left( {\mathrm{r}} \right)} \end{array} \right.,$$

where **a**^(r)^ is the subset of features whose contribution is to be measured at r-th replica. The perturbation at r-th replica, **E**^(r)^, is defined as

8 $${\mathbf{{{E}}}}^{({\mathrm{r}})} = \left[ {\begin{array}{*{20}{c}} {\varepsilon _1^{\left( {\mathrm{r}} \right)}} & \cdots & {\varepsilon _{\mathrm{n}}^{\left( {\mathrm{r}} \right)}} \end{array}} \right],$$

9 $$\varepsilon_{\mathrm{i}}^{\left( {\mathrm{r}} \right)} = \left\{ \begin{array}{*{20}{ll}} 0, \hfill& a_{\mathrm{i}}^{\left( {\mathrm{r}} \right)} \notin {\mathbf{a}}^{\left( {\mathrm{r}} \right)} \\ p\left( {a_{\mathrm{i}}^{\left( {{\mathrm{r}} + 1} \right)} - a_{\mathrm{i}}^{\left( {{\mathrm{r}} - 1} \right)}} \right),&a_{\mathrm{i}}^{\left( {\mathrm{r}} \right)} \in {\mathbf{a}}^{\left( {\mathrm{r}} \right)} \end{array} \right..$$

The *p* value stands for the amount of perturbation applied. In this study, *p* is set as 0.01.

**Fig. 7:**
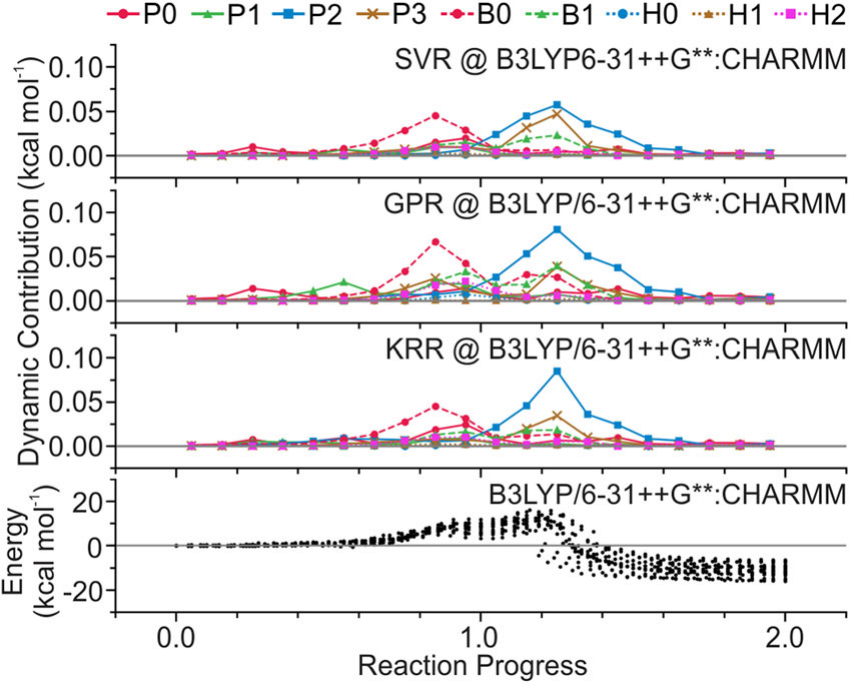
Dynamic energy contribution. The dynamic energy contribution measured from SVR, GPR and KRR regression models trained on energetic profiles calculated at B3LYP/6–31++G**:CHARMM level of theory. The values provided are the average over the reaction progress.

The derived dynamic energy contribution could decompose the energy contribution from each chemical event with regard to the reaction progress. The reaction coordinates of the pathways are normalized using three anchor points: reactant as 0.0, tetrahedral intermediate as 1.0, and product as 2.0. It is noted that the dynamic measurement depends on the level of QM theory applied in the pathway optimizations. We therefore focus on the energetic profiles calculated at B3LYP/6–31++G**:CHARMM level of theory (Fig. 7).

The acylation is initialized by the proton transfer between Ser70 and the catalytic water (P0). During the first transition to the tetrahedral intermediate, the bond formation between Ser70 Oγ and carbonyl carbon (B0) is deemed to be the most energetic dominant event. Notably, the protonation of the thiazolidine nitrogen (P3) is concerted in this process. The rate determining events of the acylated product formation are the dual protonation of Ser130 (P2) and the β-lactam nitrogen (P3) together with the cleavage of the β-lactam scissile bond (B1). In addition, the dynamic contribution measurement is also regression model-dependent, which in turn reflects the difference among the predictive PES of the regression models. Despite the differences among regression models, different levels of QM theory generally lead to the same conclusion, as illustrated in Supplementary Figs. 11–16.

During the formation of tetrahedral intermediate, the rate determining event is shown to be the bond formation between Ser70 hydroxyl oxygen and the β-lactam carbonyl carbon. As for the formation of acyl-enzyme product, the dual-proton transfer from Lys73 to β-lactam nitrogen, bridged by the Ser130 hydroxyl group, becomes the rate determining event. The dynamic energy contributions are consistent with the intrinsic contribution measurements as they identify the same critical chemical events during the acylation. Generally, the dynamic energy contribution qualitatively reveals the time windows and spans of chemical events and quantitatively reflects their underlying correlations.

## Discussion

In this study, we presented machine-learning-based theoretical models to predict the energetic profiles for enzymatic reactions. Two numerical measurements based on chemical events were developed and provided insights into the underlying mechanisms of the reaction.

Via the intrinsic energy contribution, the proton transfer between Lys73 and Ser130 and the protonation of the thiazolidine nitrogen are deemed to be the most energetically significant chemical events, while the Glu166 proton accepting is revealed to be less essential. In this regard, there are two major factors that determine whether the investigated acylation pathway is viable: the presence of the fully protonated Lys73 as the proton source to re-protonate Ser130; and the existence of the hydroxyl group in Ser130, which serves as the proton bridge during the tetrahedral collapsing. Such evidence could be connected to experimental mutagenesis studies. It has been shown that Lys73 mutant of TEM-1 deactivated the enzyme for hydrolysis^10^, indicating that acylation pathway is turned off in the absence of a proton source to Ser130. Based on our analysis, it is obvious that the acylation pathway is prohibited as its energetic determining event (P2) is no longer accessible in the reaction space. In the case of Ser130Gly mutant of TEM-1 (or namely TEM-76), Thomas et al.^11^ demonstrated that the enzyme was still hydrolysis-active as the Ser130 hydroxyl group is substituted by a crystal water. Of course, the catalytic rate of the mutated TEM-1 is decreased due to the relatively lower reactivity of water molecule. Moreover, the important role of Glu166 has been emphasized in many experimental studies for the hydrolysis. Mutations on this residue could turn class A β-lactamases into a penicillin-binding-protein (PBP)^5,6^, suggesting that the acylation process is still thermodynamically favorable. Our analysis also aligns with this evidence, as the only process (P1) that involves Glu166 is deemed to be non-essential.

No direct experimental measurement could be adopted to validate the dynamic energy contribution measurements as any chemical process could not be simply isolated from the reaction. However, qualitative agreement with the intrinsic contribution assessment is observed. The underlying correlations between the proton transfers are validated in the dynamic energy contribution. Notably, the protonation of Ser130 hydroxyl group and the thiazolidine nitrogen are found to be concerted with the formation of tetrahedral intermediate, indicating that the acylation reaction is a one-step 4-proton-transfer process. Isolating such proton transfers from the tetrahedral formation process has led to conflicted estimations on the overall reaction barrier or the stepwise activation energy (Table 1). Moreover, dynamic energy contributions reveal that the rate limiting events of the acylation are the proton transfers from Lys73 to β-lactam nitrogen via the bridging Ser130 hydroxyl group, opposing to previous QM/MM calculations^17,18^, in which the tetrahedral formation is concluded to be the rate limiting step.

It should be emphasized that the present study serves as a further complement, not criticism, to previous high-level insightful QM/MM computational studies^16–18^ on the mechanisms of β-lactamases driven antibiotic resistance (see also Supplementary Note 1).

Owing to the complexity and high degrees of freedom of reaction environment, entropy inevitably plays an important role in enzymatic functions^52,53^. There are many different entropic contributions and penalties in enzymatic catalysis^54^. It is generally accepted that the translational and rotational entropy penalties for the ligand binding have already been paid upon the formation of enzyme-ligand complex^53,54^. The remaining entropic factors for enzymatic catalysis should mainly stem from the intrinsic properties of the catalytic systems. In principle, the current theoretical model could include significant part of the catalytic entropy effects. The current model was built based on a total of 18 MEPs starting from 18 representative protein conformations from the sampling in different functional states. The consideration of multiple pathways in the catalytic mechanism covers major entropic effects of actual transition being distributed among multiple possible pathways. In addition, this model should partially account for the entropic factors of the protein-ligand “snug fit” binding, in which the ligand is locked at the binding pocket of enzyme with reacting groups of ligand and enzyme in the right position and orientation for reactions. There are certainly many more important entropic factors for enzymatic reactions that the proposed model in this study could not fully account for. For example, some proteins may carry special entropic property under extreme circumstances, such as psychrophilic and thermophilic proteins^55,56^. With continuous efforts from the methodology developing community, searching for novel robust and accurate enhanced sampling approaches that appropriately account for the entropic contribution to catalysis from protein flexibility and other factors remains an active field^57,58^.

Further comments are noted on the transferability and extensibility of the proposed approach. Whereas the proposed energy contribution measurements are derived without introducing any model-dependent precondition, they are naturally transferable to other enzymatic processes as well. It should be noted that for many complex systems, such as transition metal-based enzymes, significant developments may be necessary to apply the proposed approaches on these systems effectively.

As for the methodology aspect, any configuration space sampling method that could cover the overall reaction progress should be suitable for the regression models. The constraints or restraints applied to control the replica distribution in the CoS methods should not affect the regression performance. Different regression models, including high-level ensemble-based machine learning methods (e.g., neural networks, regression trees, boosting methods, etc.) are also viable, as the proposed energy contribution decomposing approaches are universal measurements. A diverse choice of input features that could bridge chemical properties with reaction progress should also be suitable for the proposed models, including other generalized coordinates systems.

In summary, we presented novel regression models with machine-learning component to quantify the energetic contributions from, as well as the correlations among, individual chemical process during enzyme catalysis with high degrees of freedom. Such quantitative measurements serve as a useful energetic-decomposing analysis to the enzymatic reaction pathway and reflect the detailed underlying mechanism. This study also serves as a proof of the concept for extending the application of machine-learning techniques to probe complex enzymatic reaction mechanisms in high degrees of freedom configurational space.

## Methods

### QM/MM calculations

All hybrid QM/MM multiscale calculations in the present study were conducted by interfacing CHARMM^40^ with SCC-DFTB^41,42^ or Q-Chem 5.0^59^. All MD simulations were performed by OpenMM 7.3.1^60^. The acyl-enzyme product of TEM-1 with benzylpenicillin was obtained from the X-ray crystal structure (PDB id: 1fqg)^6^ and the mutant residue Asn166 was modified to Glu166 as in the wild type TEM-1. The residues were then protonated according to previous studies^15^. The system was solvated in a 77 Å cubic water box. Sodium and chloride ions were added to balance the total charge of the system. In order to fully relax the system, classical mechanic minimization and equilibration were performed with the CHARMM36 force field^61^ for proteins, CHARMM general force field (CGenFF)^62^ for the penicillin molecule and TIP3P model^63^ for water. The structure of the QM/MM initial pathway calculation was taken from the trajectory of a 10 ns MD simulation at 300 K, as included in the Supplementary Data 1. The chain-of-states method, RPATh with constraints^36,37^ as implemented in CHARMM, was applied for reaction pathway calculations. All the pathway calculations were carried out with 50 replicas. The parallel-distributed replica (REPDSTR) computational framework^64^ implemented in CHARMM was employed in the B3LYP pathway optimizations to accelerate the calculation.

### Reaction pathway sampling

The initial pathway was calculated from DFTB3/mio:CHARMM level of theory with any residue in the outer 15 Å of QM region selected as the unfrozen MM region (Supplementary Fig. 1). Based on the initial pathway, multiple reaction pathways were sampled. Firstly, three replicas representing reactant (r), transition (t), and product (p) states were selected. In all, 200 ns MD simulations were performed on each of the selected replicas. During the MD runs, all the atoms in the QM region were fixed and snapshots were taken every 0.1 ps. 2-Dimensional principal component analysis (2D-PCA) was performed on the MD trajectories with the pairwise Cα distances as input. The 2D-PCA result was grouped into 6 clusters by the Agglomerative Clustering method, and the snapshots that are the closest to the centers of each cluster were chosen as the representative structures (Supplementary Fig. 2). A total of 18 representative structures were then selected. In order to retain the consistency among the QM/MM pathway’s energetic profiles, a common MM region was used, and was selected to be the union set of residues within the outer 10 Å of all representative QM regions. Geometry optimizations were then performed on the selected representative structures. Lastly, based on those representative structures, 18 RPATh with constraints calculations were carried out to obtain the MEPs. The coordinates of initial QM/MM configurations, the initial pathway, and the optimized pathways (denoted in “ID/state”) are included in the Supplementary Data 1.

### Machine-learning protocols

The scikit-learn package^65^ was employed for various machine-learning protocols, including dimensionality reduction, clustering, and regression. The hydrogen bonding interactions are identified via the Baker-Hubbard criteria as implemented in MDTraj 1.9.3^66^. The radial basis function (RBF) was used as the kernel function for all regression models: support vector regression (SVR)^48^, Gaussian process regression (GPR)^49^, and kernel ridge regression (KRR)^50^. The training and testing datasets used for prediction performance benchmarking, intrinsic and dynamic energy contribution measurements are summarized in Supplementary Table 2. For the training-validation process of models, the leave-one-group-out cross-validation (LOGO CV) regression analysis was employed in the validation step; the hyper-parameters of the models were tuned via a grid search (GS) strategy. In addition, as GPR is prone to overfit on small datasets, the noise level *α*, which is enclosed in a white kernel function, is also refined to obtain a reasonable model. The calculated pathway and the predicted pathway profiles are included in Supplementary Figs. 17–142.

## Supplementary information

Supplementary Information

Description of Additional Supplementary Files

Supplementary Data 1

## Data Availability

The authors declare that all data supporting the findings of this study are available within the paper and its Supplementary Information files.

## Code availability

CHARMM c42b2 (with REPDSTR support) and Q-Chem 5.0 are commercial software and are accessible from their distributor. SCC-DFTB is distributed as a build-in module of CHARMM c42b2. MDTraj 1.9.3, OpenMM 7.3.1, and scikit-learn are freeware and are accessible from the reference provided in the text. The Python codes developed to derive the intrinsic and dynamic energy contributions in this study are available on GitHub at github.com/smutaogroup/tem1_acylation_pathfitting.
