# Supplementary material for: Unraveling the energetic significance of chemical events in enzyme catalysis via machine-learning based regression approach
Source: Commun Chem. 2020 Oct 8;3:134. doi: 10.1038/s42004-020-00379-w (PMC9814854; doi:10.1038/s42004-020-00379-w)
Supplement: Supplementary file 1 — Supplementary Information [file 42004_2020_379_MOESM1_ESM.pdf]

Unraveling the Energetic Significance of Chemical Events in Enzyme Catalysis via Machine-Learning based Regression Approach

- Supplementary Information -

Zilin Song,[1] Hongyu Zhou,[1] Hao Tian,[1] Xinlei Wang,[2] and Peng Tao [1,*]

[1] Department of Chemistry, Center for Research Computing, Center for Drug Discovery, Design, and Delivery (CD4), Southern Methodist University, Dallas, Texas 75275, United States

[2] Department of Statistical Science, Southern Methodist University, Dallas, Texas 75275, United States

***** Correspondence: ptao@smu.edu

Table of Contents

Supplementary Table 1 1

Supplementary Table 2 2

Supplementary Figure 1 3

Supplementary Figure 2 4

Supplementary Figure 3-6 5

Supplementary Figure 7 9

Supplementary Figure 8 10

Supplementary Figure 9, 10 11

Supplementary Figure 11-16 14

Supplementary Figure 17-142 20

Supplementary Figure 143 62

Supplementary Note 1 65

Supplementary Table 1

Supplementary Table 1. Notation of feature groups and the corresponding chemical events.

Notation	Features	Explanation
P0	d0, d2	Proton transfer from Ser70 Oγ to catalytic water
P1	d9, d10	Proton transfer from catalytic water to Glu166 Oε
P2	d3, d4, d5	Proton transfer from Lys73 Nζ to SER130 Oγ
P3	d6, d7, d8	Proton transfer from Ser130 Oγ to benzylpenicillin N4
B0	d1	Bond formation between benzylpenicillin C7 and Ser70 Oγ
B1	d14	Bond cleavage between benzylpenicillin C7 and N4
H0	d11	Hydrogen bonding between catalytic water and Asn170
H1	d12	Hydrogen bonding between Asn170 and Glu166
H2	d13	Hydrogen bonding between Ser235 and benzylpenicillin O12

Supplementary Table 2

Supplementary Table 2. Component of datasets used in the training-validation and testing process for various purpose in the current study.

Notation of the model [a]	Data used in the training/testing set	
	Replica structures ('x')	Replica energies ('y')
Benchmarking the prediction quality (Figure 3d) [b]		
DFTB3/mio:CHARMM	DFTB3/mio:CHARMM	DFTB3/mio:CHARMM
B3LYP/6-31G:CHARMM	B3LYP/6-31G:CHARMM	B3LYP/6-31G:CHARMM
B3LYP/6-31+G*:CHARMM	B3LYP/6-31G:CHARMM	B3LYP/6-31+G*:CHARMM
B3LYP/6-31++G**:CHARMM	B3LYP/6-31G:CHARMM	B3LYP/6-31++G**:CHARMM
B3LYP-D3/6-31++G**:CHARMM	B3LYP/6-31G:CHARMM	B3LYP-D3/6-31++G**:CHARMM
B3LYP/6-311++G**:CHARMM	B3LYP/6-31G:CHARMM	B3LYP/6-311++G**:CHARMM
B3LYP-D3/6-311++G**:CHARMM	B3LYP/6-31G:CHARMM	B3LYP-D3/6-311++G**:CHARMM
Intrinsic energy contribution (Figure 5, Supplementary Figure 9, 10) [b]		
DFTB3/mio:CHARMM	DFTB3/mio:CHARMM	DFTB3/mio:CHARMM
B3LYP/6-31G:CHARMM	B3LYP/6-31G:CHARMM	B3LYP/6-31G:CHARMM
B3LYP/6-31+G*:CHARMM	B3LYP/6-31G:CHARMM	B3LYP/6-31+G*:CHARMM
B3LYP/6-31++G**:CHARMM	B3LYP/6-31G:CHARMM	B3LYP/6-31++G**:CHARMM
B3LYP-D3/6-31++G**:CHARMM	B3LYP/6-31G:CHARMM	B3LYP-D3/6-31++G**:CHARMM
B3LYP/6-311++G**:CHARMM	B3LYP/6-31G:CHARMM	B3LYP/6-311++G**:CHARMM
B3LYP-D3/6-311++G**:CHARMM	B3LYP/6-31G:CHARMM	B3LYP-D3/6-311++G**:CHARMM
Dynamic energy contribution (Figure 7, Supplementary Figure 11 to 16) [c]		
DFTB3/mio:CHARMM	DFTB3/mio:CHARMM	DFTB3/mio:CHARMM
B3LYP/6-31G:CHARMM	B3LYP/6-31G:CHARMM	B3LYP/6-31G:CHARMM
B3LYP/6-31+G*:CHARMM	B3LYP/6-31G:CHARMM	B3LYP/6-31+G*:CHARMM
B3LYP/6-31++G**:CHARMM	B3LYP/6-31G:CHARMM	B3LYP/6-31++G**:CHARMM
B3LYP-D3/6-31++G**:CHARMM	B3LYP/6-31G:CHARMM	B3LYP-D3/6-31++G**:CHARMM
B3LYP/6-311++G**:CHARMM	B3LYP/6-31G:CHARMM	B3LYP/6-311++G**:CHARMM
B3LYP-D3/6-311++G**:CHARMM	B3LYP/6-31G:CHARMM	B3LYP-D3/6-311++G**:CHARMM

[a] Note that all B3LYP pathway profiles are single point energy refined from B3LYP/6-31G:CHARMM optimized pathway geometries;

[b] 17 pathways are used as the training-validation set, 1 pathway is used as the testing set;

[c] All 18 pathways are used as the training-validation set, no testing set is needed.

Supplementary Figure 1

Supplementary Figure 1. The initial DFTB3/mio:CHARMM pathway.

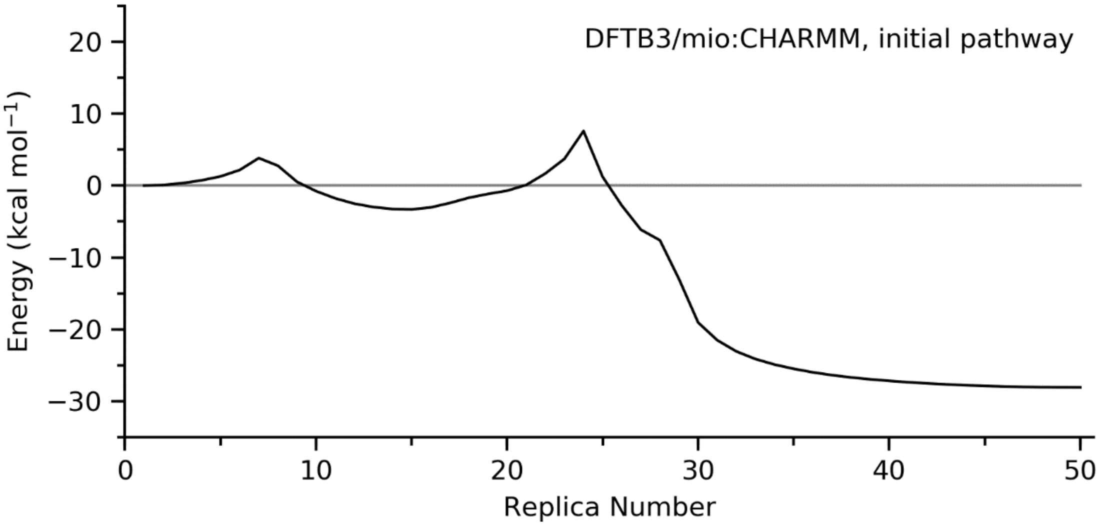

The QM states used for the pathway sampling is reactant (r, replica 1), transition (t, replica 24), and product (p, replica 50).

Supplementary Figure 2

Supplementary Figure 2. 2D-PCA dimensionality reduction results were clustered into 6 clusters by Agglomerative Clustering method.

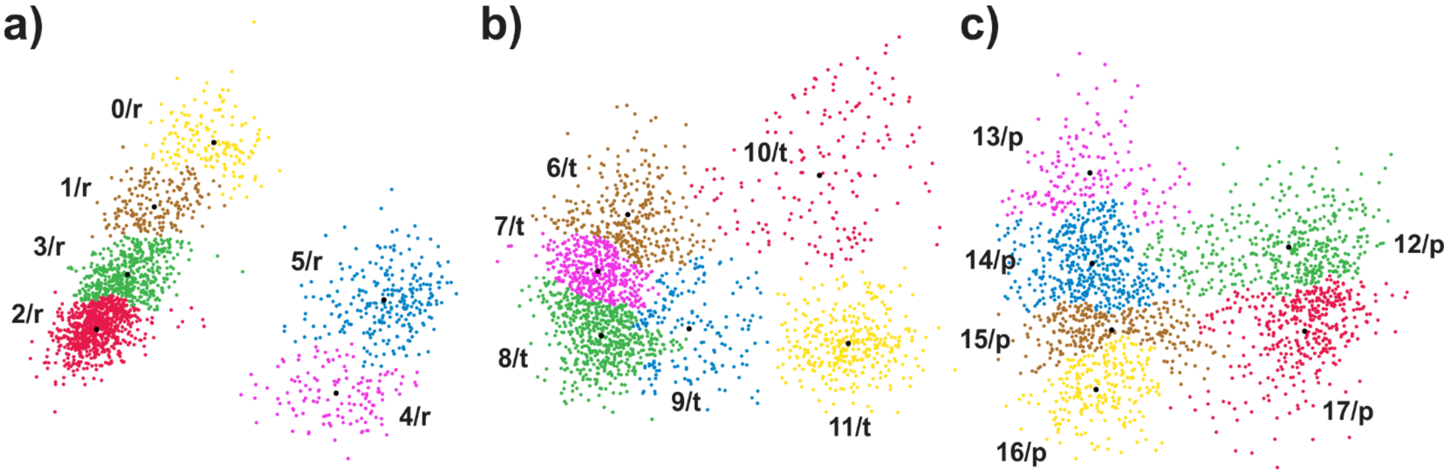

2D-PCA reduced pairwise Cα MD trajectory with QM region fixed in (a) reactant state; (b) transition state; (c) product state. The snapshots taken for pathway optimization are those in the center of each cluster, represented by the black dots.

Supplementary Figure 3-6

Supplementary Figure 3. Reaction pathway profiles from B3LYP/6-31G:CHARMM calculations.

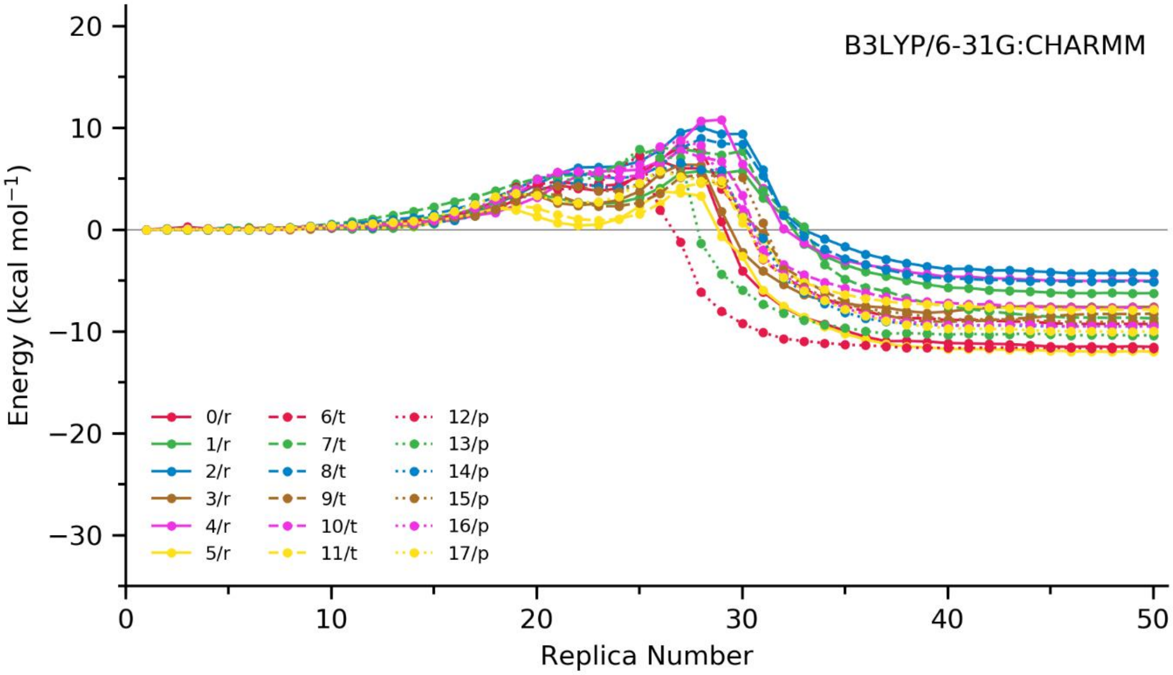

Supplementary Figure 4. Reaction pathway profiles from B3LYP/6-31+G*:CHARMM calculations.

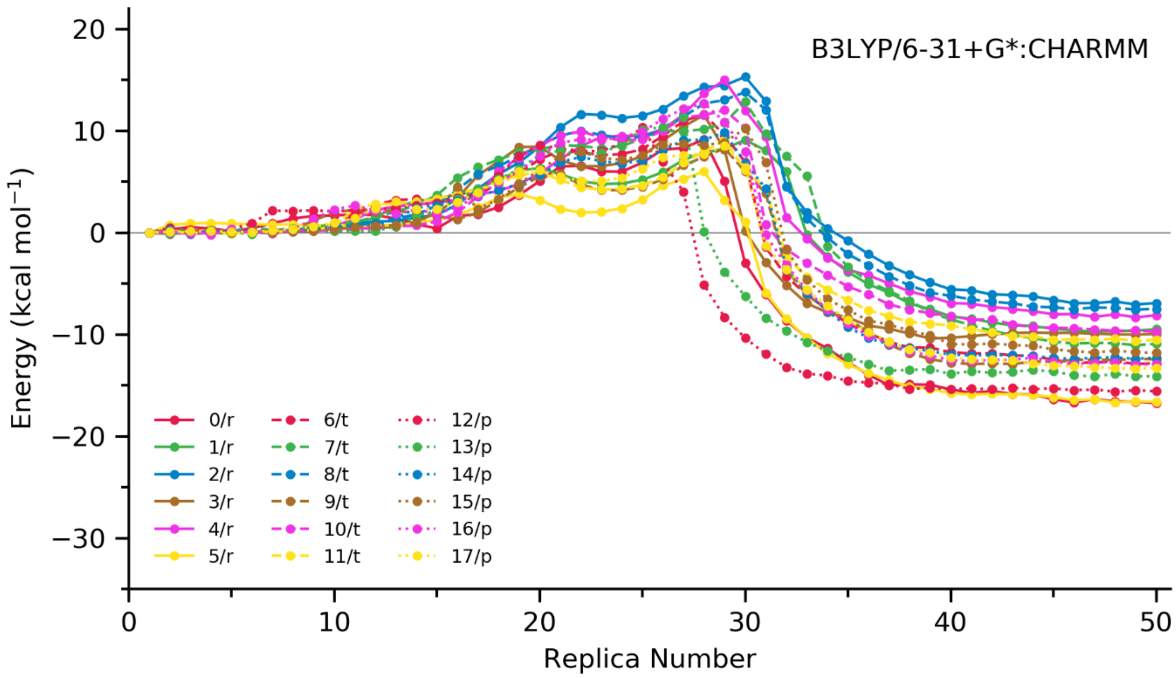

Supplementary Figure 5. Reaction pathway profiles from B3LYP/6-311++G**:CHARMM calculations.

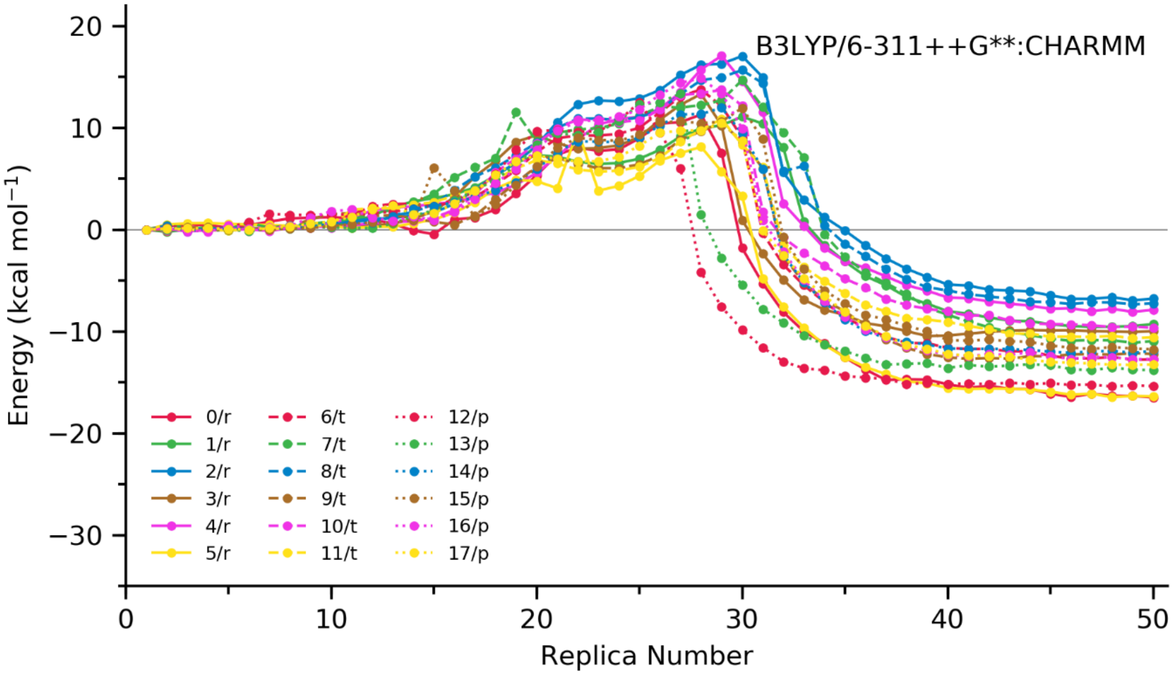

Supplementary Figure 6. Reaction pathway profiles from B3LYP-D3/6-311++G**:CHARMM calculations.

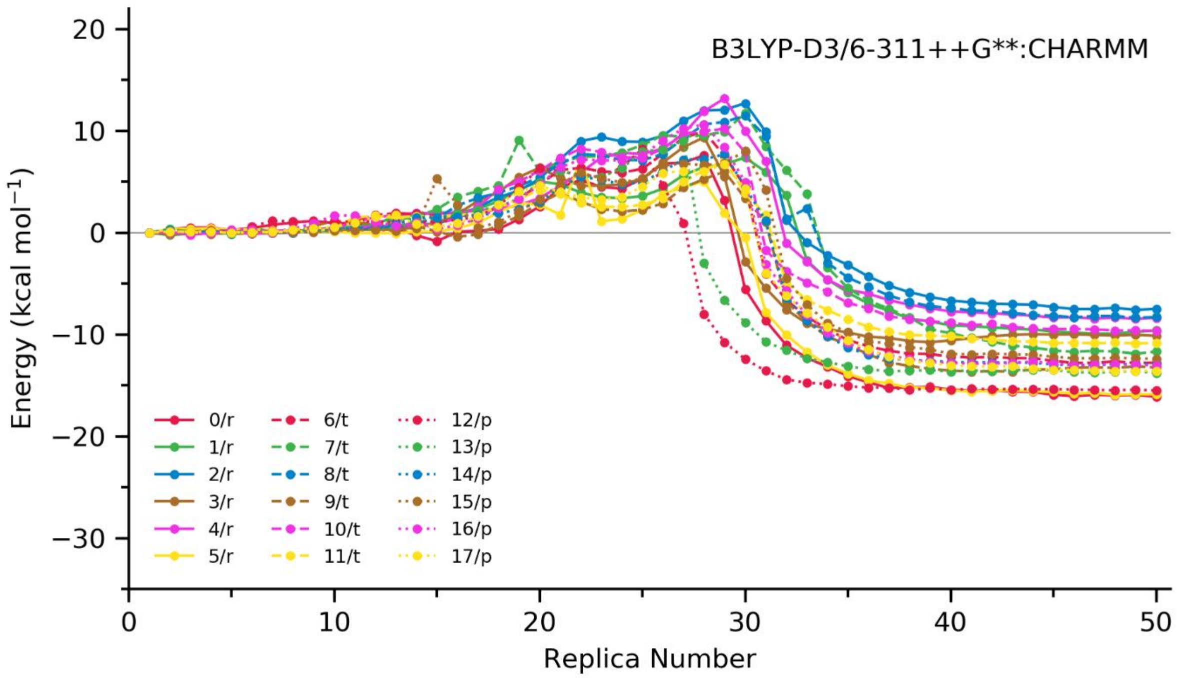

Supplementary Figure 7

Supplementary Figure 7. K-best feature selection results.

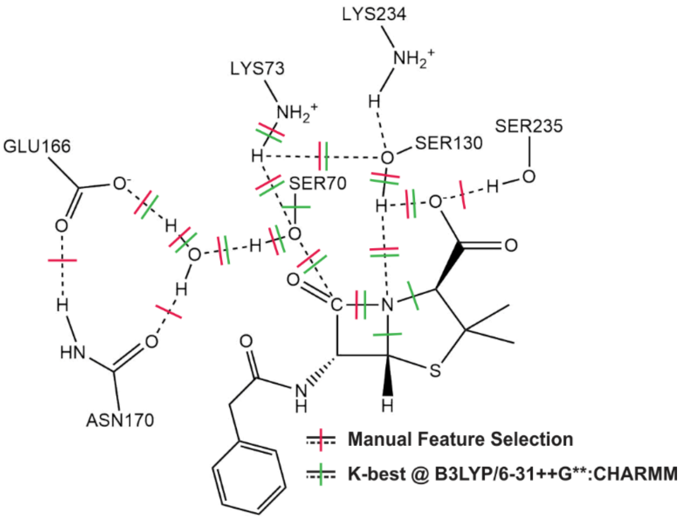

Feature selection results from the univariate K-best method based on mutual information between each independent-dependent variable pair. The top 15 features are presented.

Supplementary Figure 8

Supplementary Figure 8. Convergence test for three regression models.

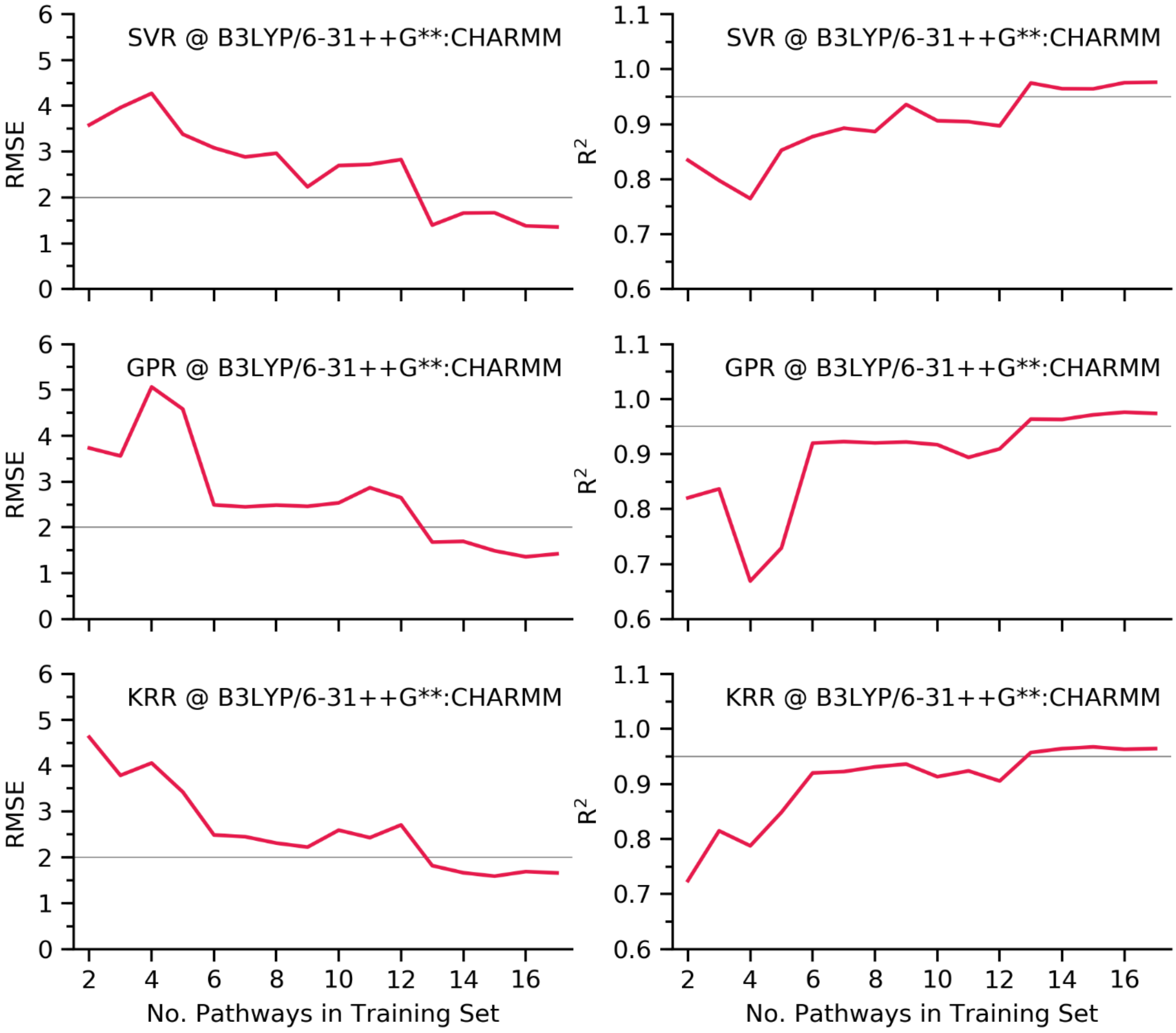

The convergence is determined by the quality of prediction, RMSE and R^2. If an ideally complete dataset was used to train the regression model, the regression model would produce 100% accurate prediction on any testing set. Therefore, the completeness could be assessed by the precision of the machine learning models. We conducted a convergence test on the B3LYP/6-31++G**:CHARMM dataset. The 0/r pathway is used as the testing set and multiple regression models was trained using the other 2 to 17 pathways. The performances of those models were benchmarked with the RMSE and R^2 between the calculated and predicted profile, as shown in Supplementary Figure 8. It is noted that all regression models converge well when 13 pathways are used as the training-validation set.

Supplementary Figure 9, 10

Supplementary Figure 9. The intrinsic energy contributions calculated directly from equation 2 (see main text).

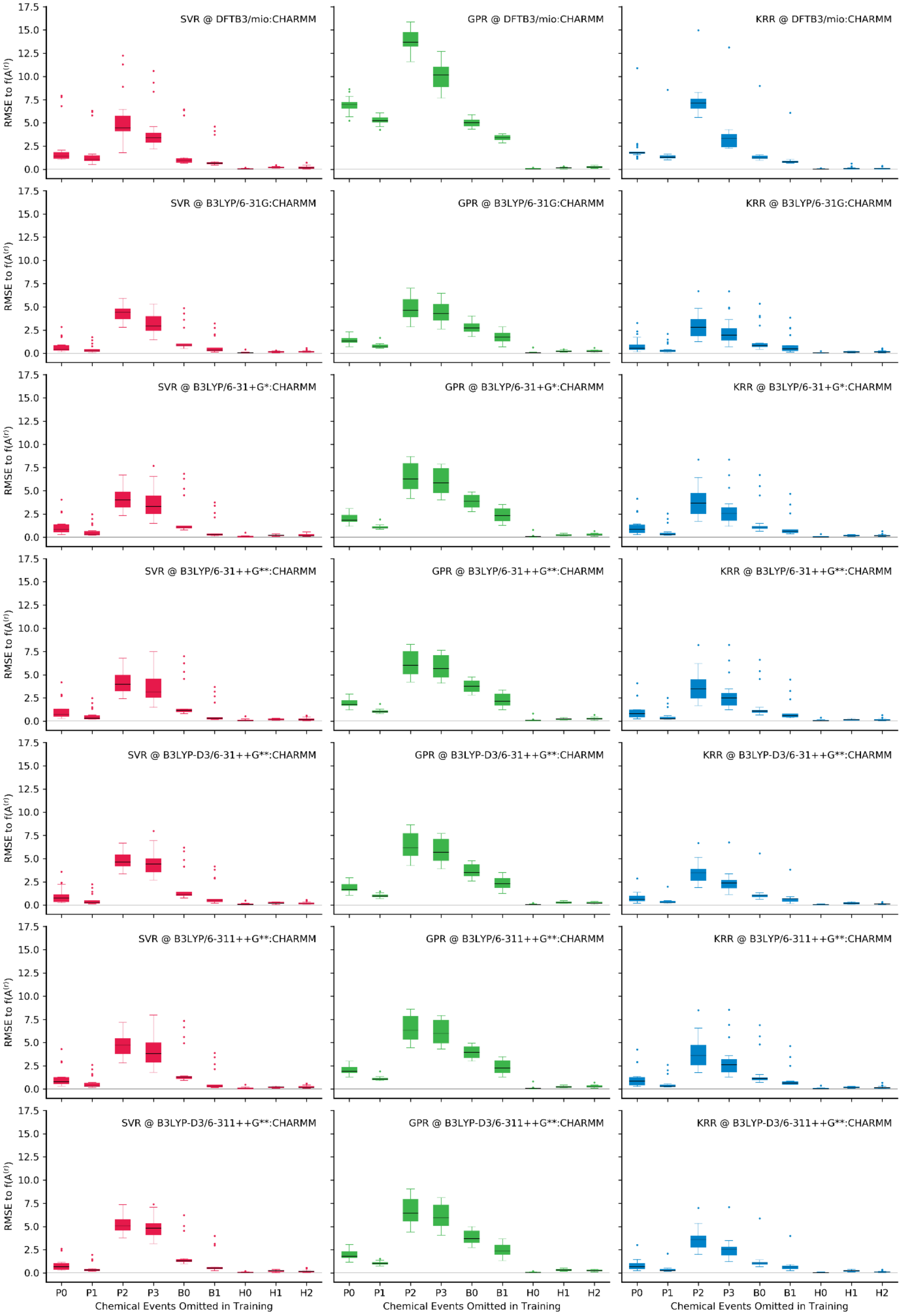

Each box contains n = 18 testing cases, the IQR noted by the boxes are divided by the median (black lines), and the whiskers mark the first datum that are larger than 1.5 * IQR. Joint contributions are measured for feature subgroups as defined in Supplementary Table 1.

Supplementary Figure 10. The % intrinsic energy contribution from other QM levels of theory.

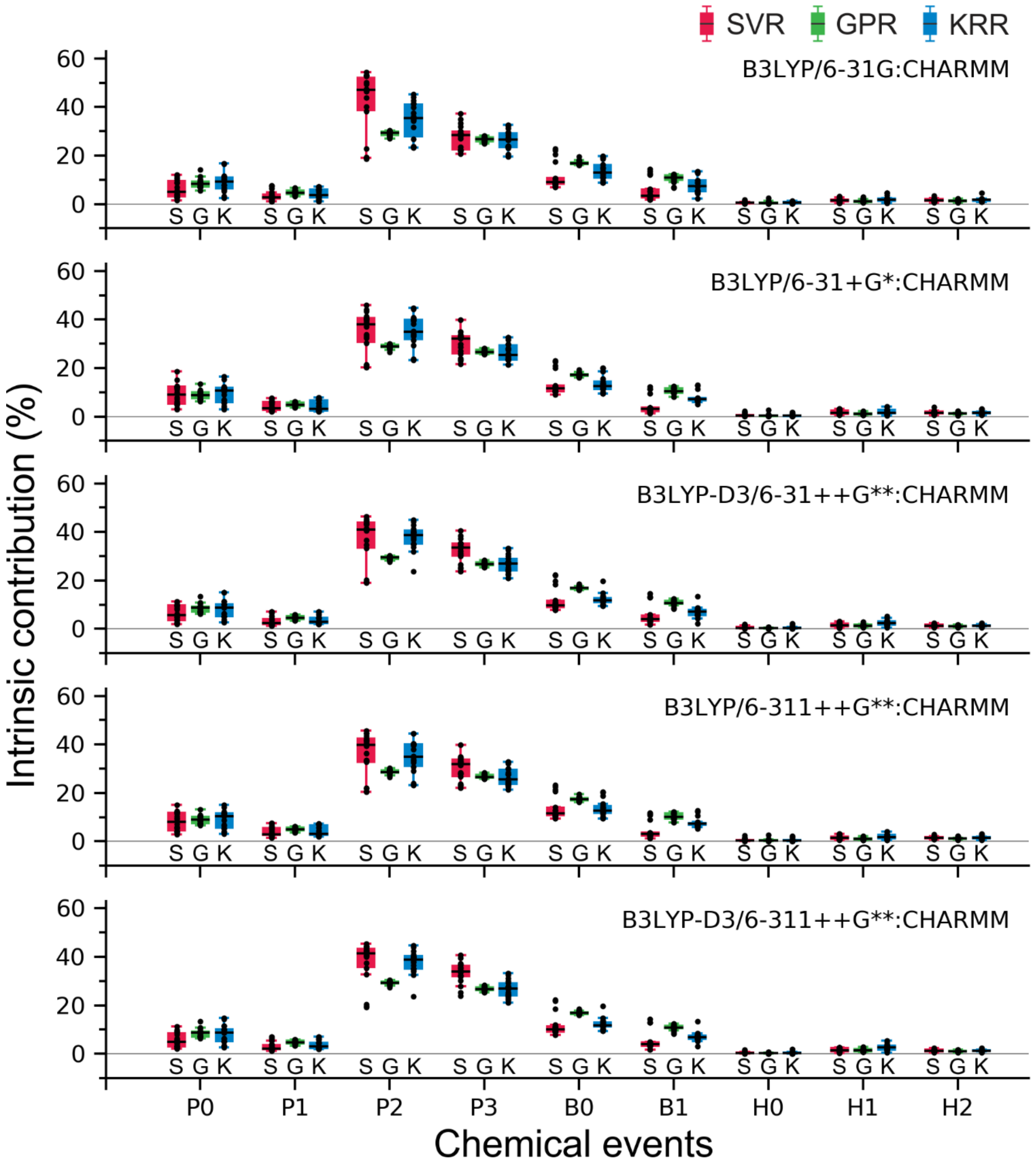

The ‘S’, ‘G’, and ‘K’ labels represent results from SVR, GPR, and KRR models, respectively. Each box contains n = 18 testing cases, the IQR noted by the boxes are divided by the median (black lines), and the mark the first datum that are larger than 1.5 * IQR. Joint contributions are measured for feature subgroups as defined in Supplementary Table 1.

Supplementary Figure 11-16

Supplementary Figure 11. Dynamic energy contribution from DFTB3/mio:CHARMM pathways.

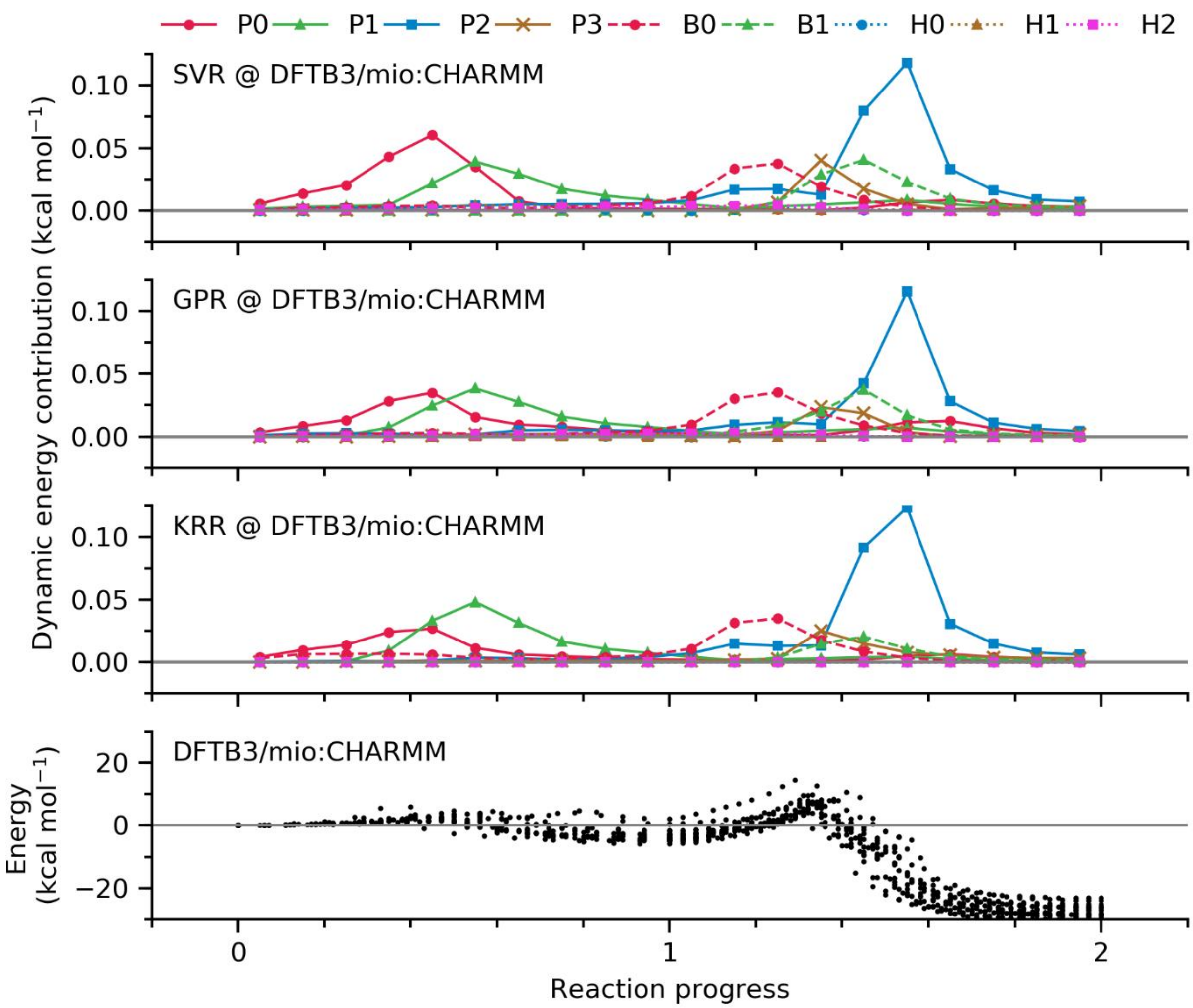

Supplementary Figure 12. Dynamic energy contribution from B3LYP/6-31G:CHARMM pathways.

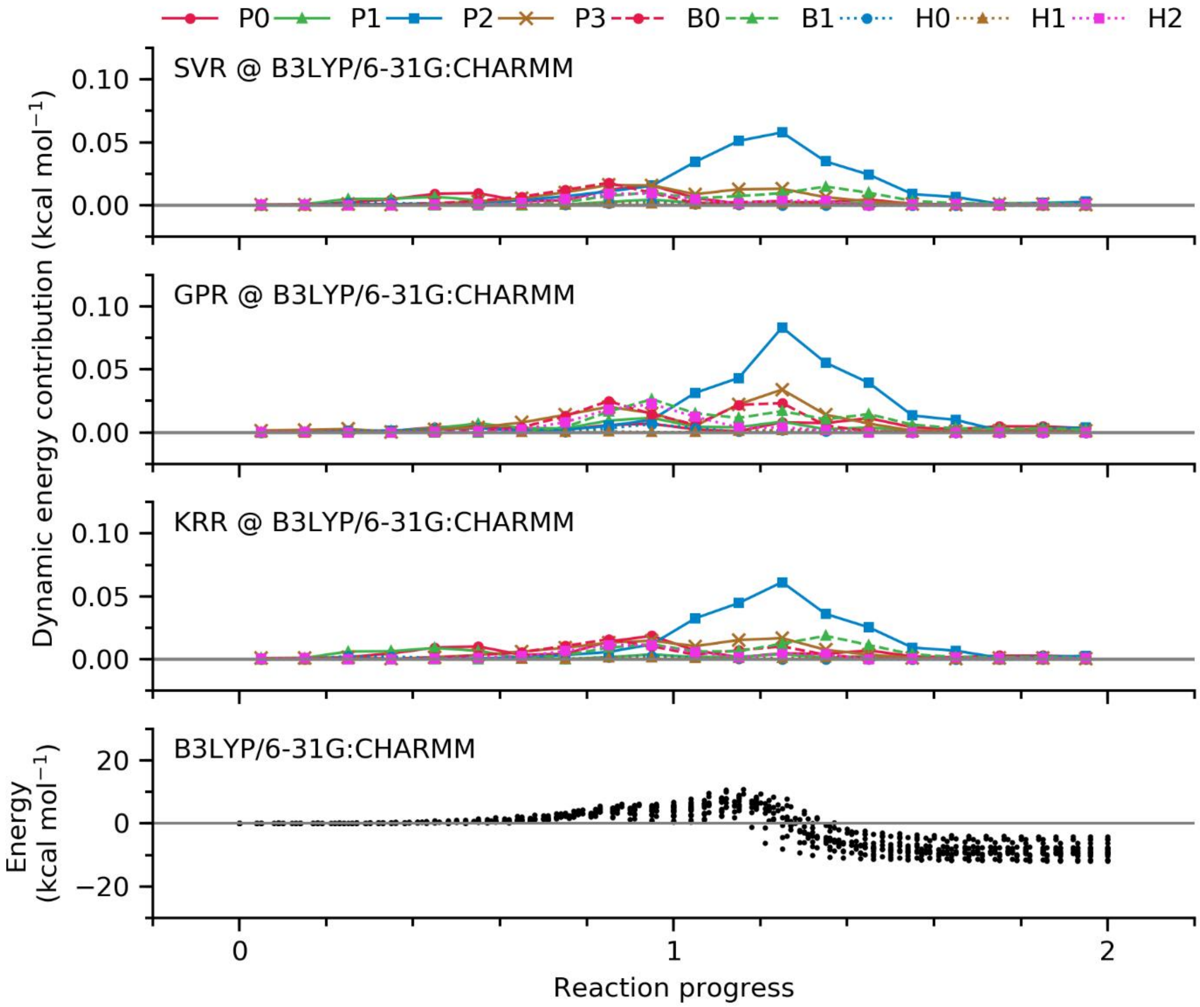

Supplementary Figure 13. Dynamic energy contribution from B3LYP/6-31+G*:CHARMM pathways.

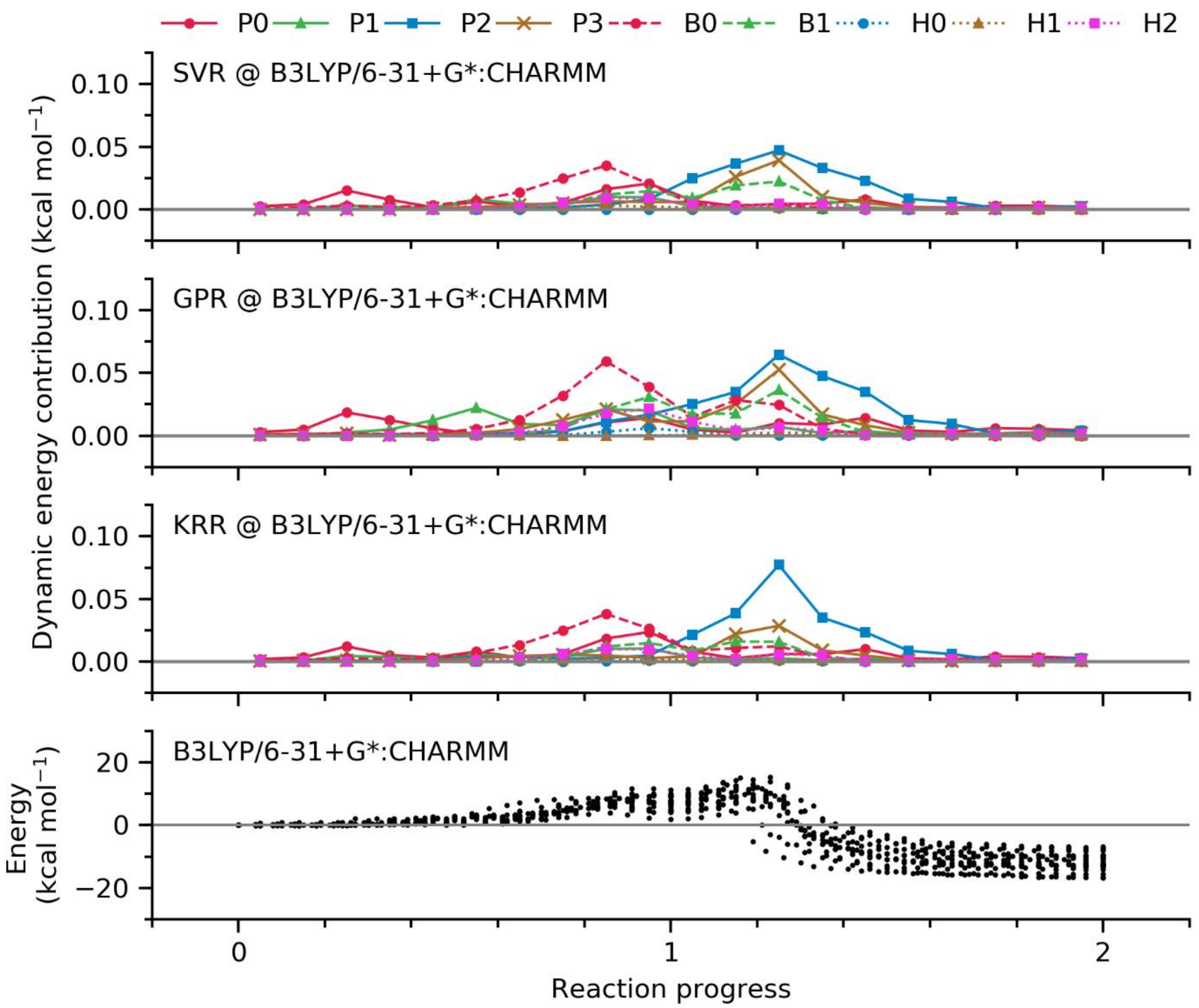

Supplementary Figure 14. Dynamic energy contribution from B3LYP-D3/6-31++G**:CHARMM pathways.

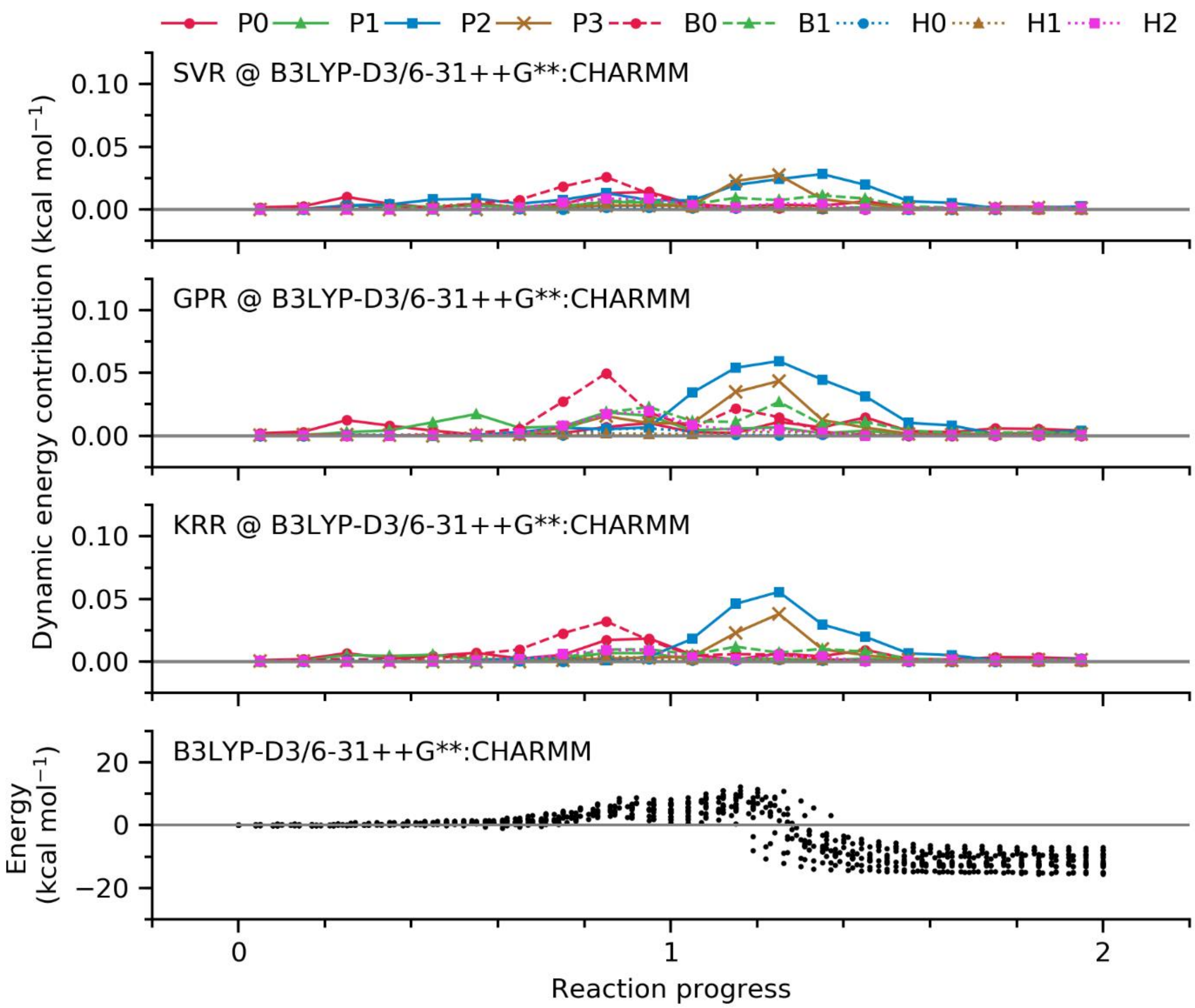

Supplementary Figure 15. Dynamic energy contribution from B3LYP/6-311++G**:CHARMM pathways.

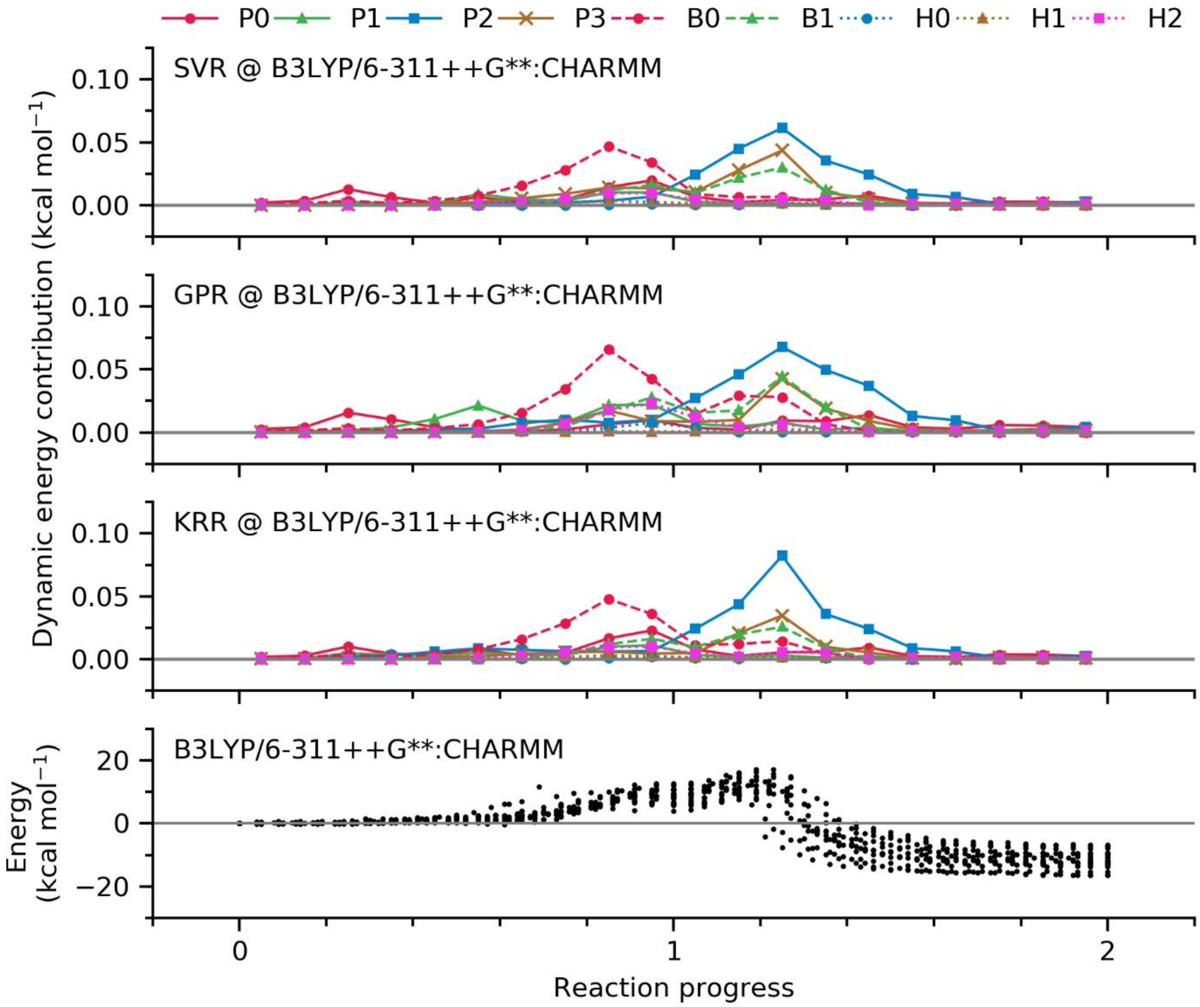

Supplementary Figure 16. Dynamic energy contribution from B3LYP-D3/6-311++G**:CHARMM pathways.

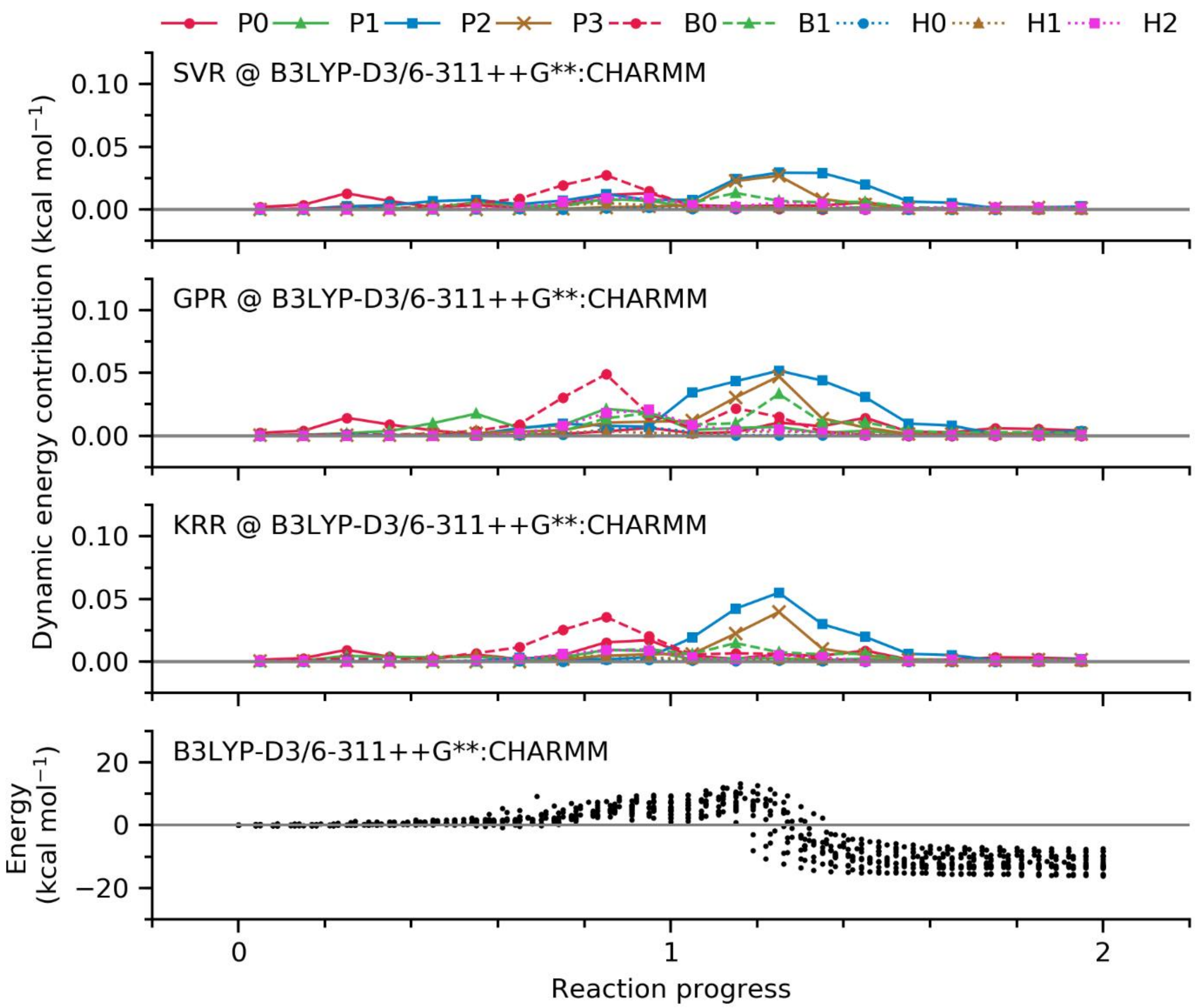

Supplementary Figure 17-142

Supplementary Figure 17 to 34. the DFTB3/mio:CHARMM pathways

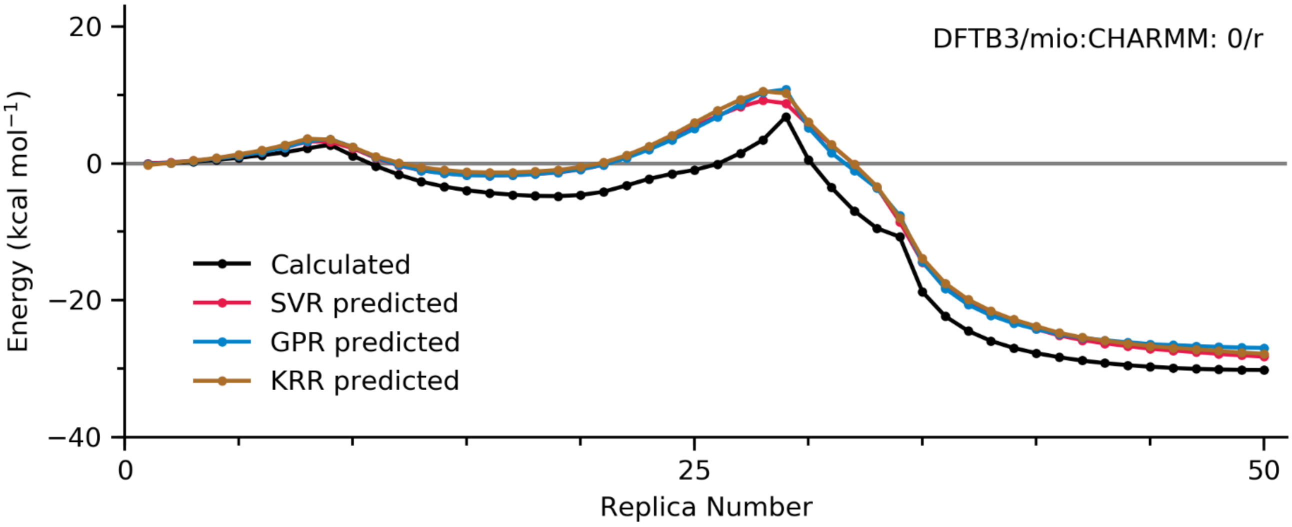

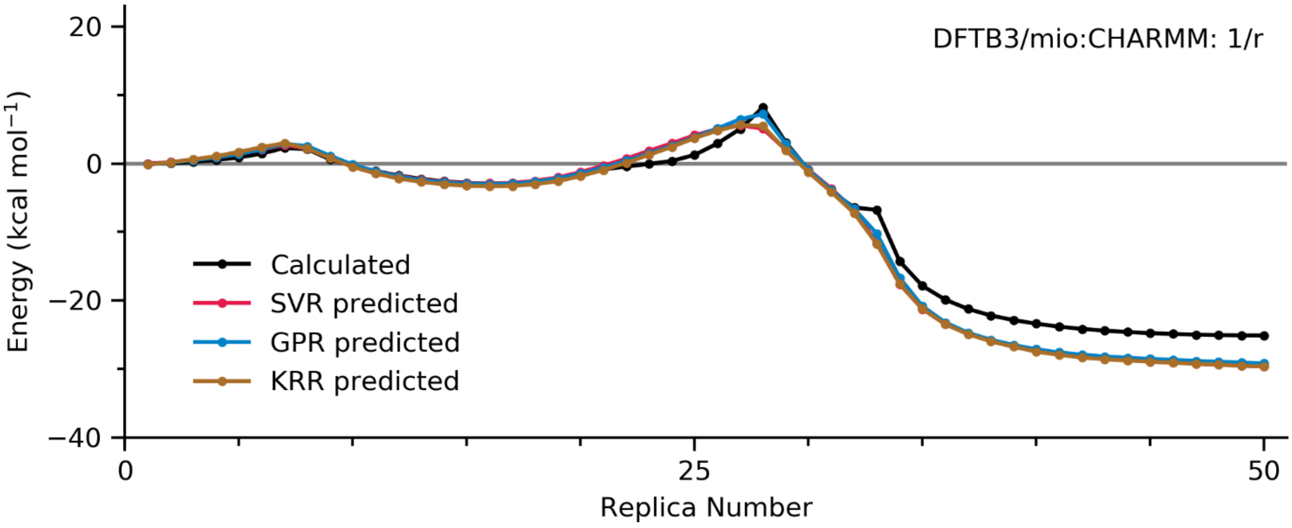

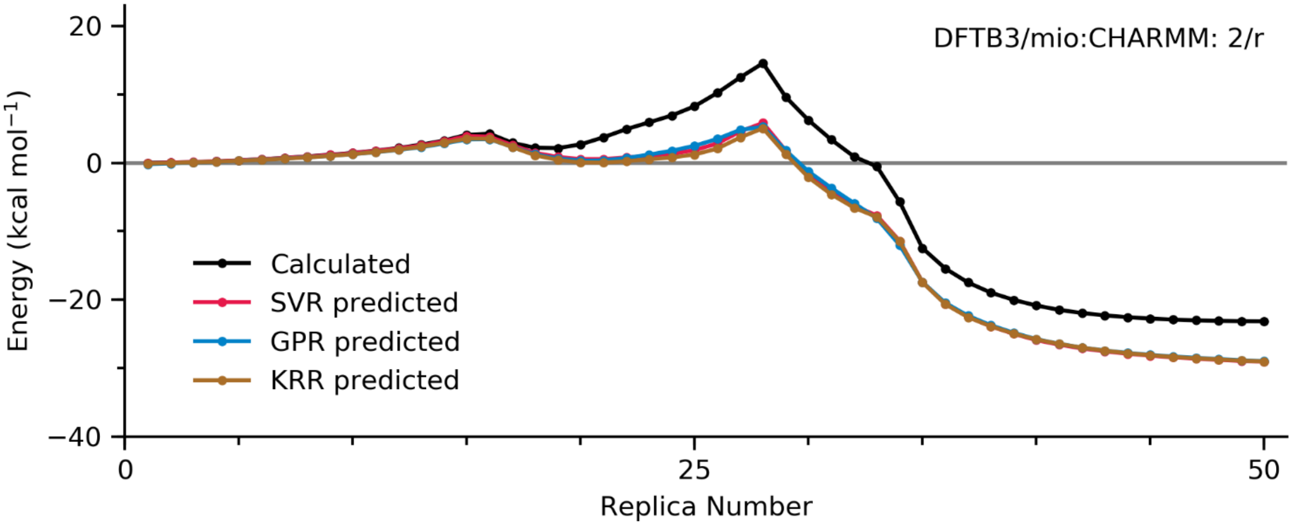

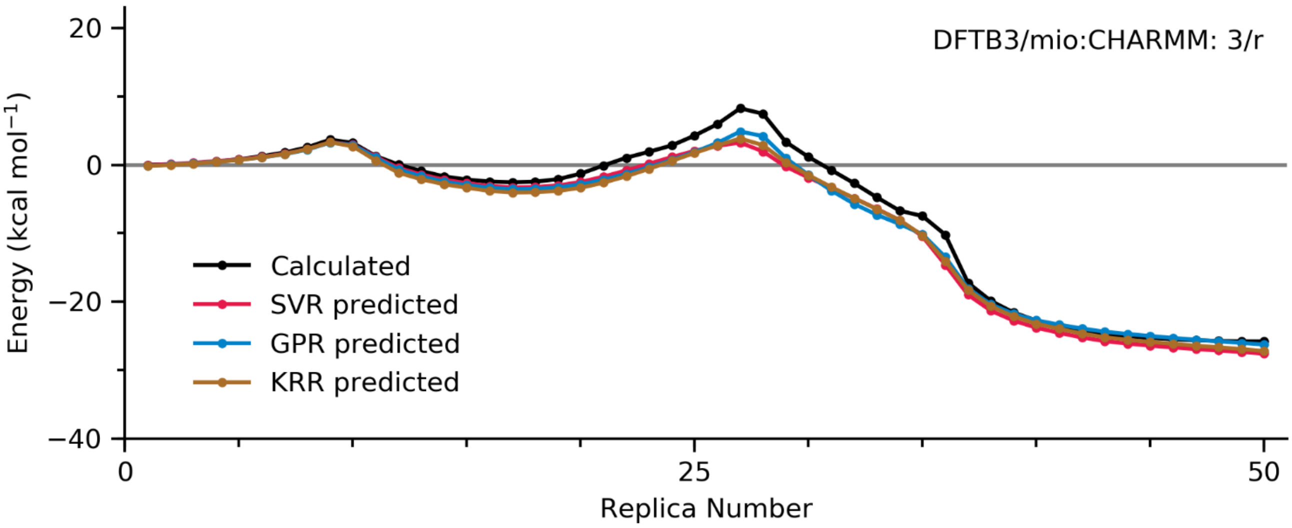
DFTB3/mio:CHARMM: 3/r
Energy (kcal mol^{-1})
Replica Number
20
0
−20
−40
0
25
50
Calculated
SVR predicted
GPR predicted
KRR predicted

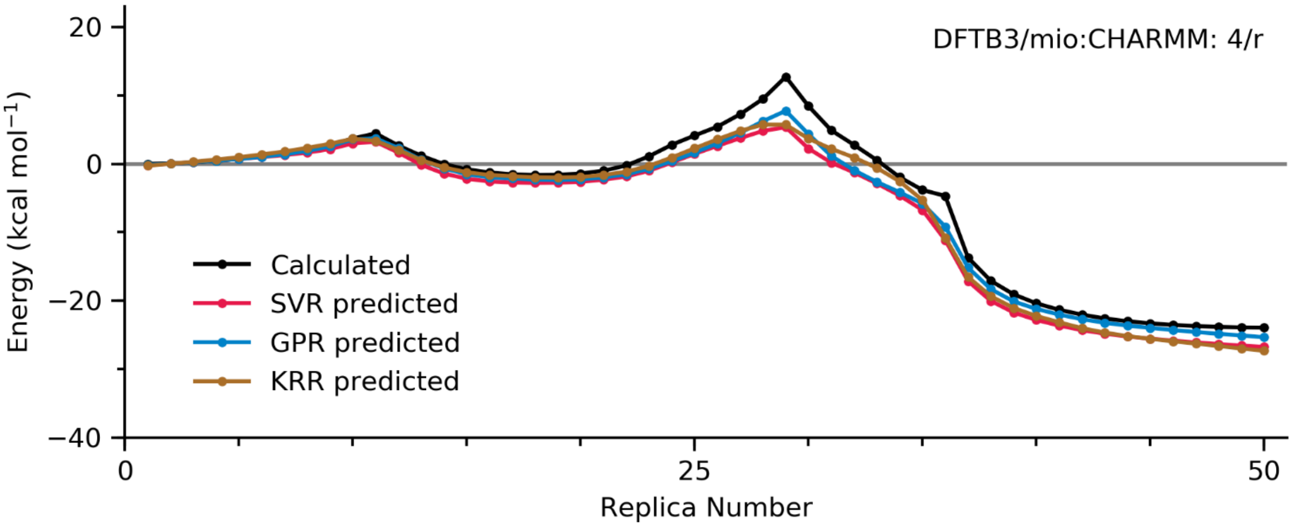
DFTB3/mio:CHARMM: 4/r
Energy (kcal mol^{-1})
Replica Number
20
0
−20
−40
0
25
50
Calculated
SVR predicted
GPR predicted
KRR predicted

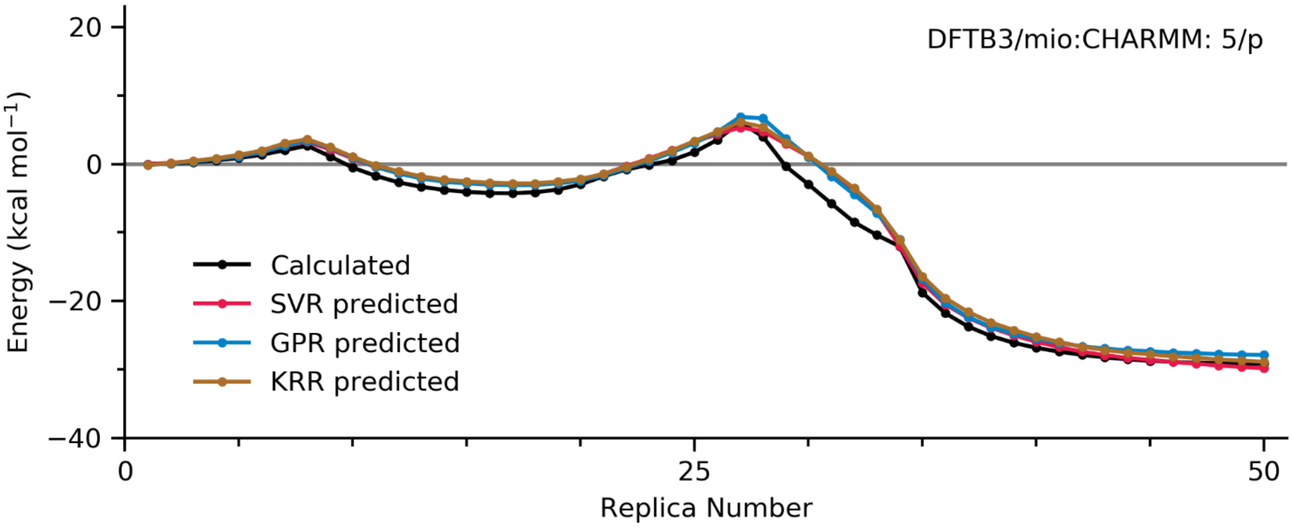
DFTB3/mio:CHARMM: 5/p
Energy (kcal mol^{-1})
Replica Number
20
0
−20
−40
0
25
50
Calculated
SVR predicted
GPR predicted
KRR predicted

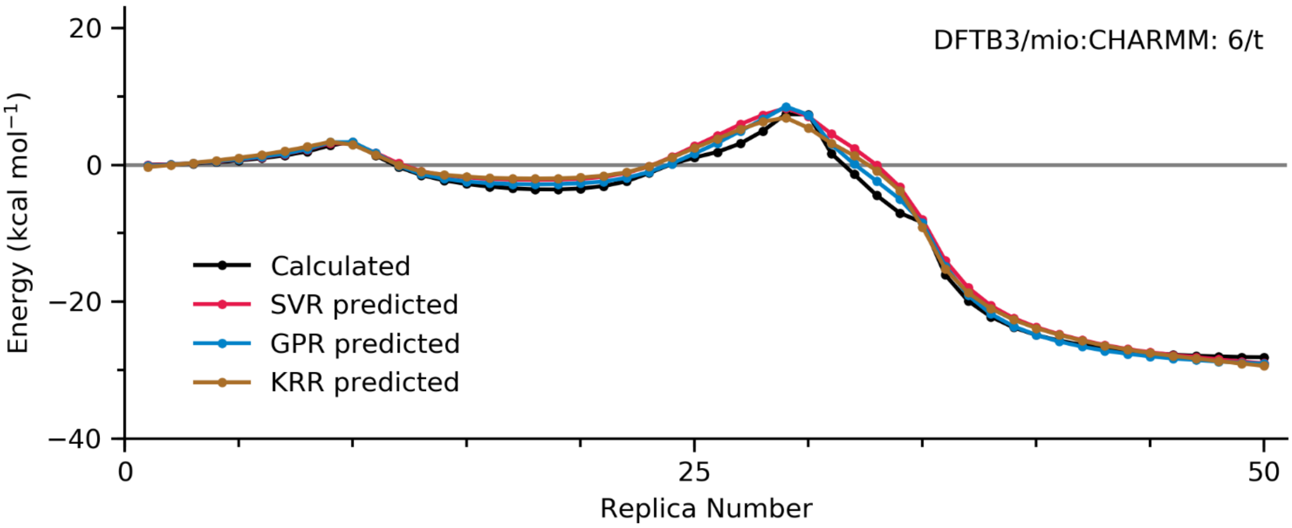
DFTB3/mio:CHARMM: 6/t
Energy (kcal mol^{-1})
Replica Number
Calculated
SVR predicted
GPR predicted
KRR predicted
20
0
−20
−40
0
25
50

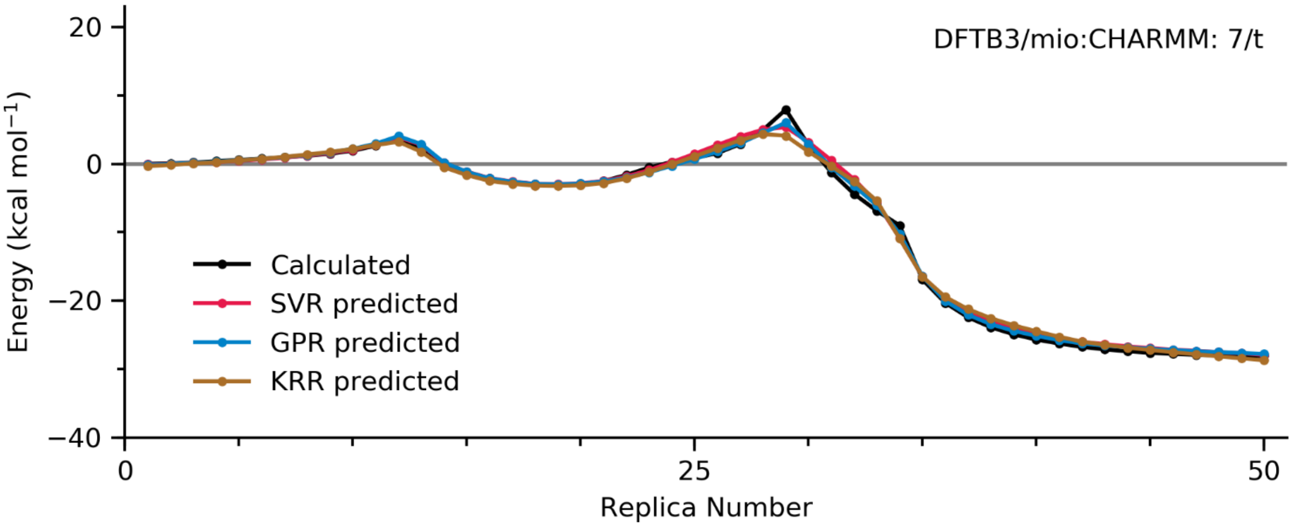
DFTB3/mio:CHARMM: 7/t
Energy (kcal mol^{-1})
Replica Number
Calculated
SVR predicted
GPR predicted
KRR predicted
20
0
−20
−40
0
25
50

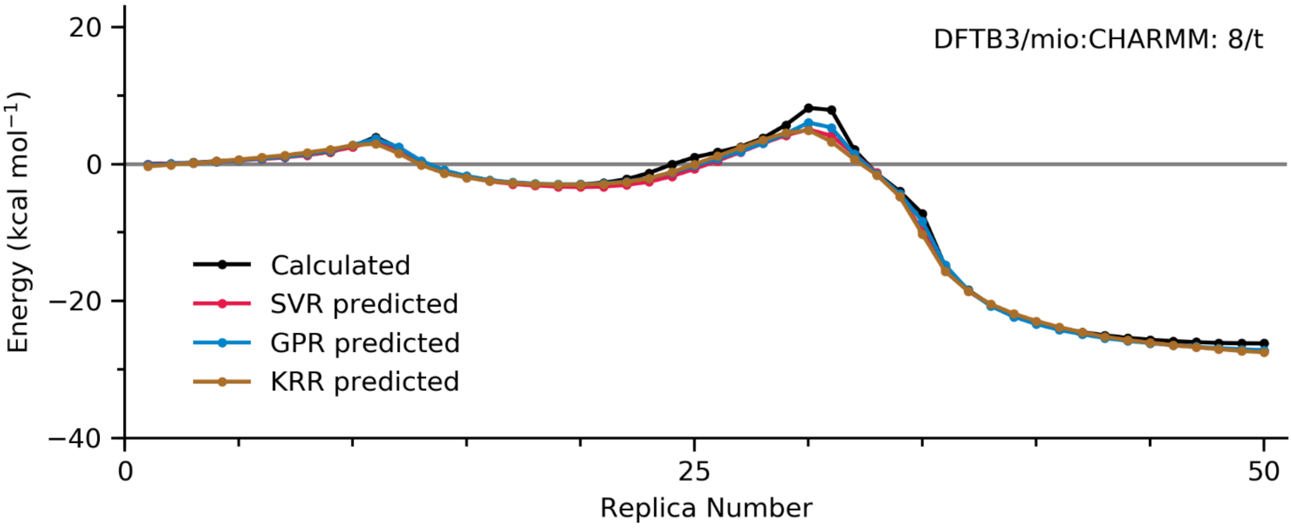
DFTB3/mio:CHARMM: 8/t
Energy (kcal mol^{-1})
Replica Number
Calculated
SVR predicted
GPR predicted
KRR predicted
20
0
−20
−40
0
25
50

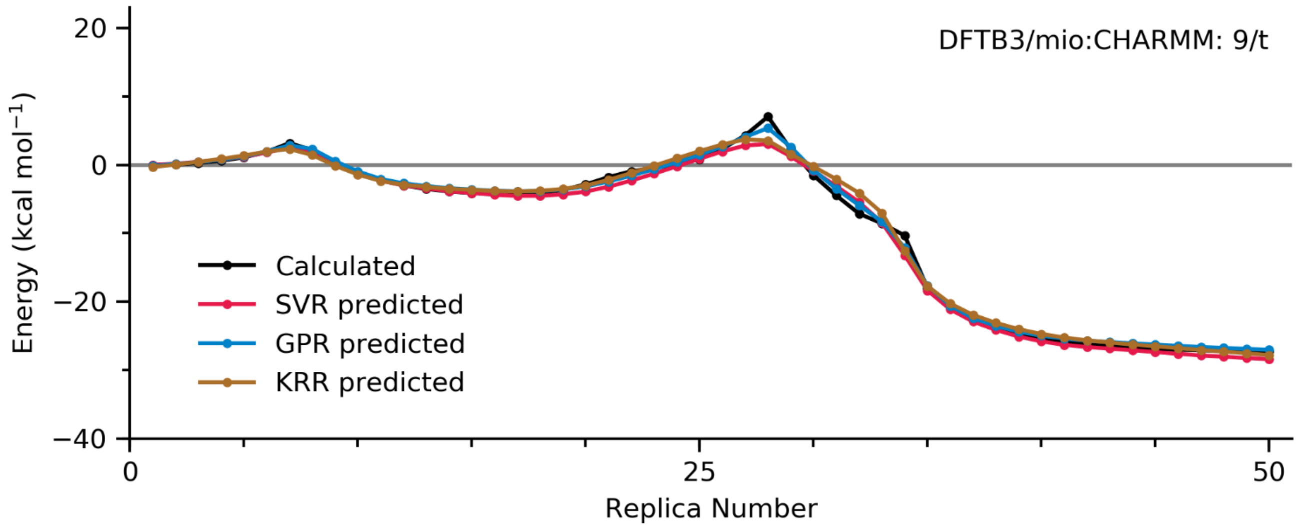
DFTB3/mio:CHARMM: 9/t
Energy (kcal mol^{-1})
Replica Number
Calculated
SVR predicted
GPR predicted
KRR predicted
20
0
−20
−40
0
25
50

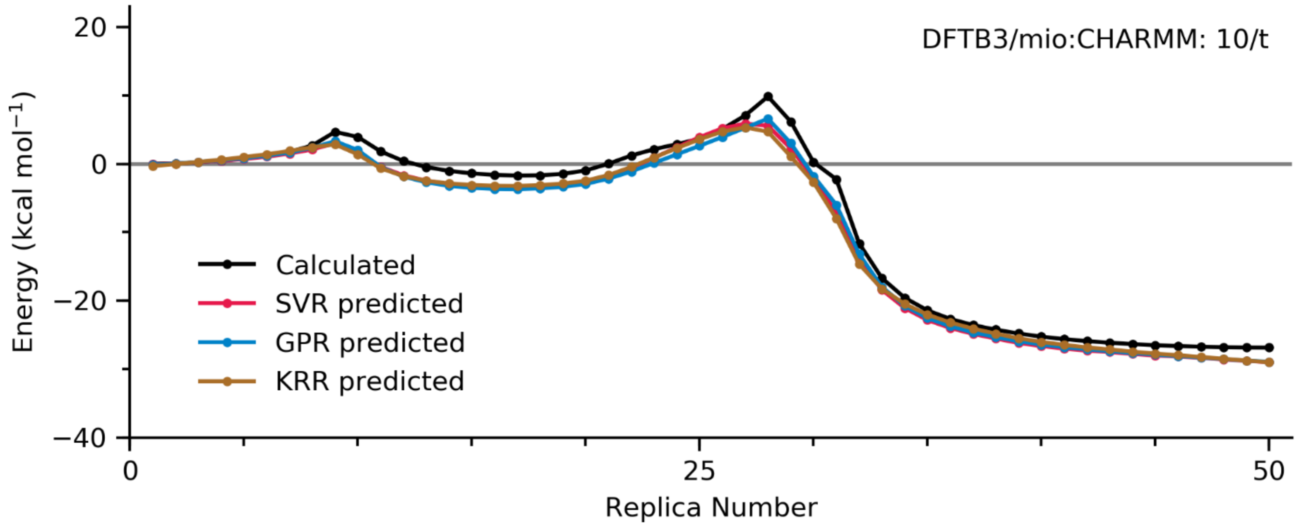
DFTB3/mio:CHARMM: 10/t
Energy (kcal mol^{-1})
Replica Number
Calculated
SVR predicted
GPR predicted
KRR predicted
20
0
−20
−40
0
25
50

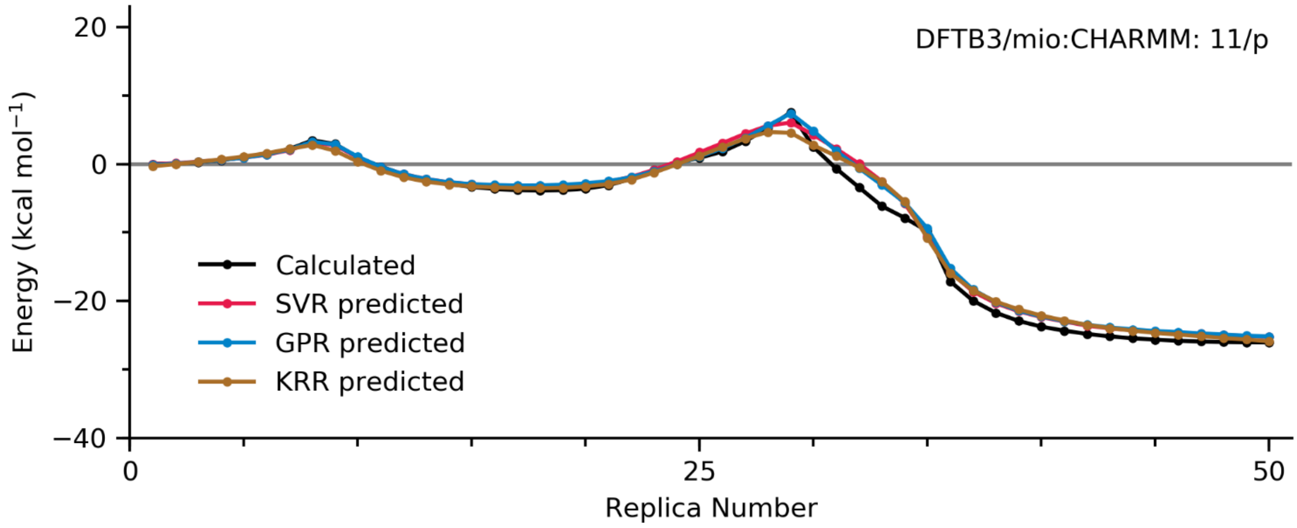
DFTB3/mio:CHARMM: 11/p
Energy (kcal mol^{-1})
Replica Number
Calculated
SVR predicted
GPR predicted
KRR predicted
20
0
−20
−40
0
25
50

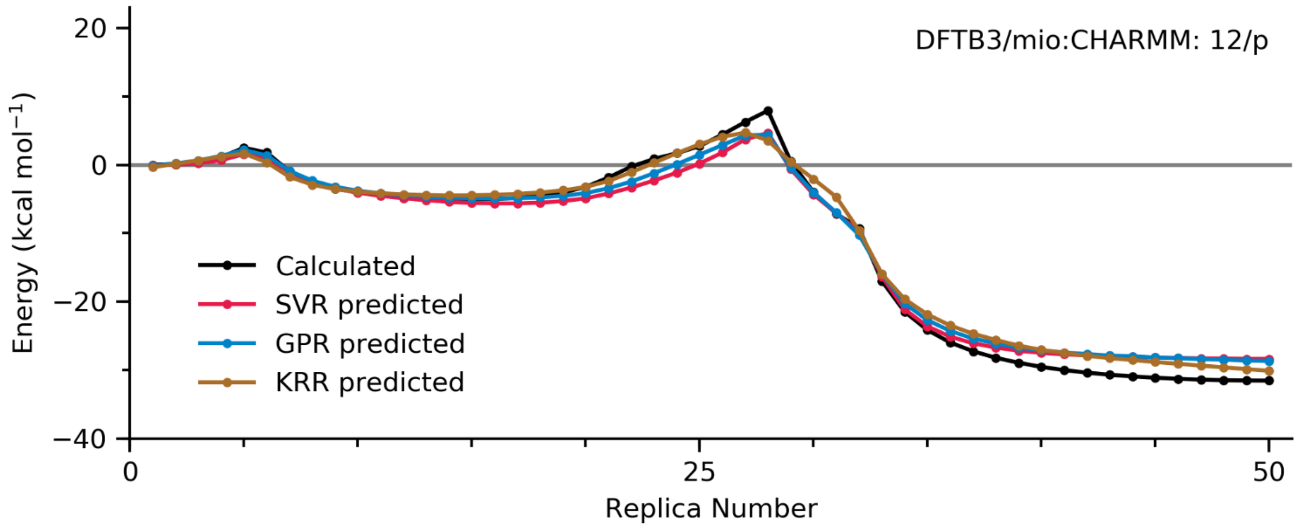
DFTB3/mio:CHARMM: 12/p
Energy (kcal mol^{-1})
Replica Number
Calculated
SVR predicted
GPR predicted
KRR predicted
20
0
−20
−40
0
25
50

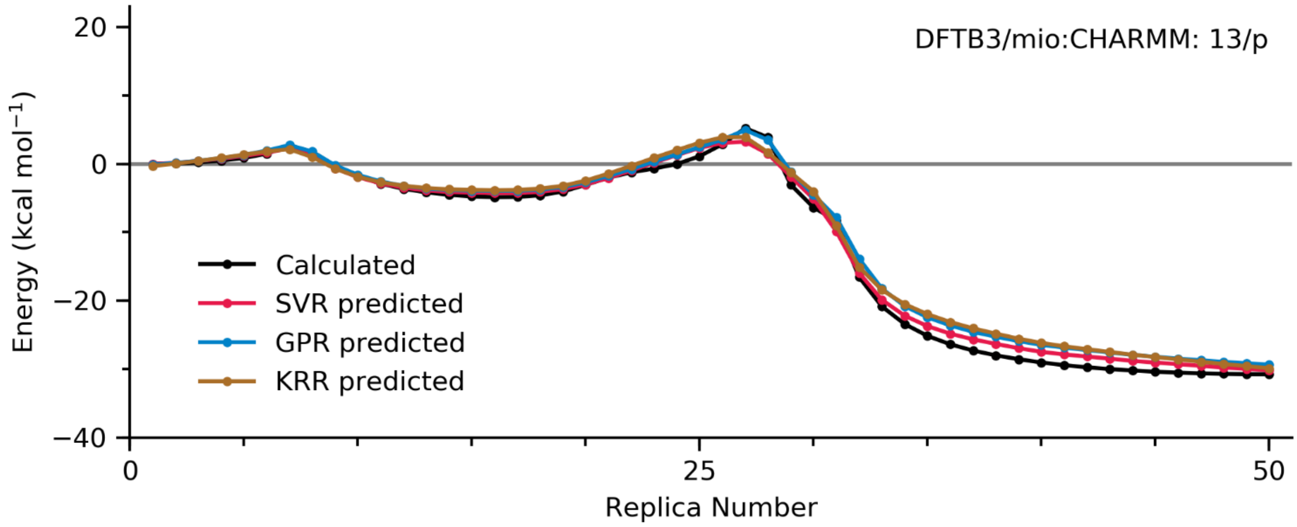
DFTB3/mio:CHARMM: 13/p
Energy (kcal mol^{-1})
Replica Number
Calculated
SVR predicted
GPR predicted
KRR predicted
20
0
−20
−40
0
25
50

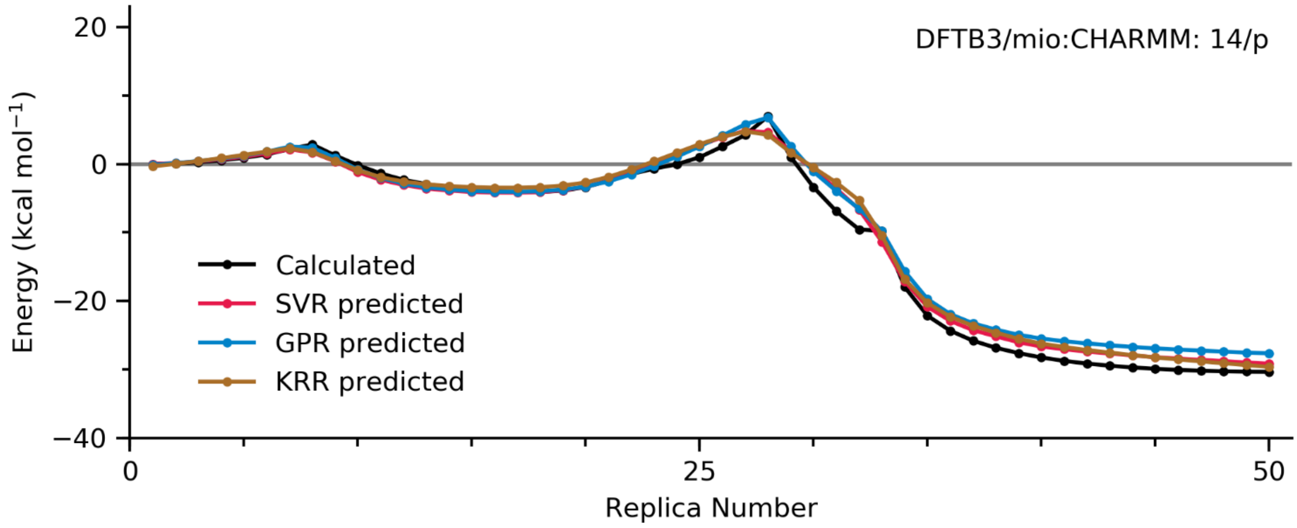
DFTB3/mio:CHARMM: 14/p
Energy (kcal mol^{-1})
Replica Number
Calculated
SVR predicted
GPR predicted
KRR predicted
20
0
−20
−40
0
25
50

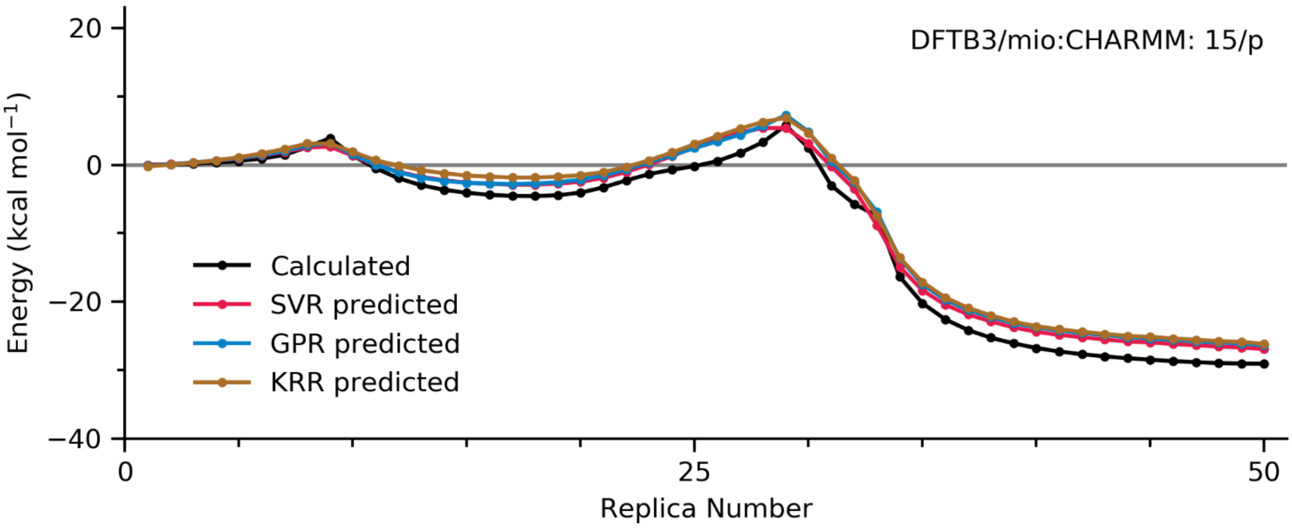
DFTB3/mio:CHARMM: 15/p
Calculated
SVR predicted
GPR predicted
KRR predicted
Energy (kcal mol^{-1})
Replica Number

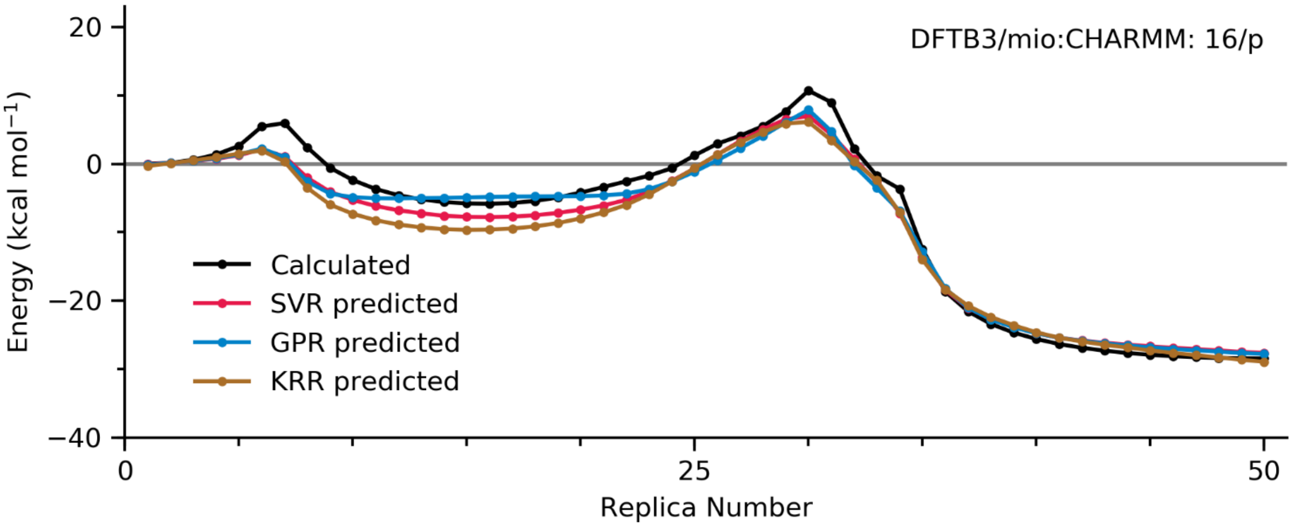
DFTB3/mio:CHARMM: 16/p
Calculated
SVR predicted
GPR predicted
KRR predicted
Energy (kcal mol^{-1})
Replica Number

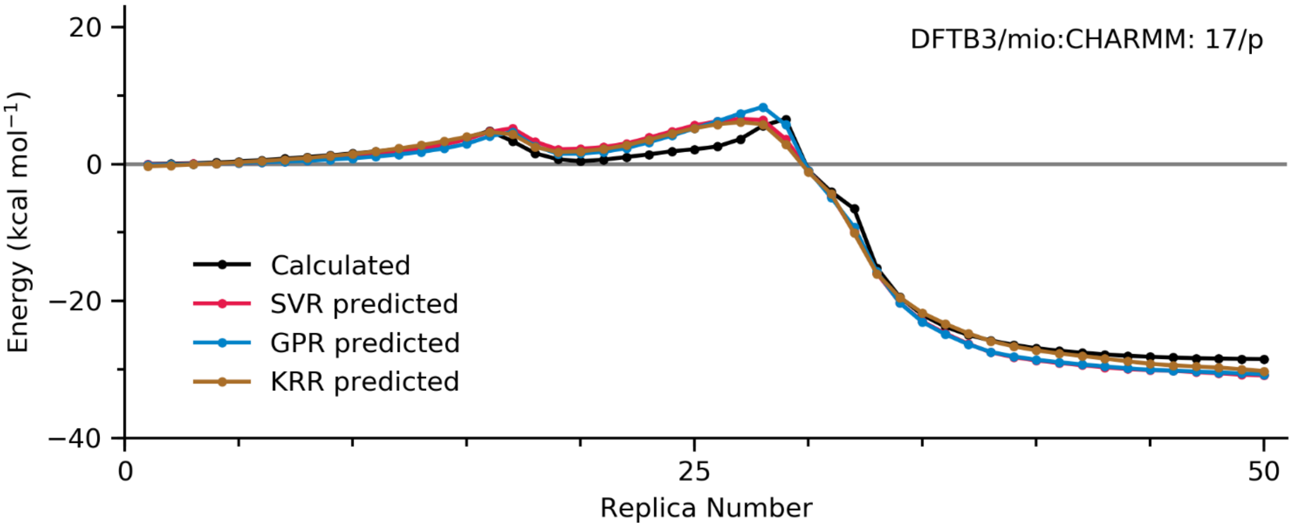
DFTB3/mio:CHARMM: 17/p
Calculated
SVR predicted
GPR predicted
KRR predicted
Energy (kcal mol^{-1})
Replica Number

Supplementary Figure 35 to 52. B3LYP/6-31G:CHARMM pathways

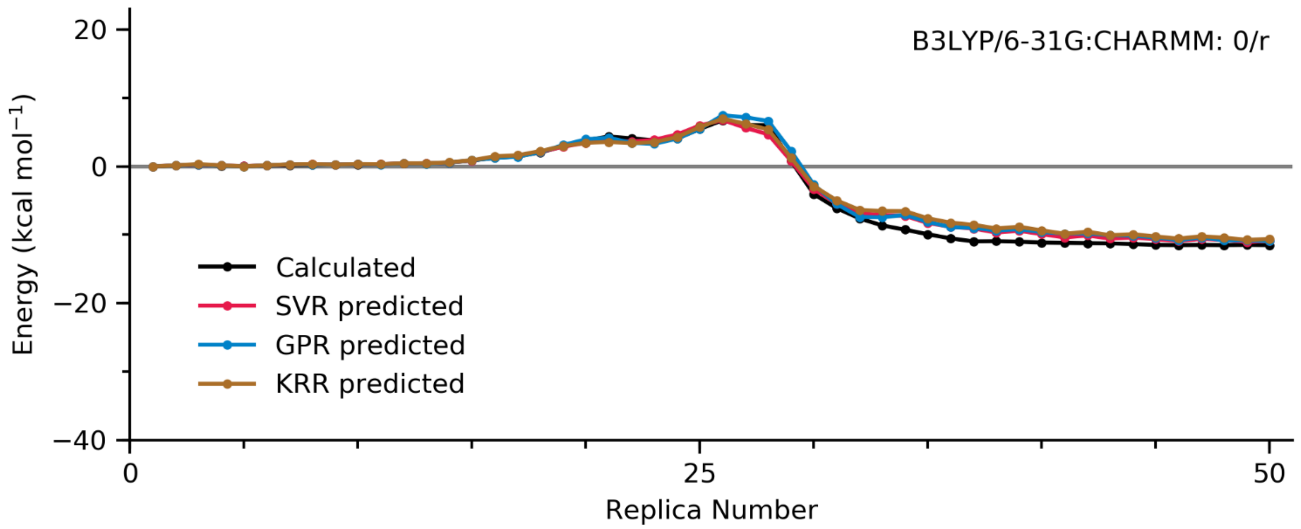

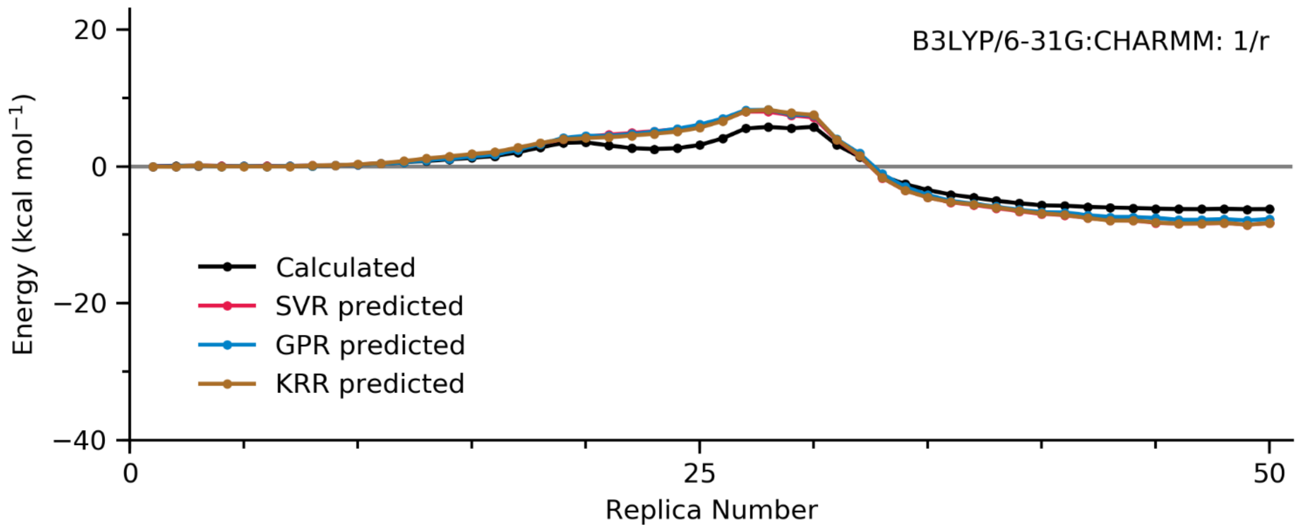

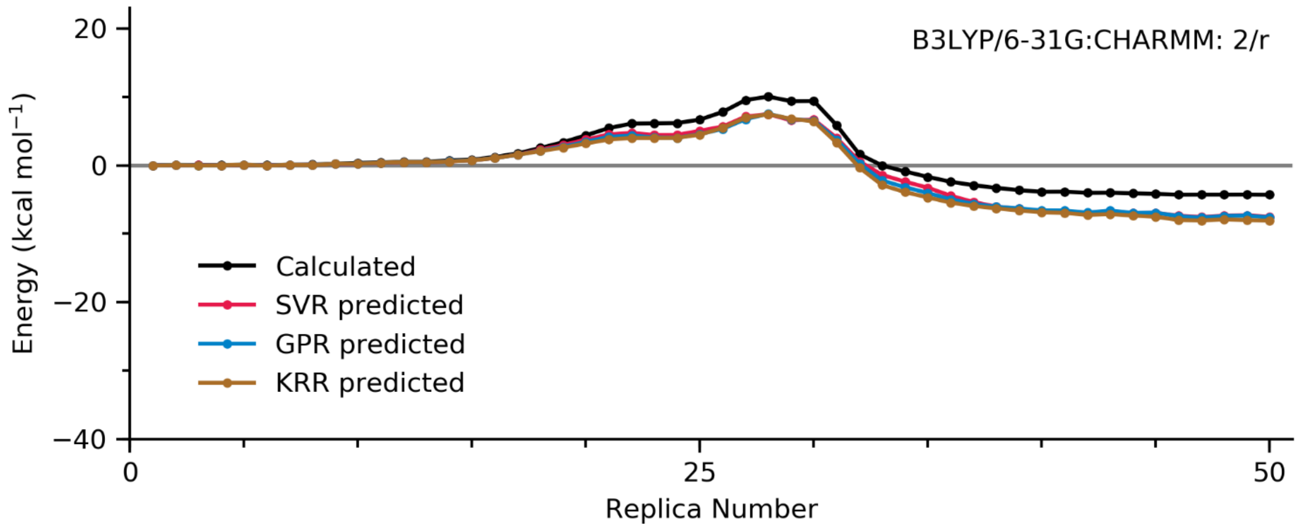

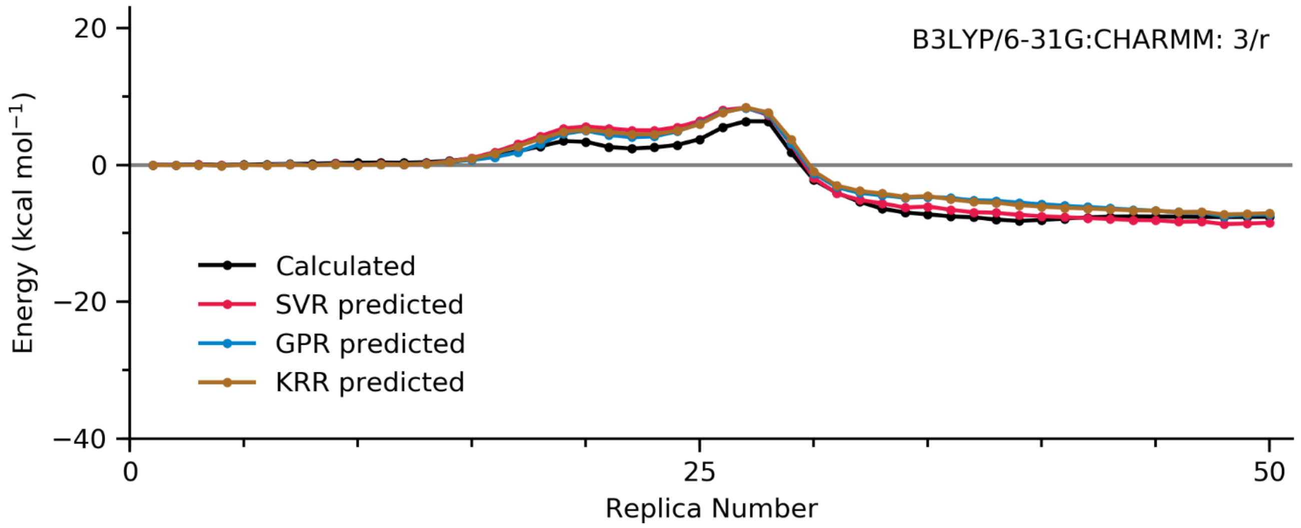
B3LYP/6-31G:CHARMM: 3/r
Calculated
SVR predicted
GPR predicted
KRR predicted
Energy (kcal mol⁻¹)
Replica Number

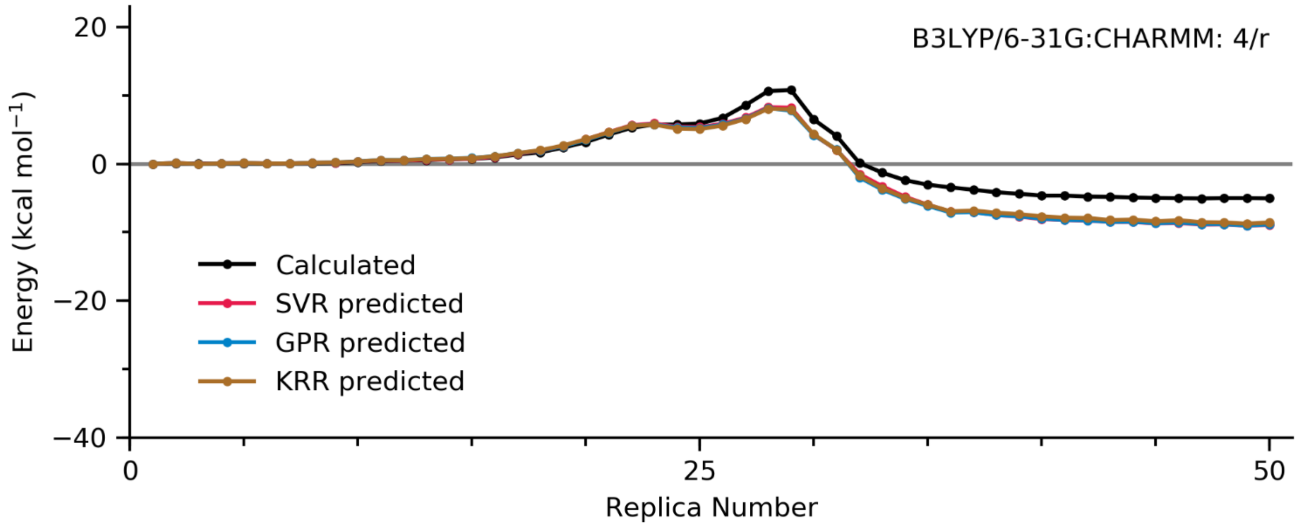
B3LYP/6-31G:CHARMM: 4/r
Calculated
SVR predicted
GPR predicted
KRR predicted
Energy (kcal mol⁻¹)
Replica Number

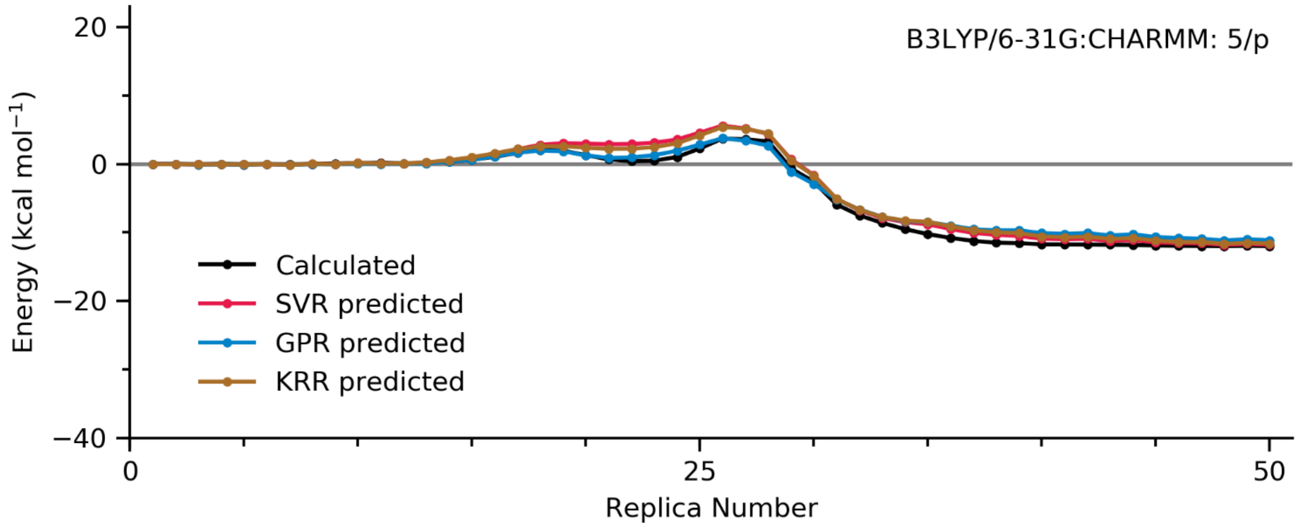
B3LYP/6-31G:CHARMM: 5/p
Calculated
SVR predicted
GPR predicted
KRR predicted
Energy (kcal mol⁻¹)
Replica Number

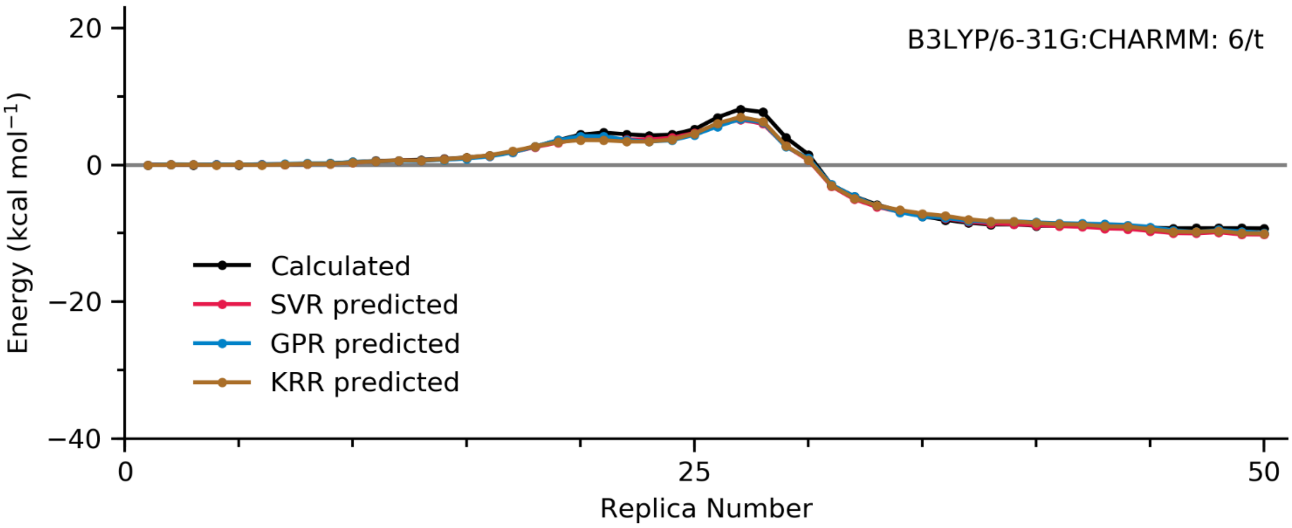
B3LYP/6-31G:CHARMM: 6/t
Energy (kcal mol^{-1})
Replica Number
Calculated
SVR predicted
GPR predicted
KRR predicted
20
0
−20
−40
0
25
50

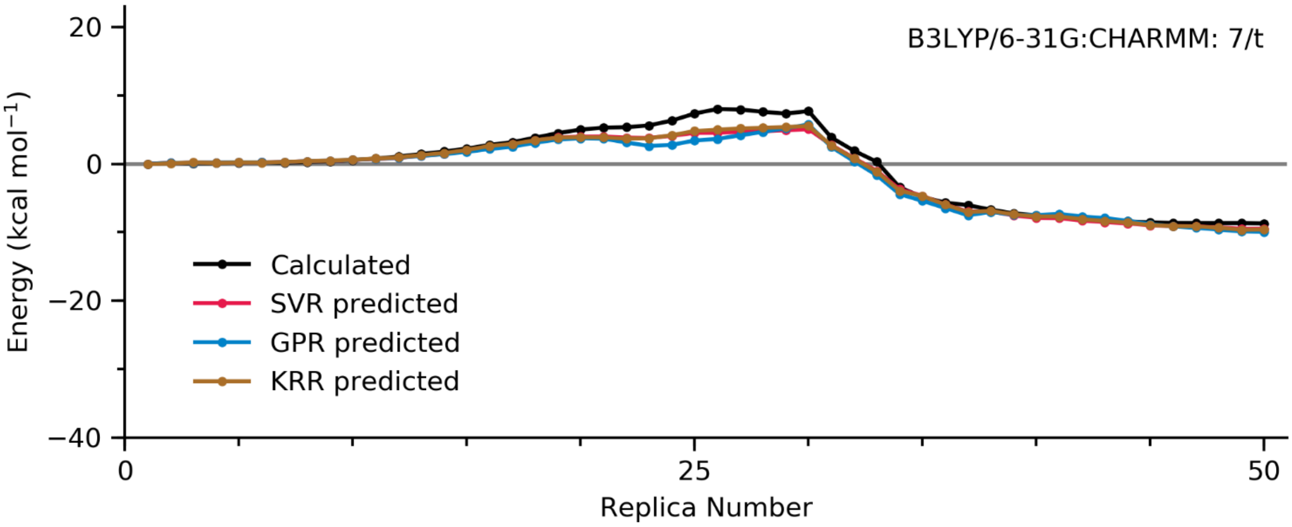
B3LYP/6-31G:CHARMM: 7/t
Energy (kcal mol^{-1})
Replica Number
Calculated
SVR predicted
GPR predicted
KRR predicted
20
0
−20
−40
0
25
50

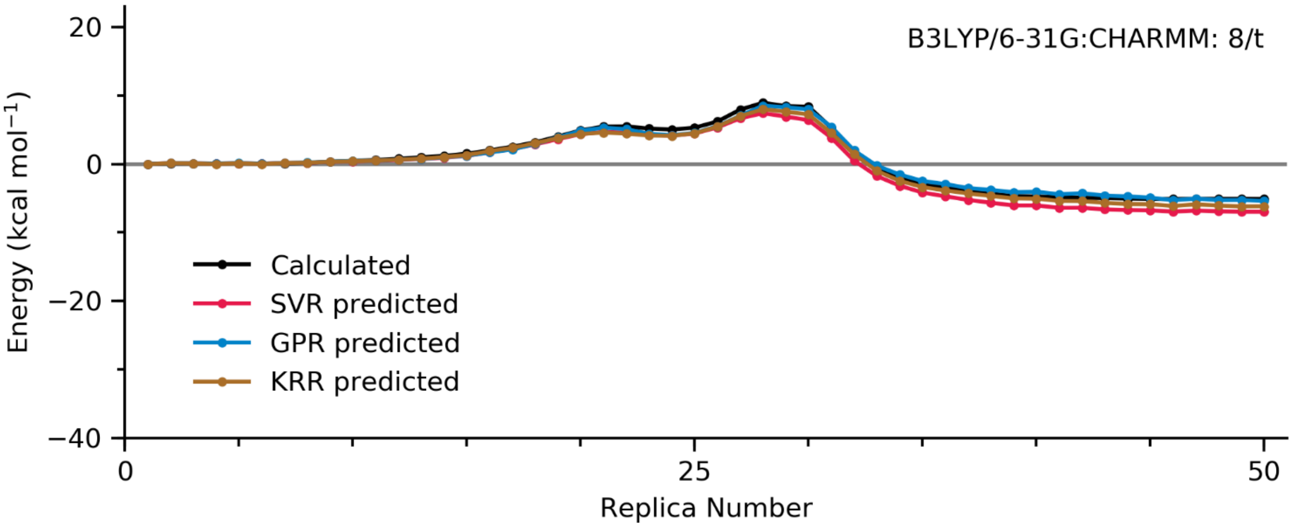
B3LYP/6-31G:CHARMM: 8/t
Energy (kcal mol^{-1})
Replica Number
Calculated
SVR predicted
GPR predicted
KRR predicted
20
0
−20
−40
0
25
50

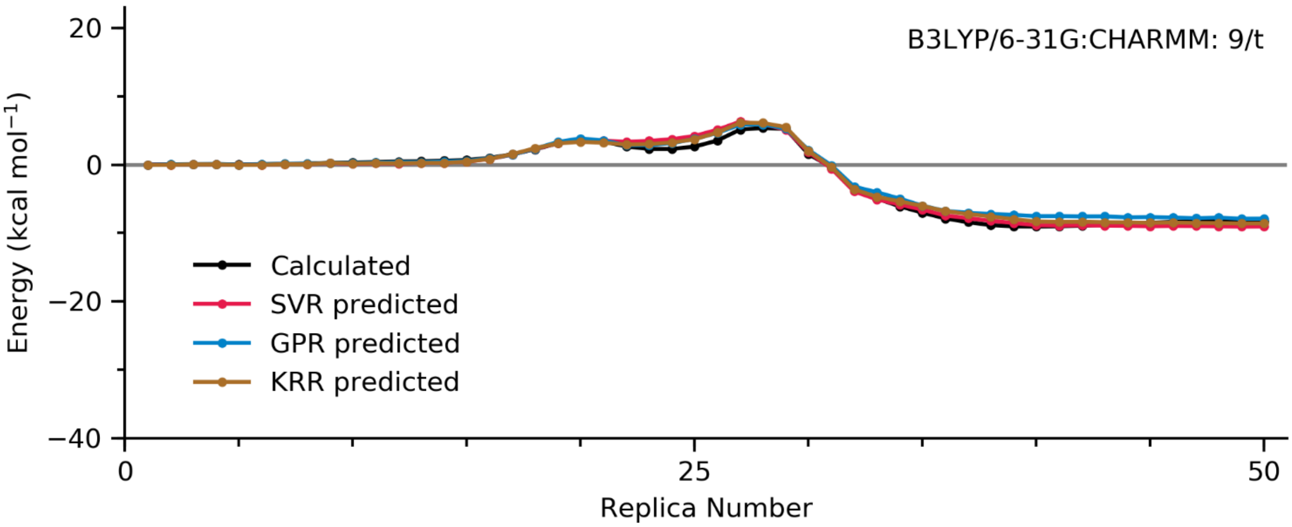
B3LYP/6-31G:CHARMM: 9/t
Energy (kcal mol−1)
20
0
−20
−40
0
25
50
Replica Number
Calculated
SVR predicted
GPR predicted
KRR predicted

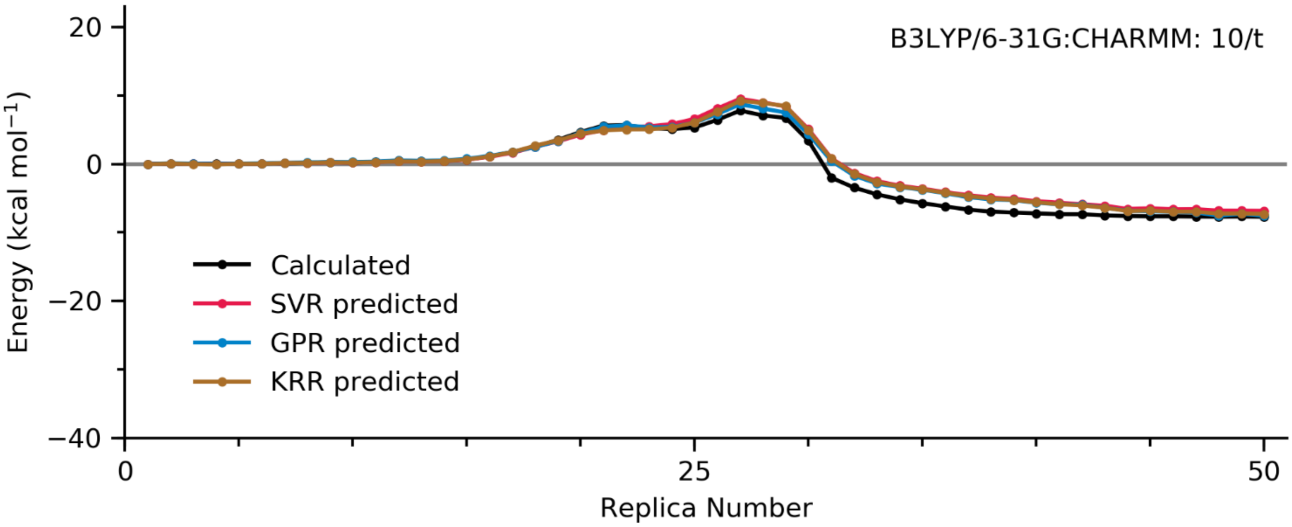
B3LYP/6-31G:CHARMM: 10/t
Energy (kcal mol−1)
20
0
−20
−40
0
25
50
Replica Number
Calculated
SVR predicted
GPR predicted
KRR predicted

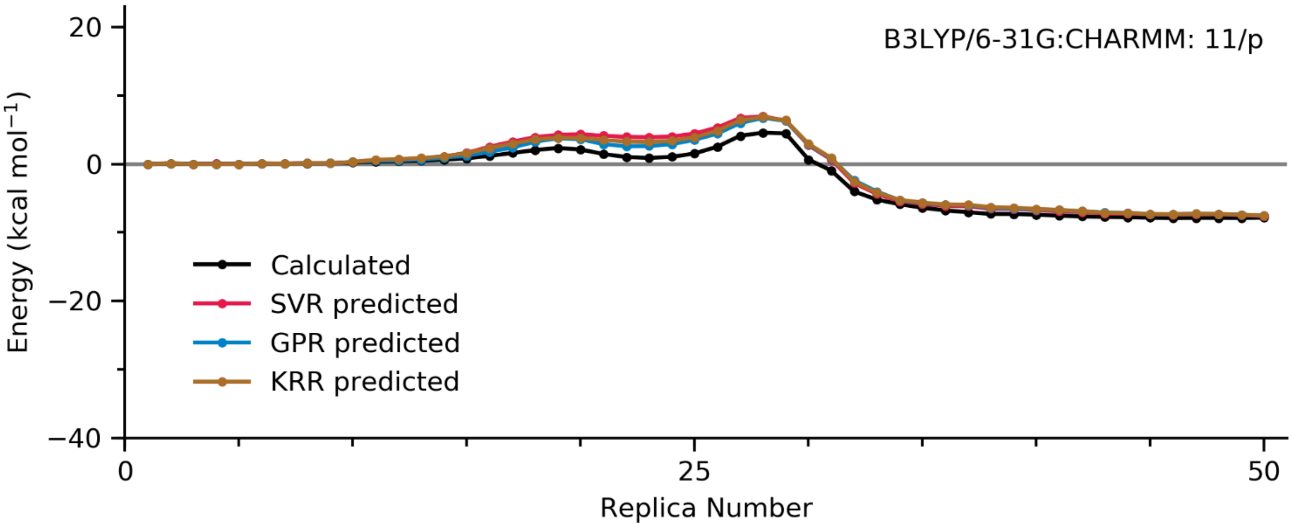
B3LYP/6-31G:CHARMM: 11/p
Energy (kcal mol−1)
20
0
−20
−40
0
25
50
Replica Number
Calculated
SVR predicted
GPR predicted
KRR predicted

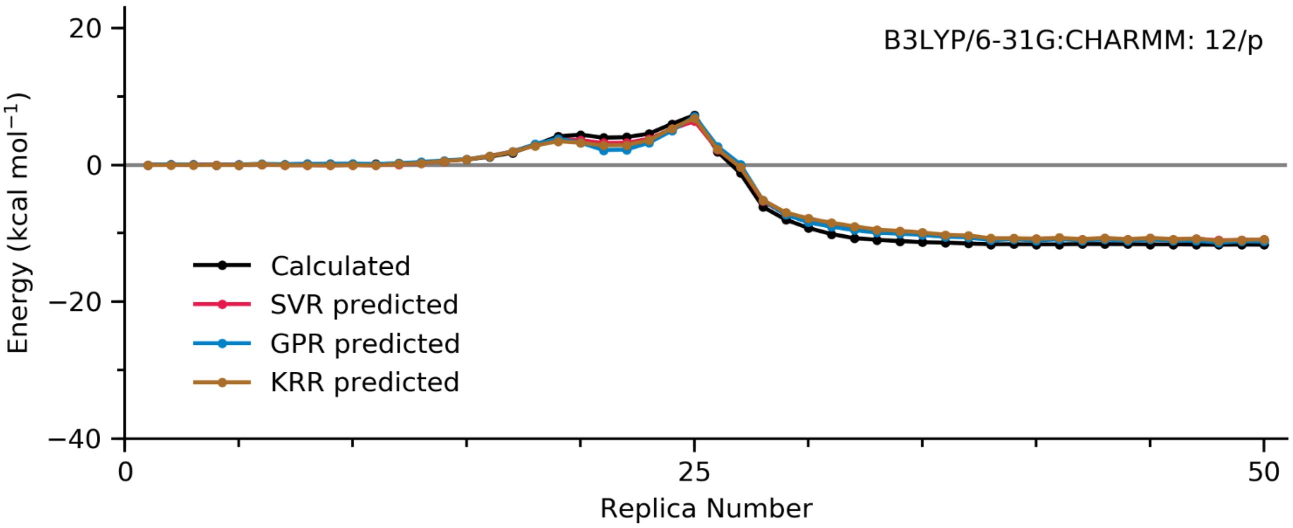
B3LYP/6-31G:CHARMM: 12/p
Energy (kcal mol−1)
Replica Number
Calculated
SVR predicted
GPR predicted
KRR predicted
20
0
−20
−40
0
25
50

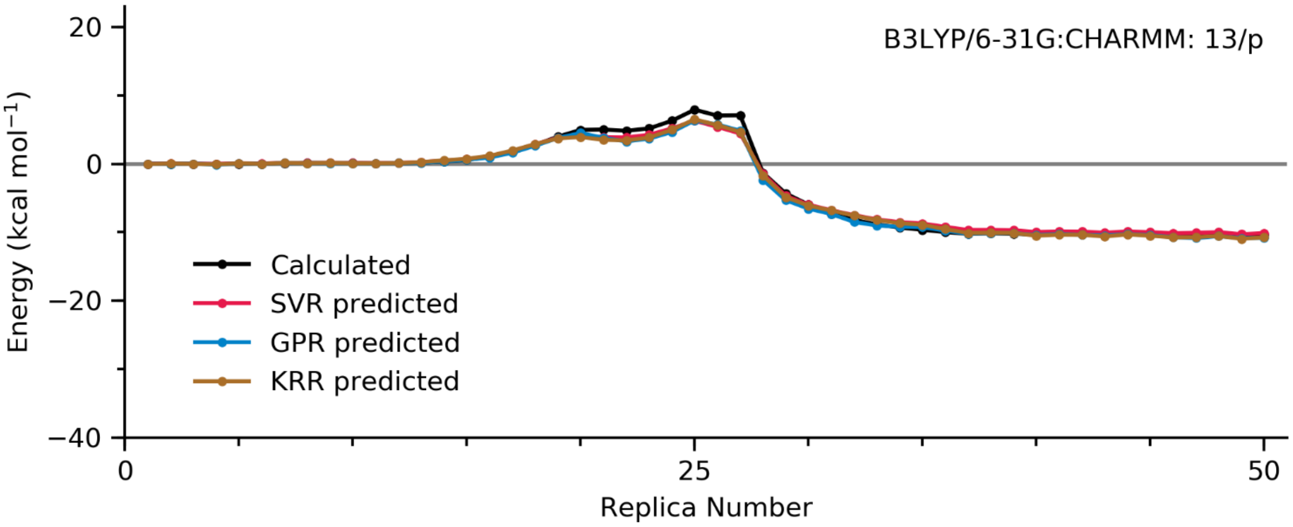
B3LYP/6-31G:CHARMM: 13/p
Energy (kcal mol−1)
Replica Number
Calculated
SVR predicted
GPR predicted
KRR predicted
20
0
−20
−40
0
25
50

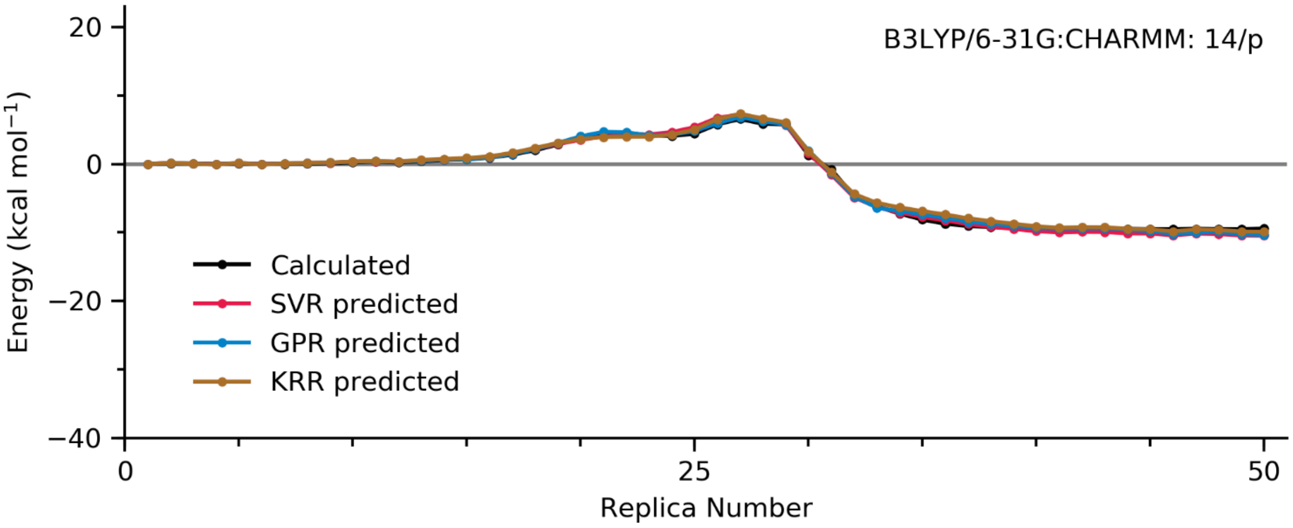
B3LYP/6-31G:CHARMM: 14/p
Energy (kcal mol−1)
Replica Number
Calculated
SVR predicted
GPR predicted
KRR predicted
20
0
−20
−40
0
25
50

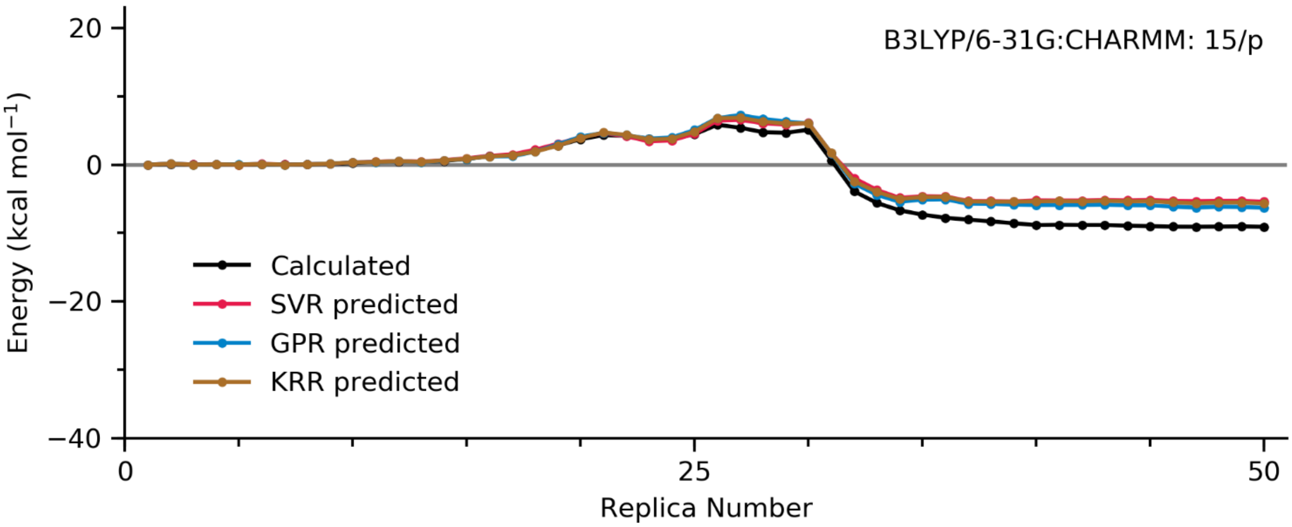
B3LYP/6-31G:CHARMM: 15/p
Calculated
SVR predicted
GPR predicted
KRR predicted
Energy (kcal mol^{-1})
Replica Number
20
0
−20
−40
0
25
50

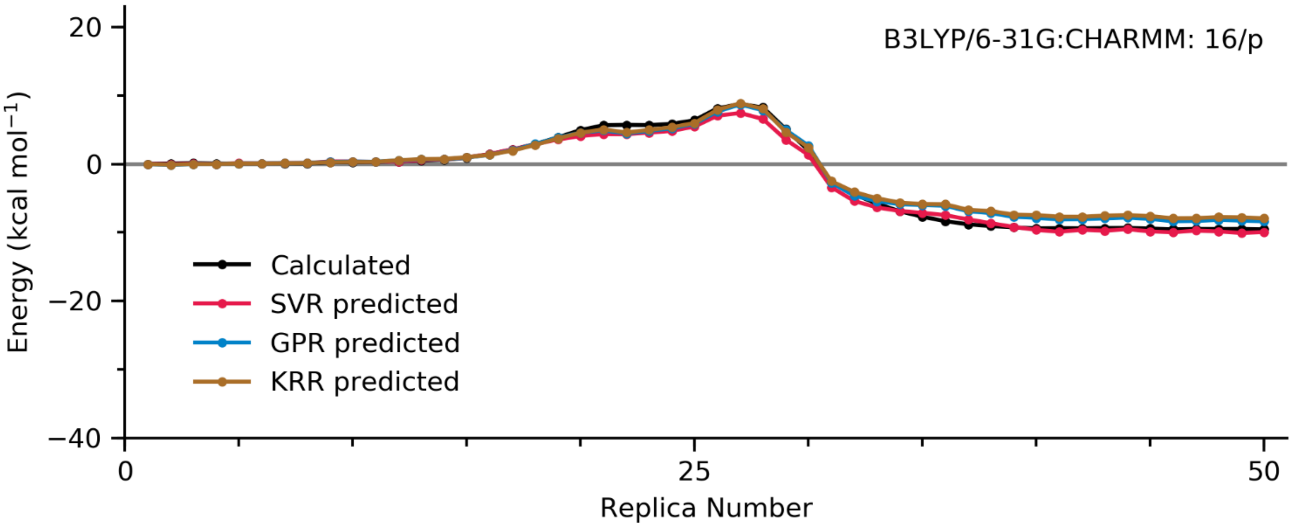
B3LYP/6-31G:CHARMM: 16/p
Calculated
SVR predicted
GPR predicted
KRR predicted
Energy (kcal mol^{-1})
Replica Number
20
0
−20
−40
0
25
50

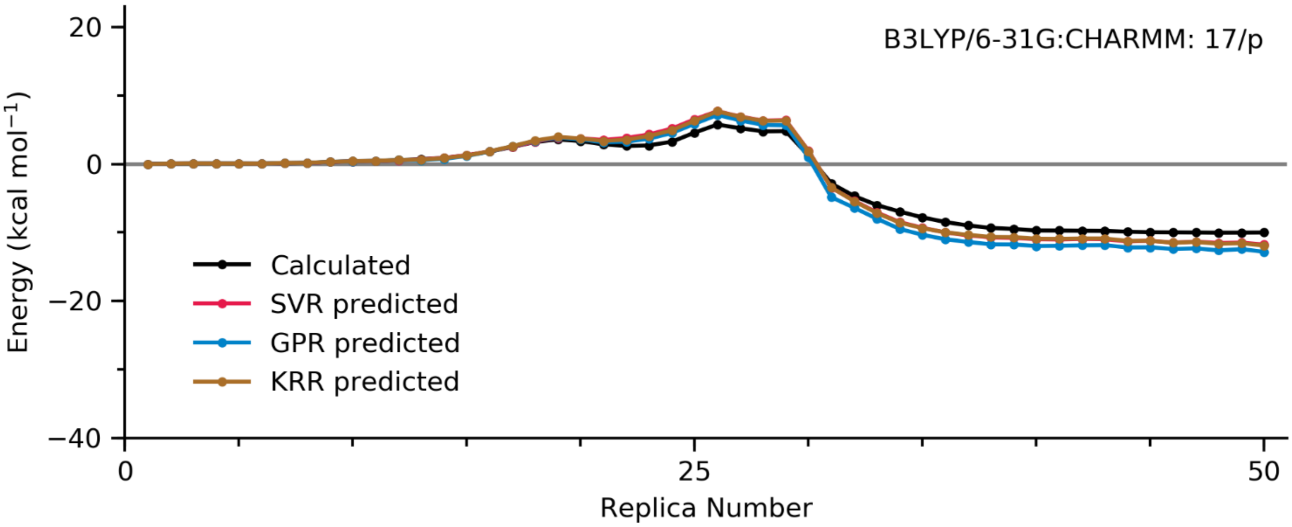
B3LYP/6-31G:CHARMM: 17/p
Calculated
SVR predicted
GPR predicted
KRR predicted
Energy (kcal mol^{-1})
Replica Number
20
0
−20
−40
0
25
50

Supplementary Figure 53 to 70. B3LYP/6-31+G*:CHARMM pathways

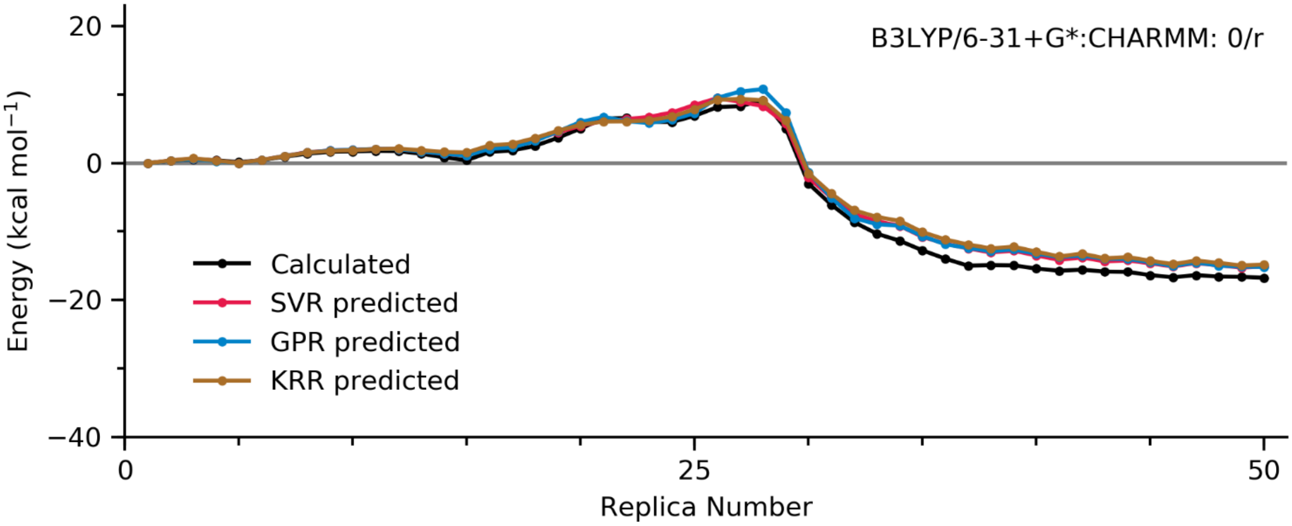

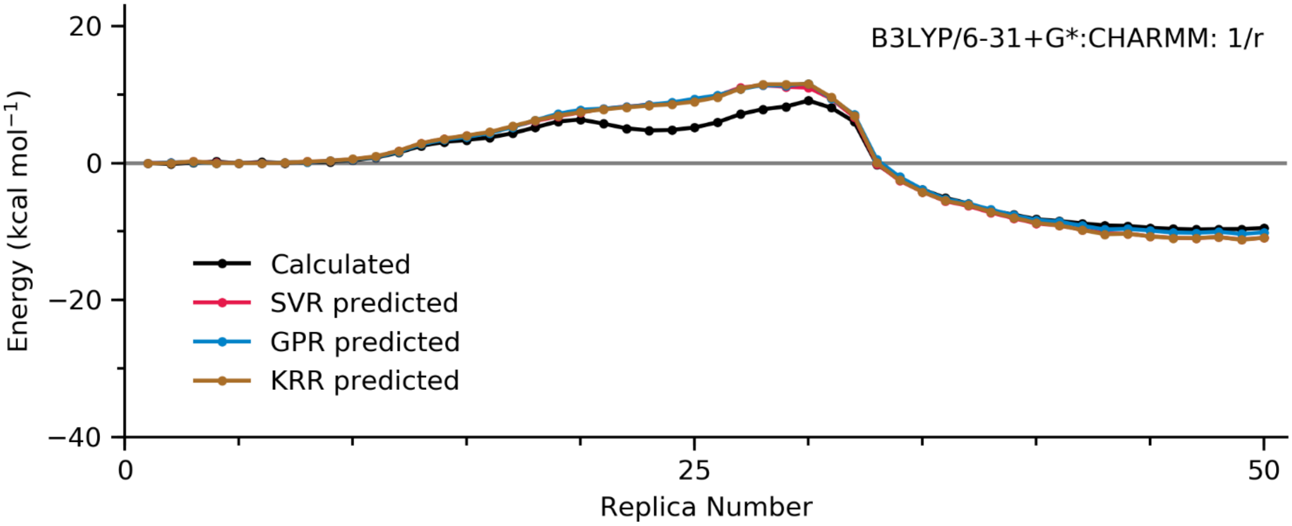

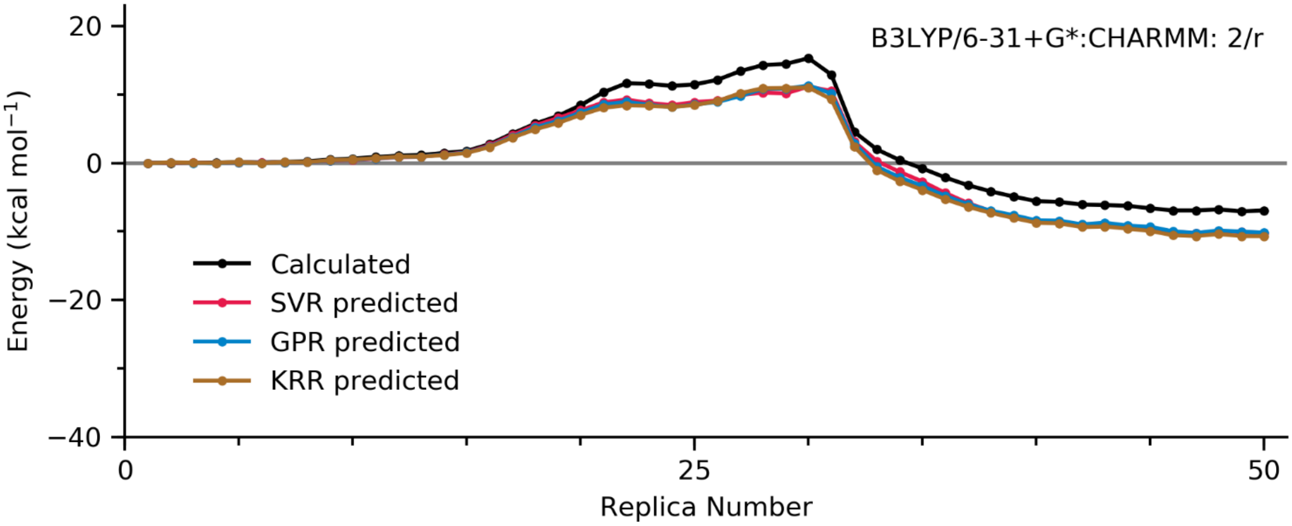

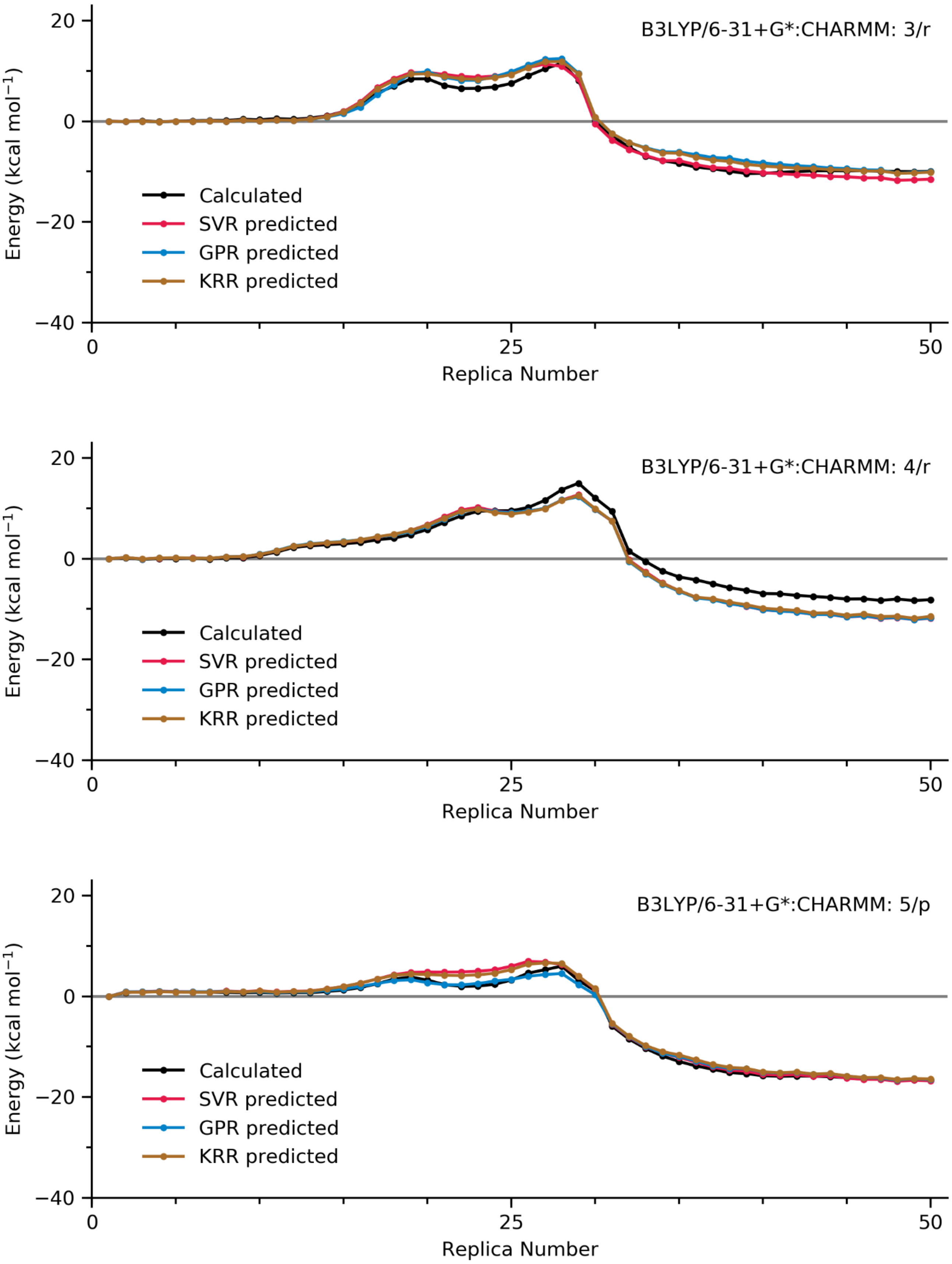
B3LYP/6-31+G*:CHARMM: 3/r
B3LYP/6-31+G*:CHARMM: 4/r
B3LYP/6-31+G*:CHARMM: 5/p
Energy (kcal mol−1)
Replica Number
Calculated
SVR predicted
GPR predicted
KRR predicted

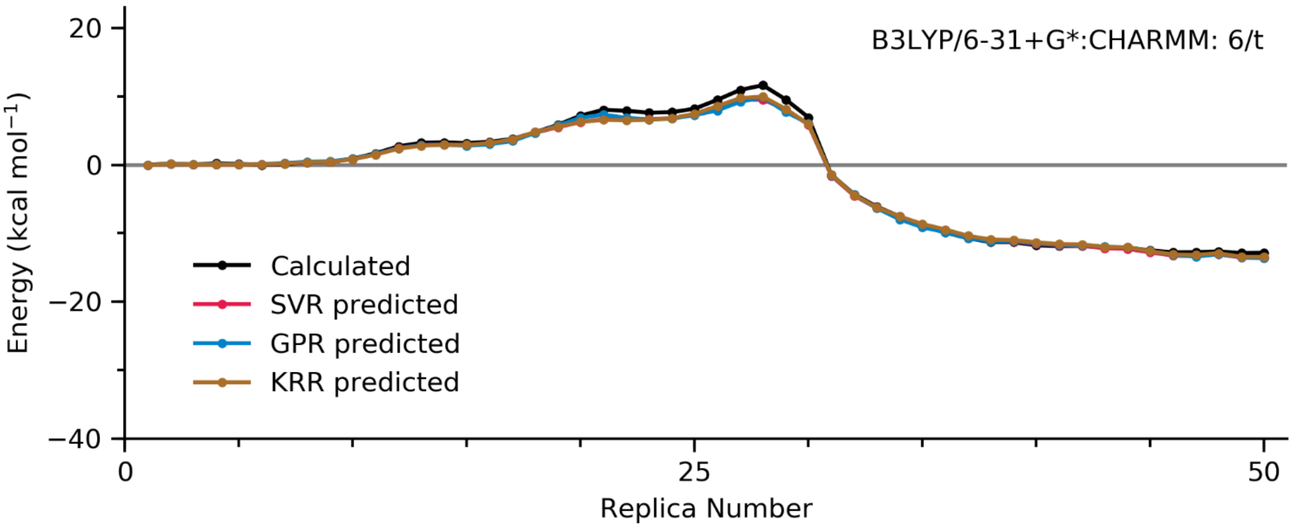

B3LYP/6-31+G*:CHARMM: 6/t
Energy (kcal mol^{-1})
Replica Number
Calculated
SVR predicted
GPR predicted
KRR predicted

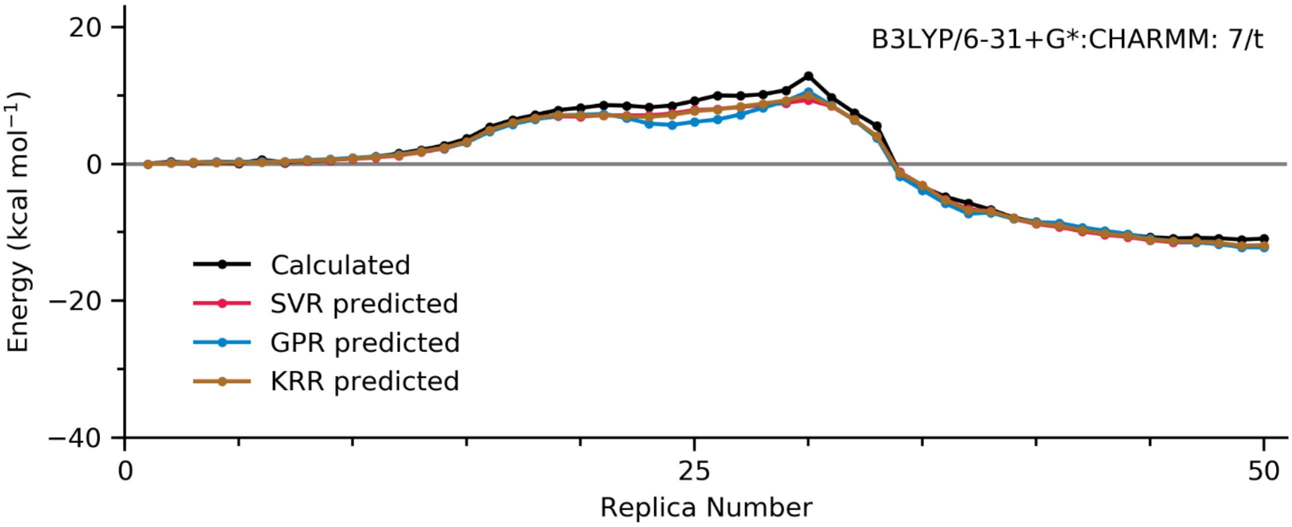

B3LYP/6-31+G*:CHARMM: 7/t
Energy (kcal mol^{-1})
Replica Number
Calculated
SVR predicted
GPR predicted
KRR predicted

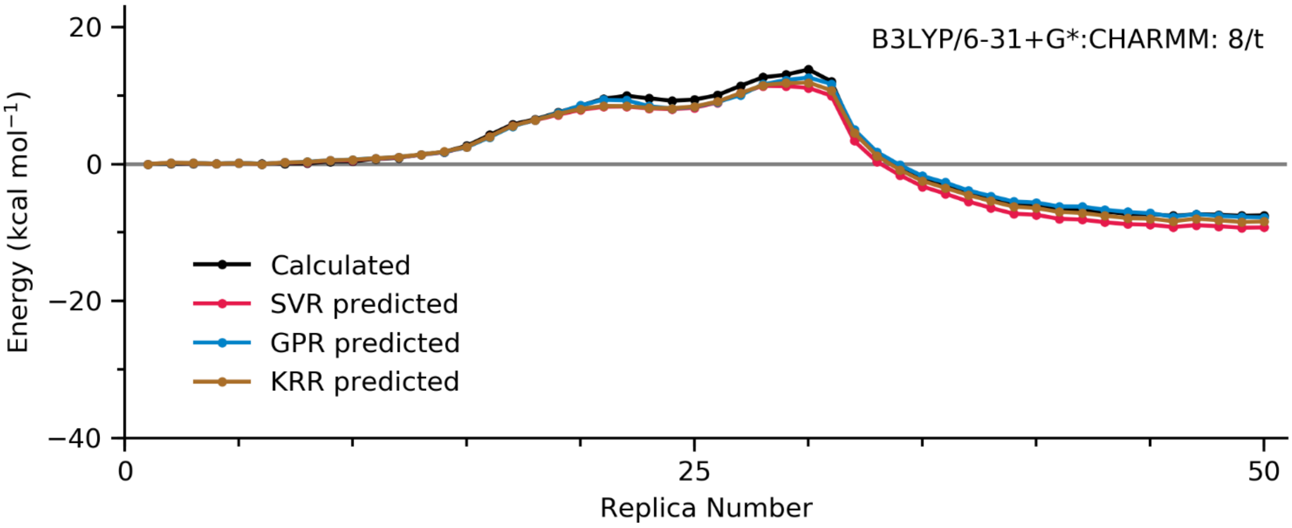

B3LYP/6-31+G*:CHARMM: 8/t
Energy (kcal mol^{-1})
Replica Number
Calculated
SVR predicted
GPR predicted
KRR predicted

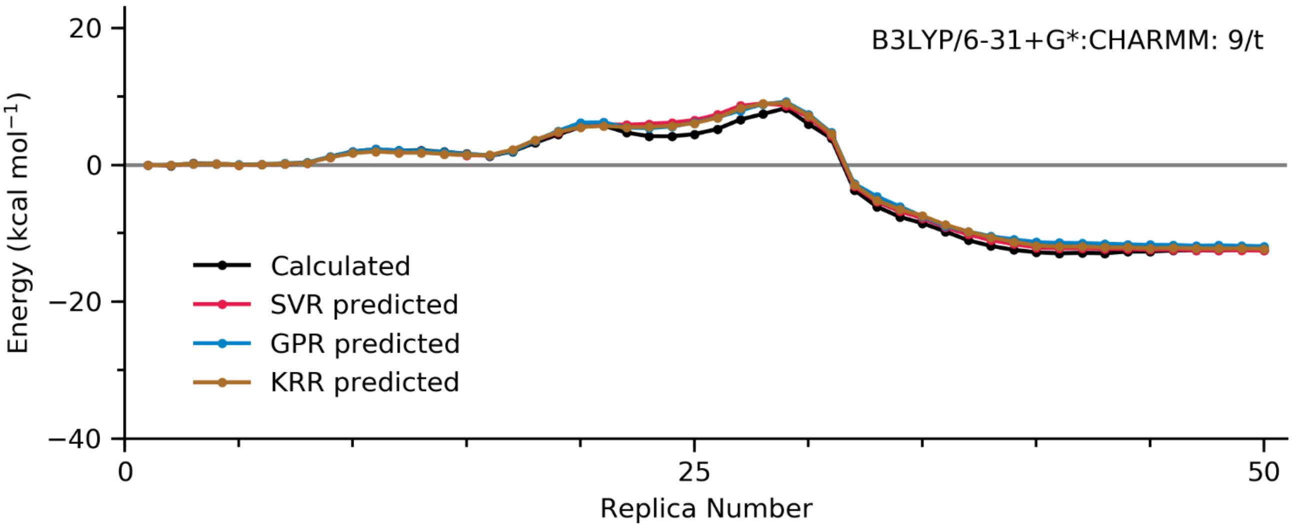
B3LYP/6-31+G*:CHARMM: 9/t
Energy (kcal mol^{-1})
Replica Number
Calculated
SVR predicted
GPR predicted
KRR predicted
20
0
−20
−40
0
25
50

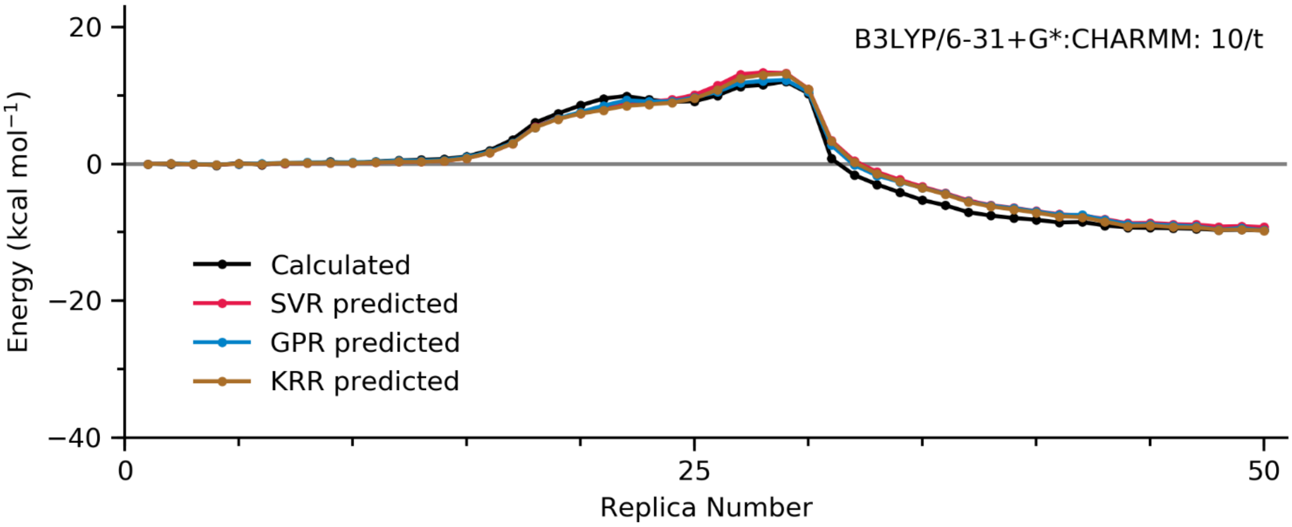
B3LYP/6-31+G*:CHARMM: 10/t
Energy (kcal mol^{-1})
Replica Number
Calculated
SVR predicted
GPR predicted
KRR predicted
20
0
−20
−40
0
25
50

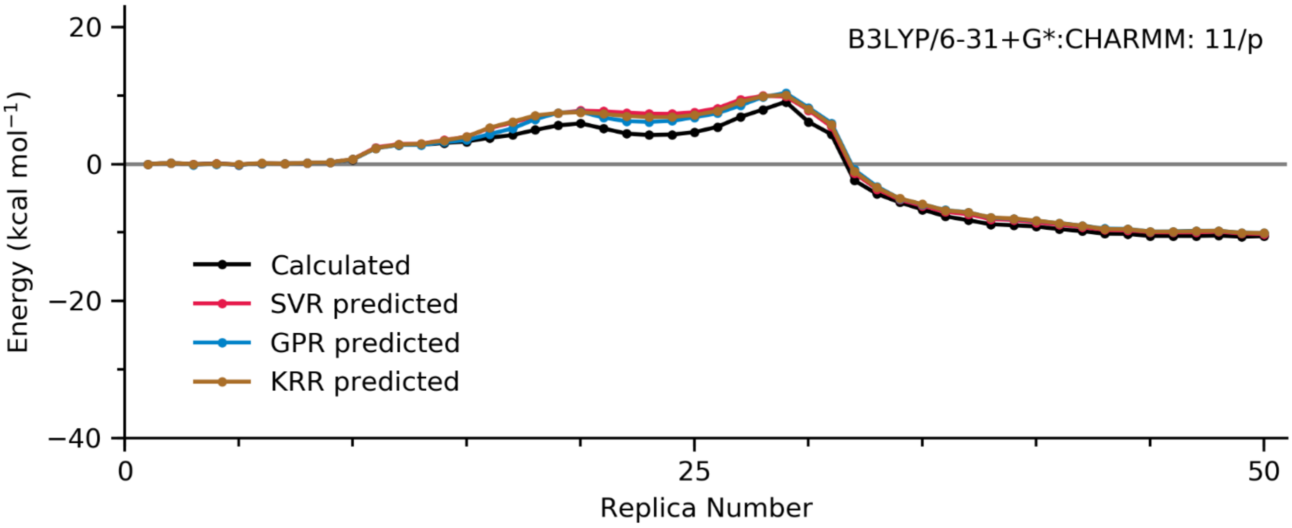
B3LYP/6-31+G*:CHARMM: 11/p
Energy (kcal mol^{-1})
Replica Number
Calculated
SVR predicted
GPR predicted
KRR predicted
20
0
−20
−40
0
25
50

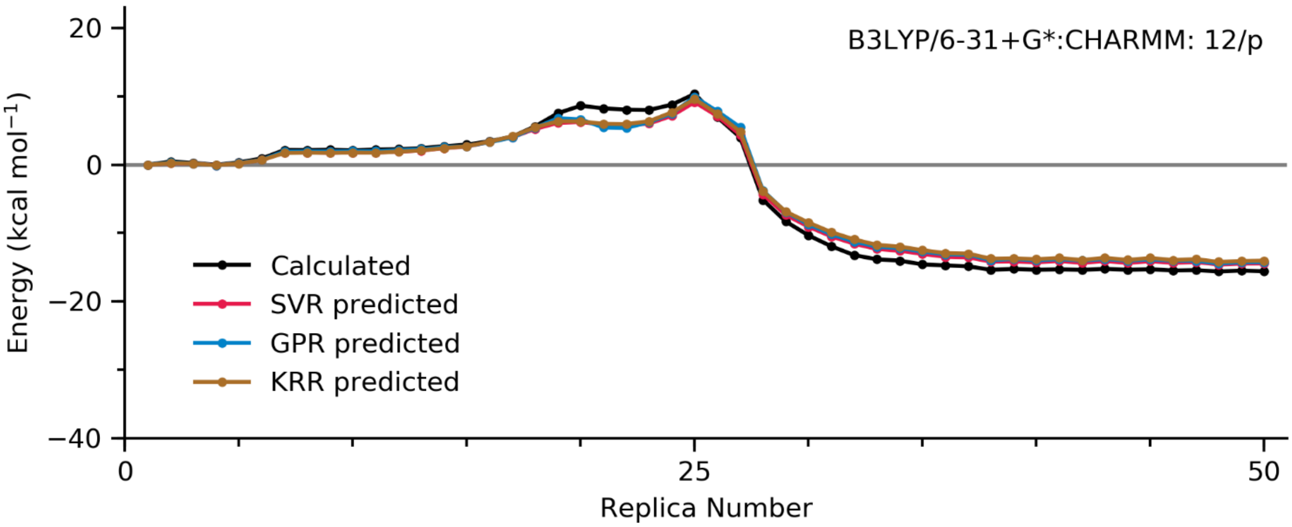
B3LYP/6-31+G*:CHARMM: 12/p
Energy (kcal mol^{-1})
Replica Number
Calculated
SVR predicted
GPR predicted
KRR predicted

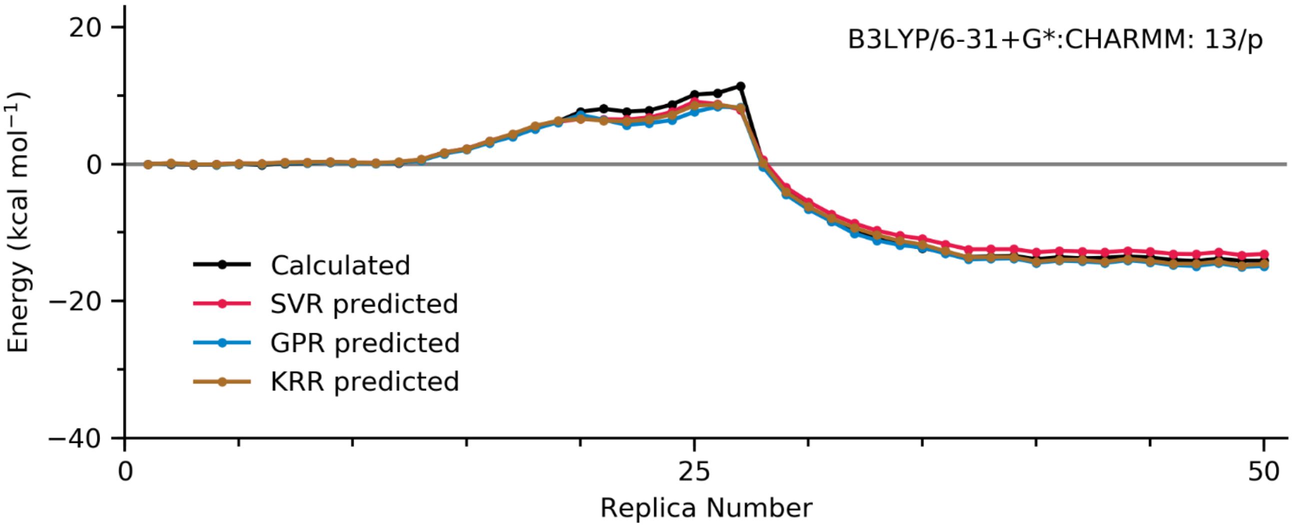
B3LYP/6-31+G*:CHARMM: 13/p
Energy (kcal mol^{-1})
Replica Number
Calculated
SVR predicted
GPR predicted
KRR predicted

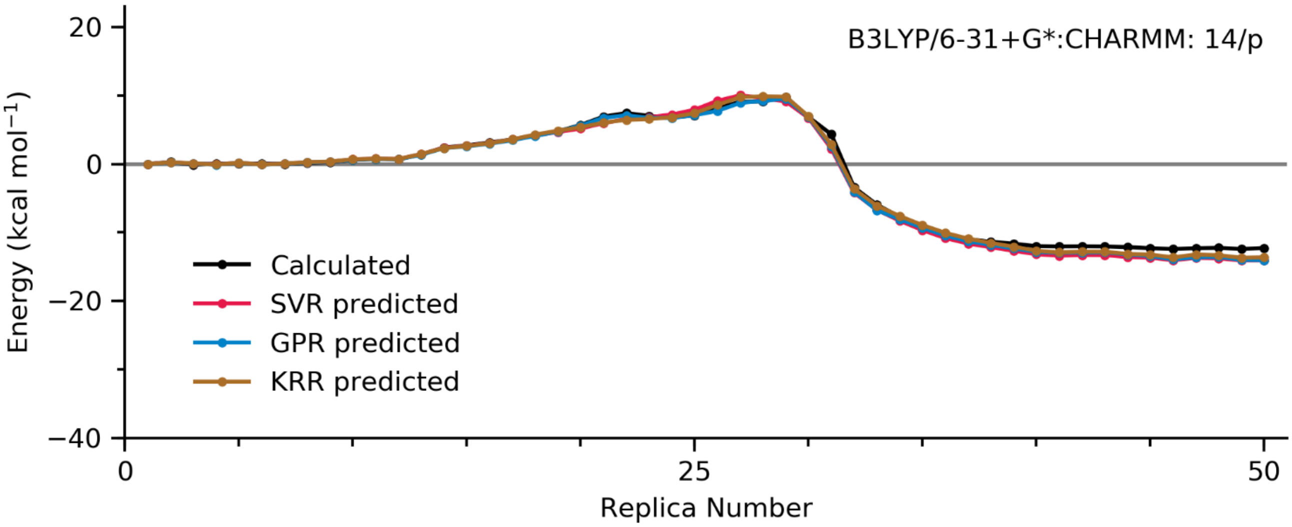
B3LYP/6-31+G*:CHARMM: 14/p
Energy (kcal mol^{-1})
Replica Number
Calculated
SVR predicted
GPR predicted
KRR predicted

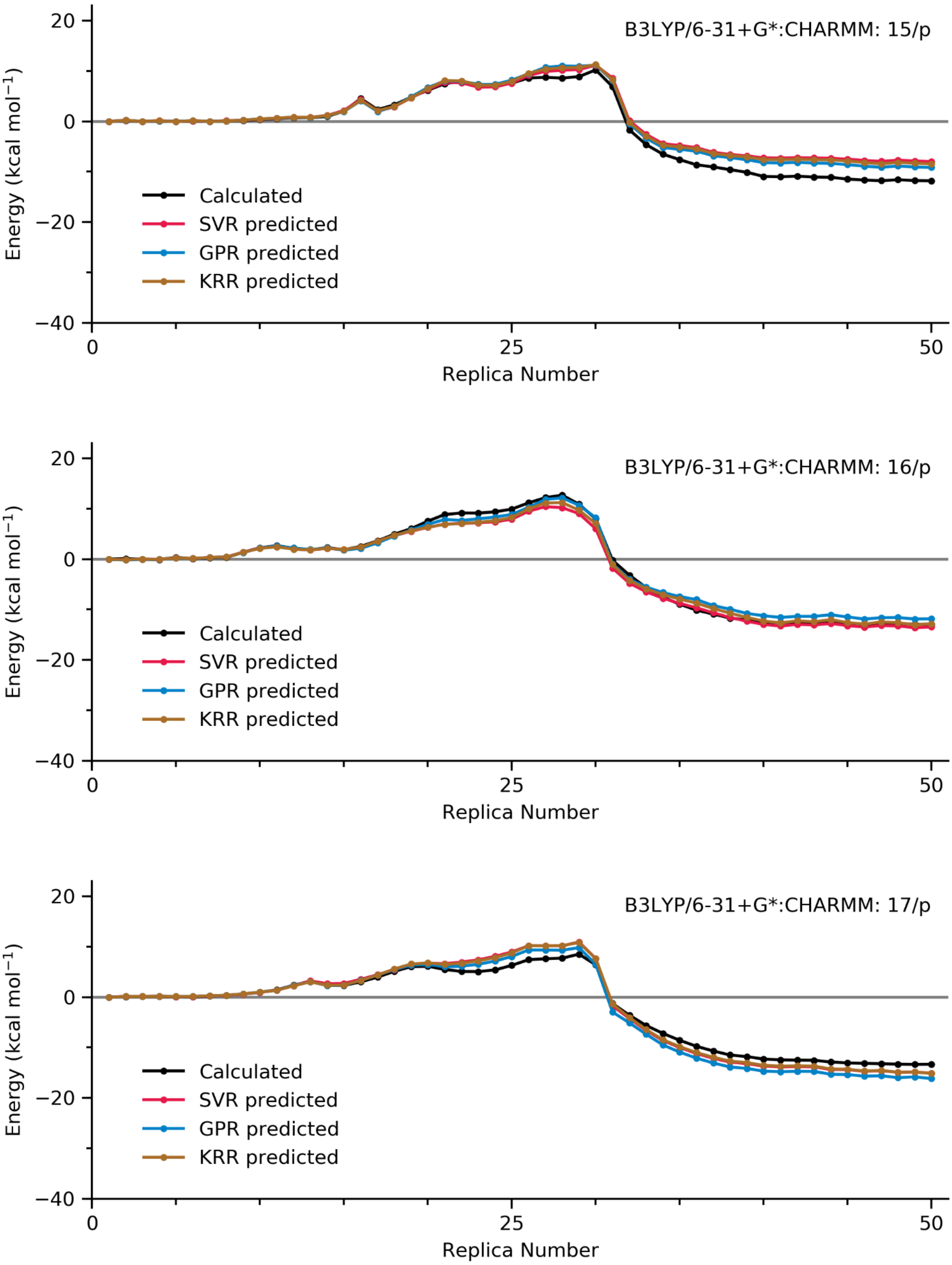
B3LYP/6-31+G*:CHARMM: 15/p
Energy (kcal mol^{-1})
Replica Number
Calculated
SVR predicted
GPR predicted
KRR predicted
B3LYP/6-31+G*:CHARMM: 16/p
Energy (kcal mol^{-1})
Replica Number
Calculated
SVR predicted
GPR predicted
KRR predicted
B3LYP/6-31+G*:CHARMM: 17/p
Energy (kcal mol^{-1})
Replica Number
Calculated
SVR predicted
GPR predicted
KRR predicted

Supplementary Figure 71 to 88. B3LYP/6-31++G**:CHARMM pathways

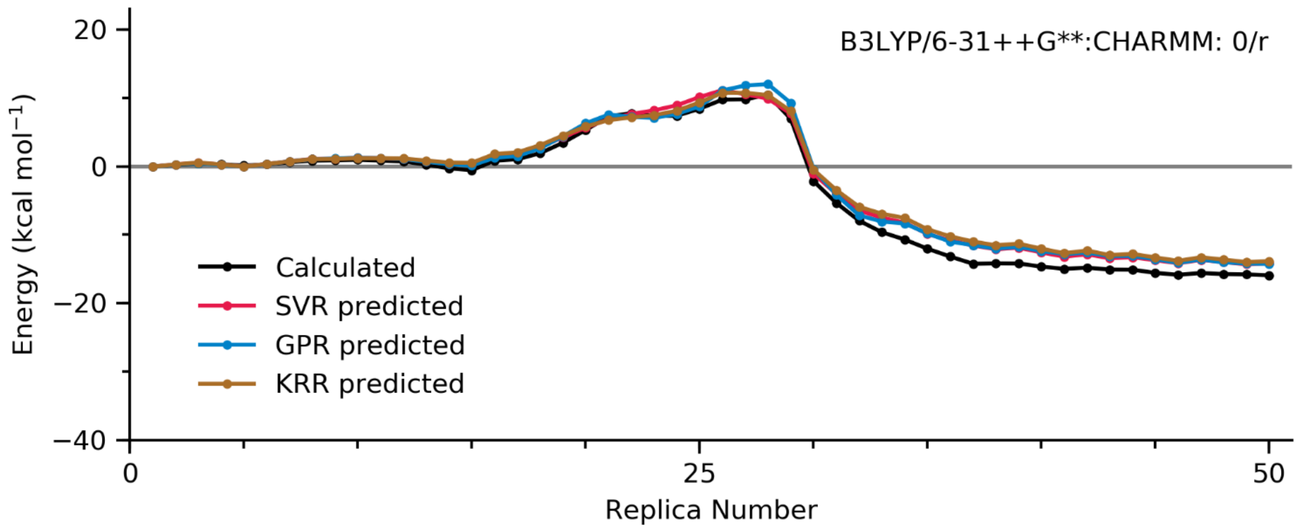

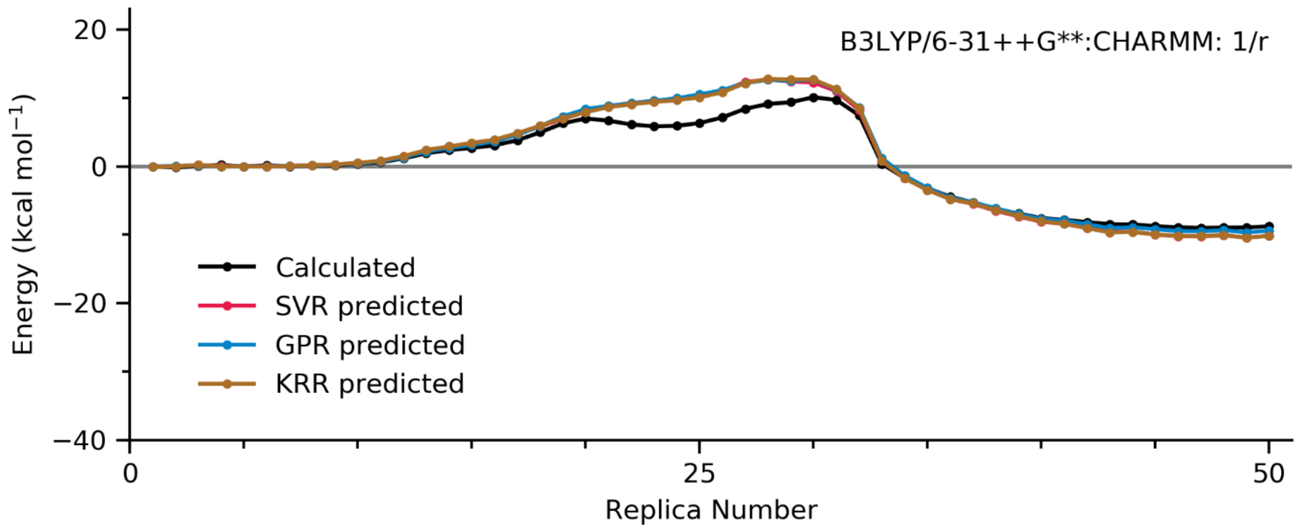

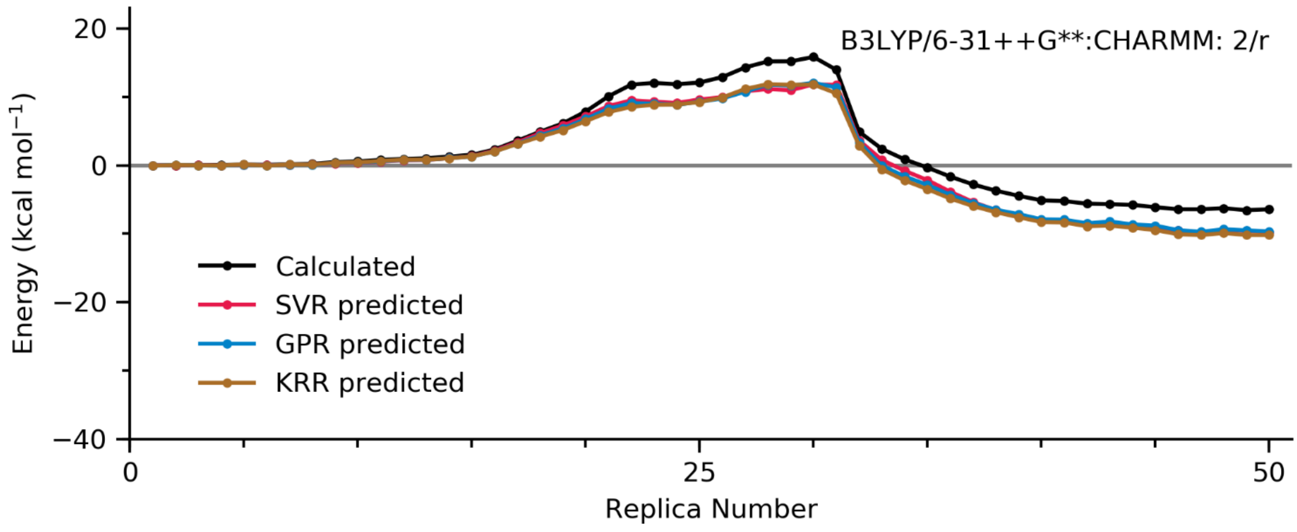

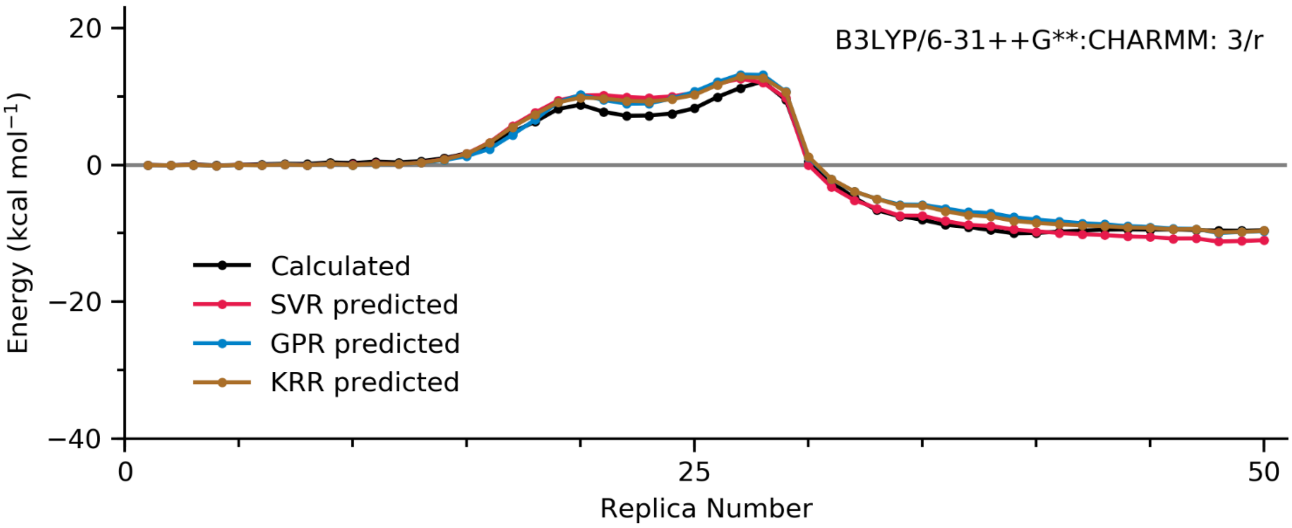

B3LYP/6-31++G**:CHARMM: 3/r
Energy (kcal mol^{-1})
Replica Number
Calculated
SVR predicted
GPR predicted
KRR predicted

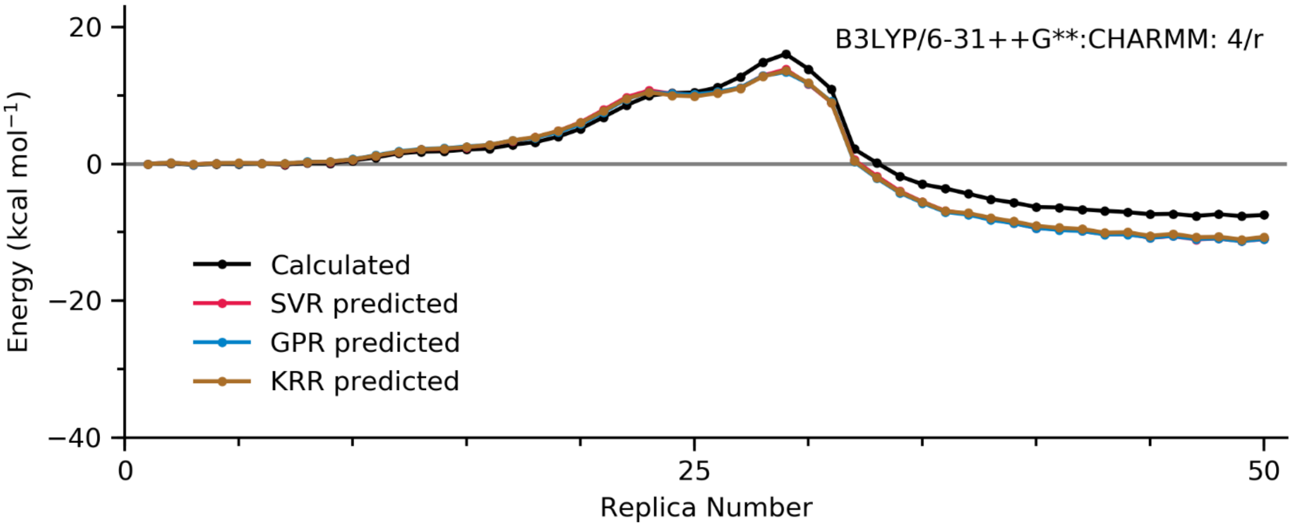

B3LYP/6-31++G**:CHARMM: 4/r
Energy (kcal mol^{-1})
Replica Number
Calculated
SVR predicted
GPR predicted
KRR predicted

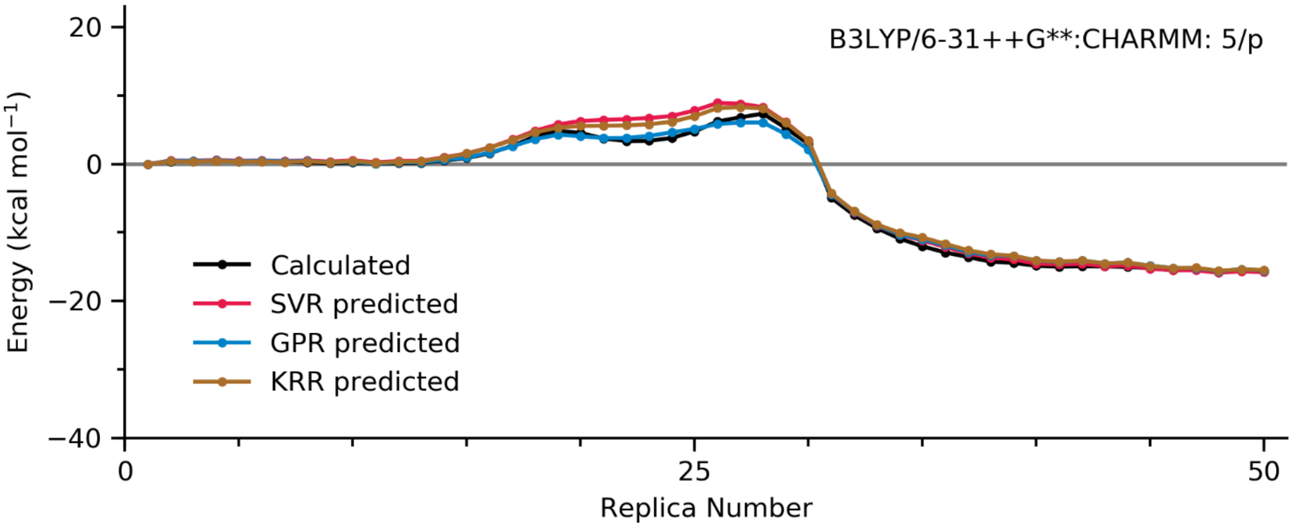

B3LYP/6-31++G**:CHARMM: 5/p
Energy (kcal mol^{-1})
Replica Number
Calculated
SVR predicted
GPR predicted
KRR predicted

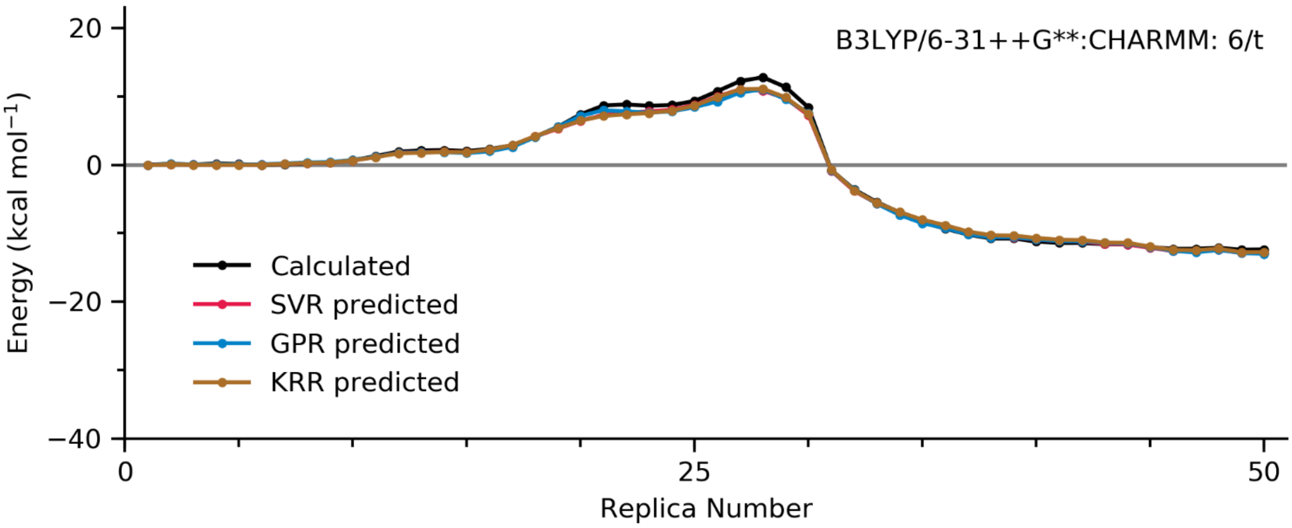

B3LYP/6-31++G**:CHARMM: 6/t
Energy (kcal mol^{-1})
Replica Number
Calculated
SVR predicted
GPR predicted
KRR predicted

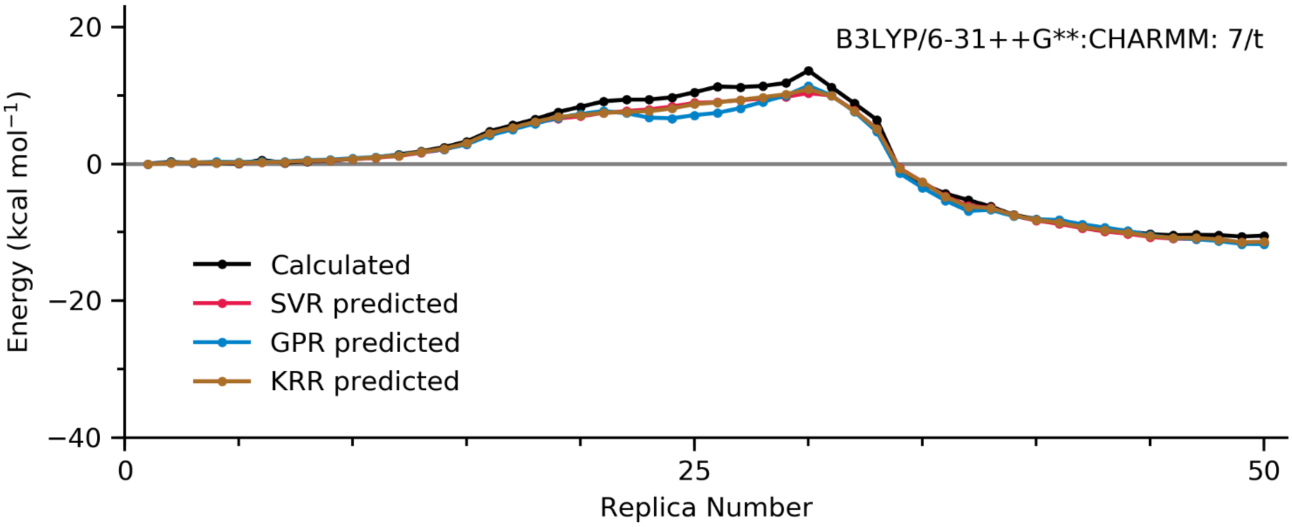

B3LYP/6-31++G**:CHARMM: 7/t
Energy (kcal mol^{-1})
Replica Number
Calculated
SVR predicted
GPR predicted
KRR predicted

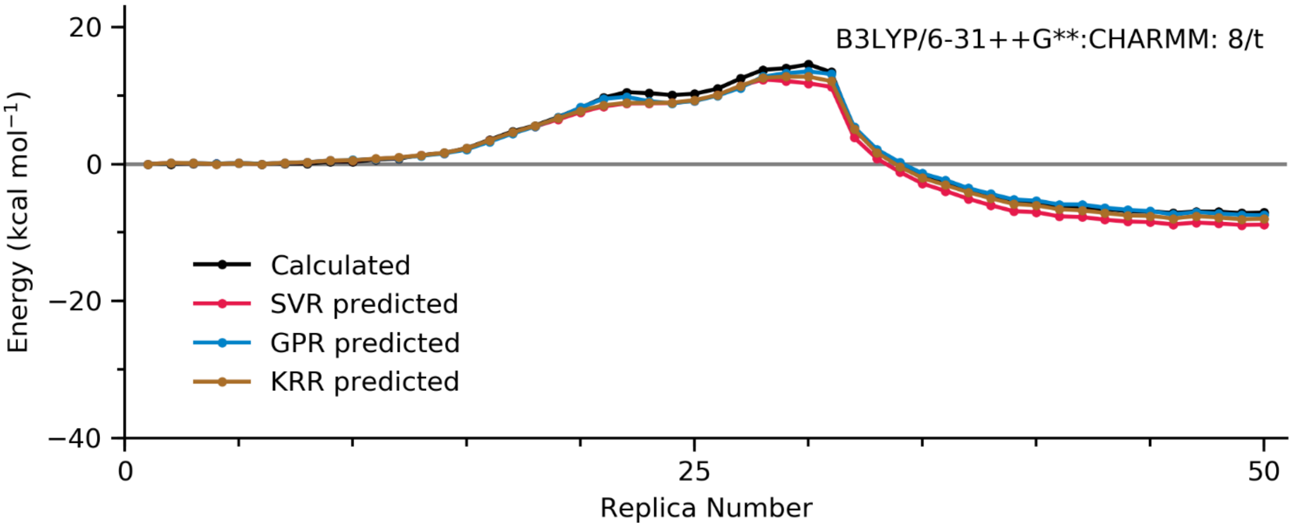

B3LYP/6-31++G**:CHARMM: 8/t
Energy (kcal mol^{-1})
Replica Number
Calculated
SVR predicted
GPR predicted
KRR predicted

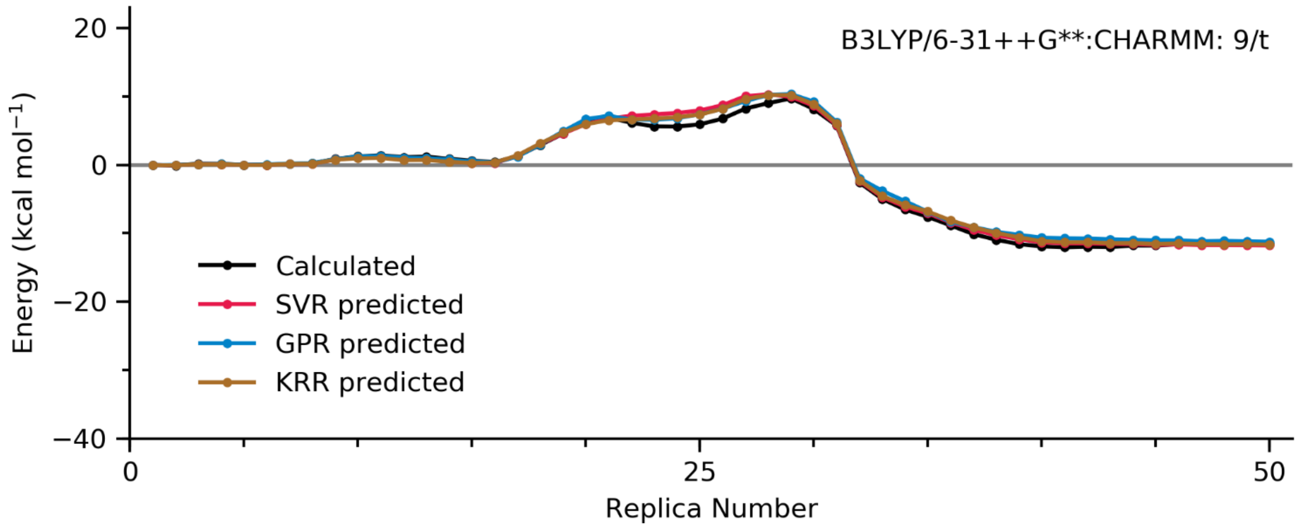
B3LYP/6-31++G**:CHARMM: 9/t
Calculated
SVR predicted
GPR predicted
KRR predicted
Energy (kcal mol−1)
Replica Number
20
0
−20
−40
0
25
50

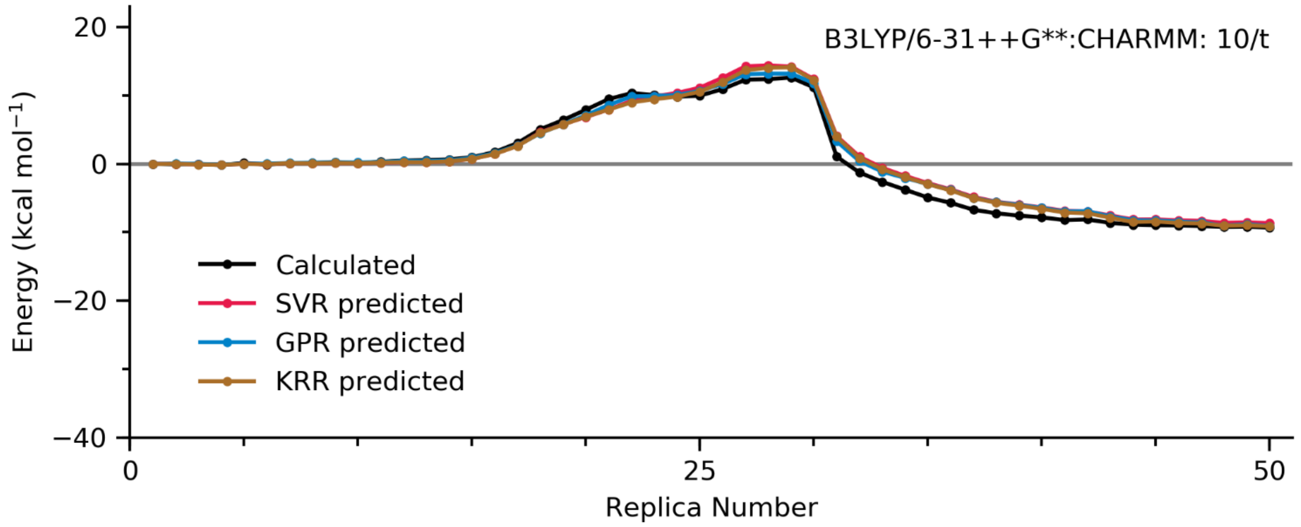
B3LYP/6-31++G**:CHARMM: 10/t
Calculated
SVR predicted
GPR predicted
KRR predicted
Energy (kcal mol−1)
Replica Number
20
0
−20
−40
0
25
50

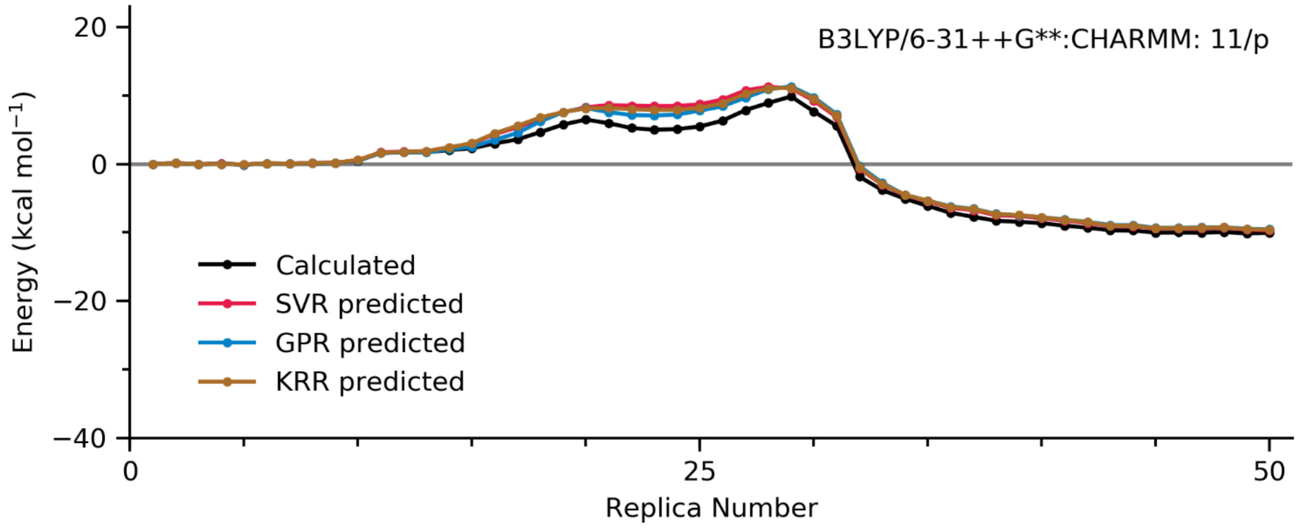
B3LYP/6-31++G**:CHARMM: 11/p
Calculated
SVR predicted
GPR predicted
KRR predicted
Energy (kcal mol−1)
Replica Number
20
0
−20
−40
0
25
50

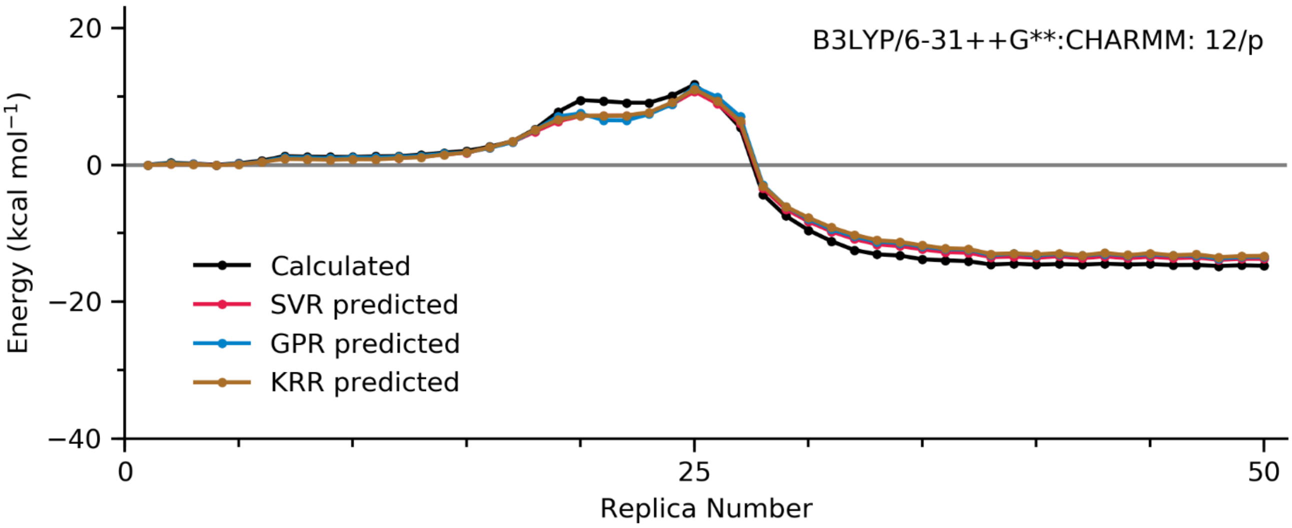
B3LYP/6-31++G**:CHARMM: 12/p
Energy (kcal mol^{-1})
Replica Number
Calculated
SVR predicted
GPR predicted
KRR predicted
20
0
−20
−40
0
25
50

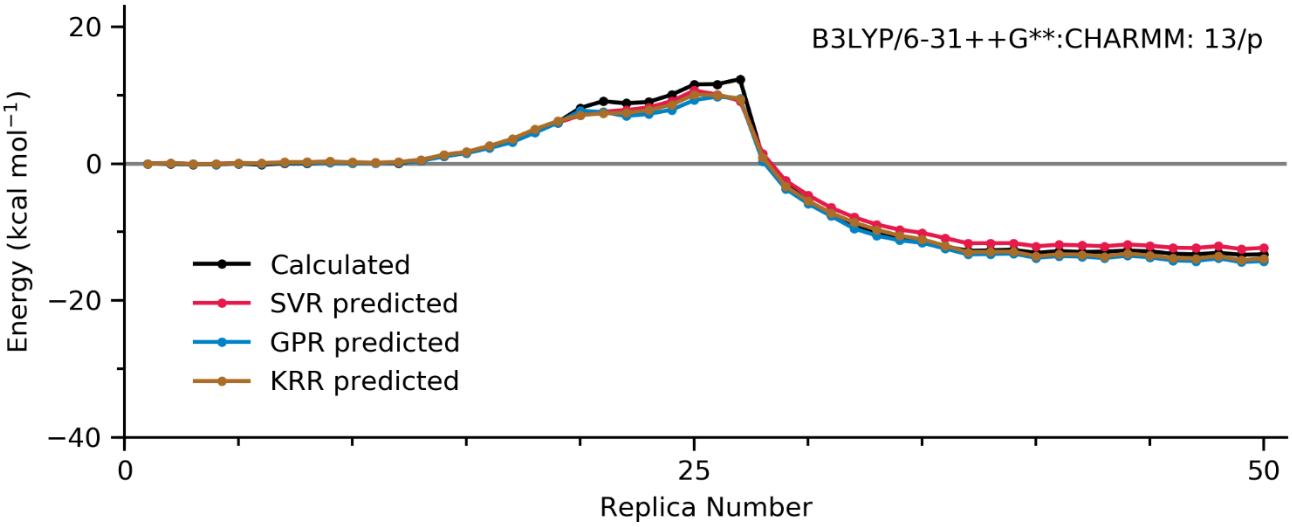
B3LYP/6-31++G**:CHARMM: 13/p
Energy (kcal mol^{-1})
Replica Number
Calculated
SVR predicted
GPR predicted
KRR predicted
20
0
−20
−40
0
25
50

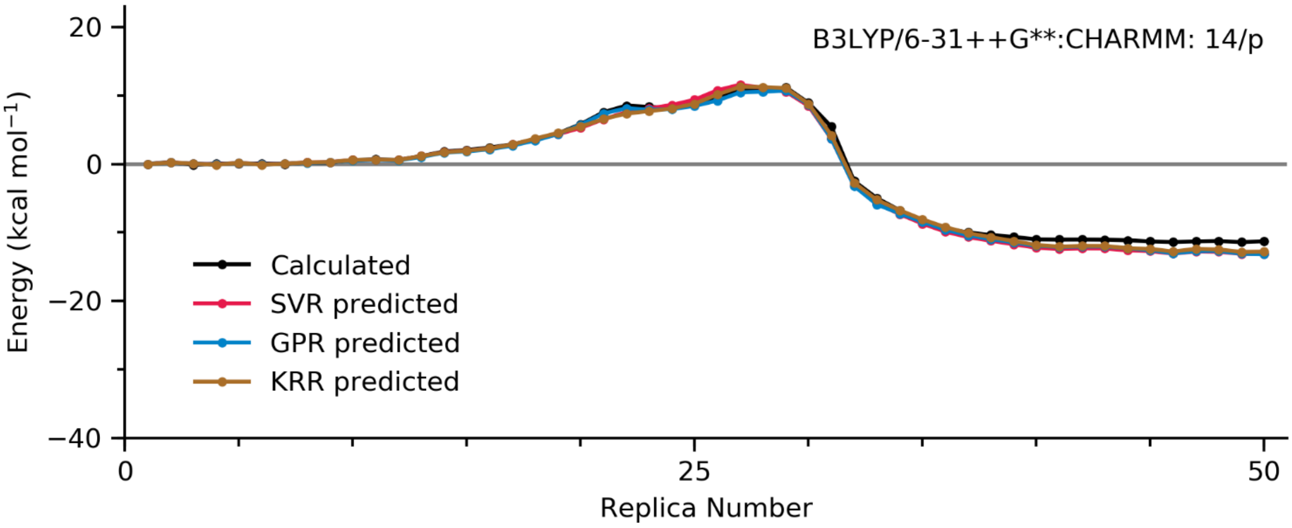
B3LYP/6-31++G**:CHARMM: 14/p
Energy (kcal mol^{-1})
Replica Number
Calculated
SVR predicted
GPR predicted
KRR predicted
20
0
−20
−40
0
25
50

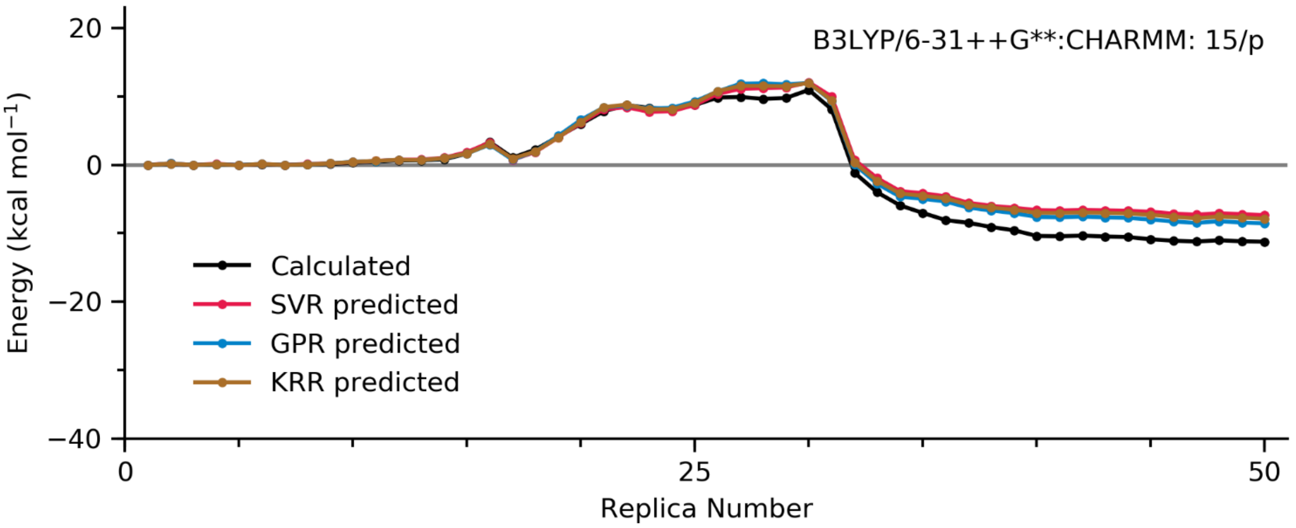

B3LYP/6-31++G**:CHARMM: 15/p
Energy (kcal mol^{-1})
Replica Number
Calculated
SVR predicted
GPR predicted
KRR predicted

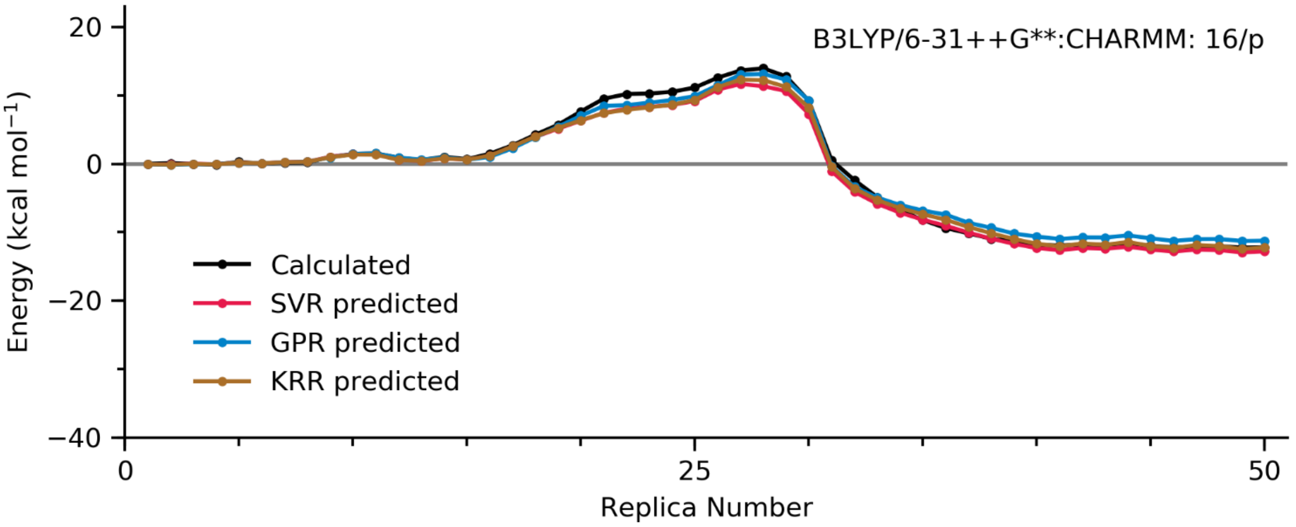

B3LYP/6-31++G**:CHARMM: 16/p
Energy (kcal mol^{-1})
Replica Number
Calculated
SVR predicted
GPR predicted
KRR predicted

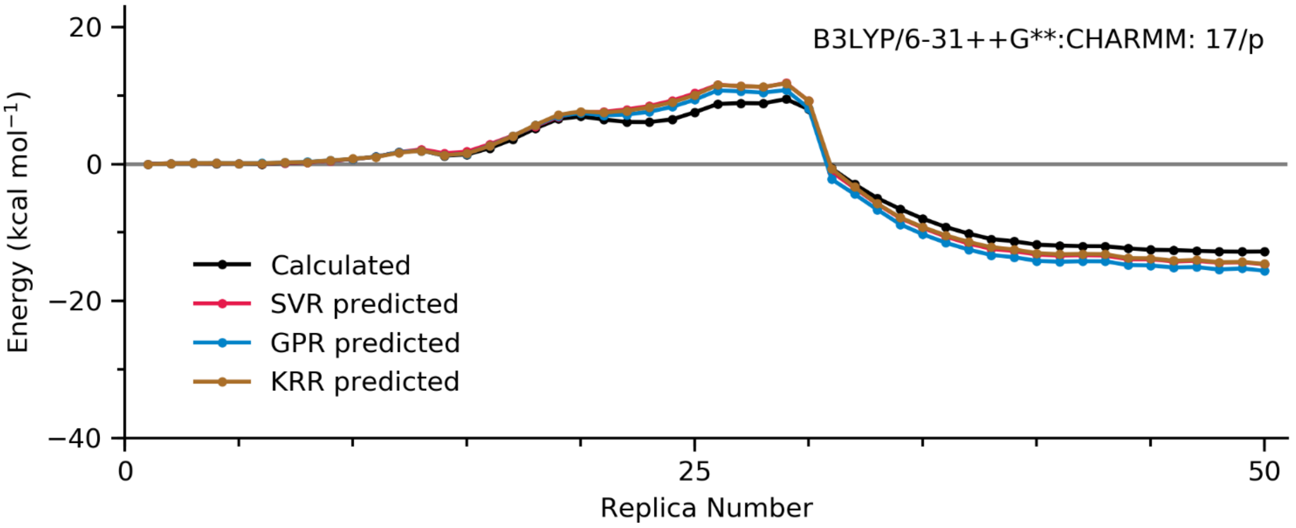

B3LYP/6-31++G**:CHARMM: 17/p
Energy (kcal mol^{-1})
Replica Number
Calculated
SVR predicted
GPR predicted
KRR predicted

Supplementary Figure 89 to 106. B3LYP-D3/6-31++G**:CHARMM pathways

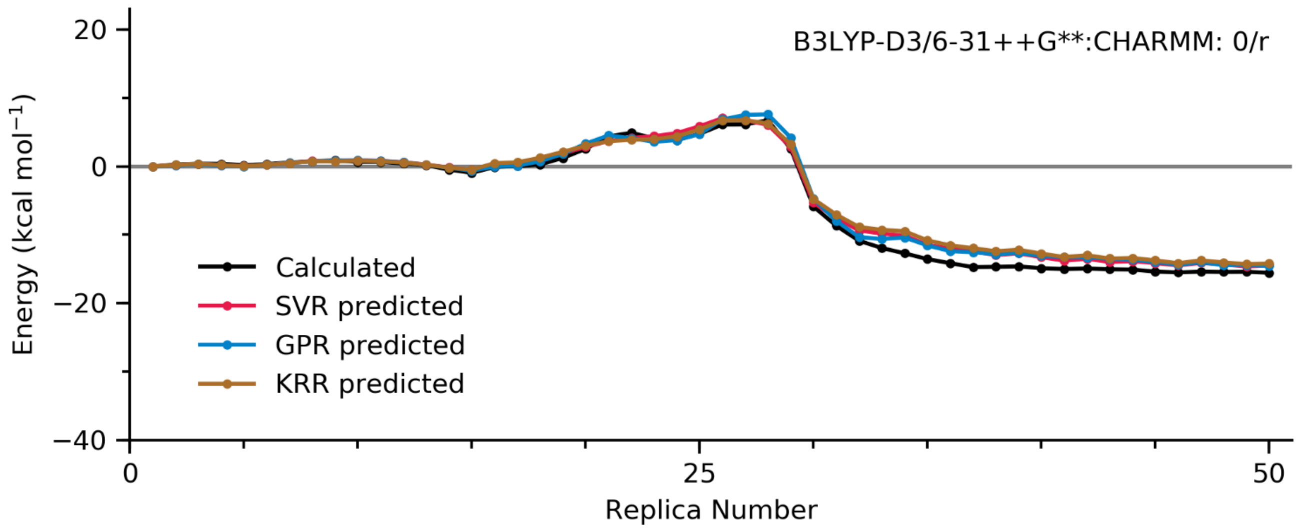

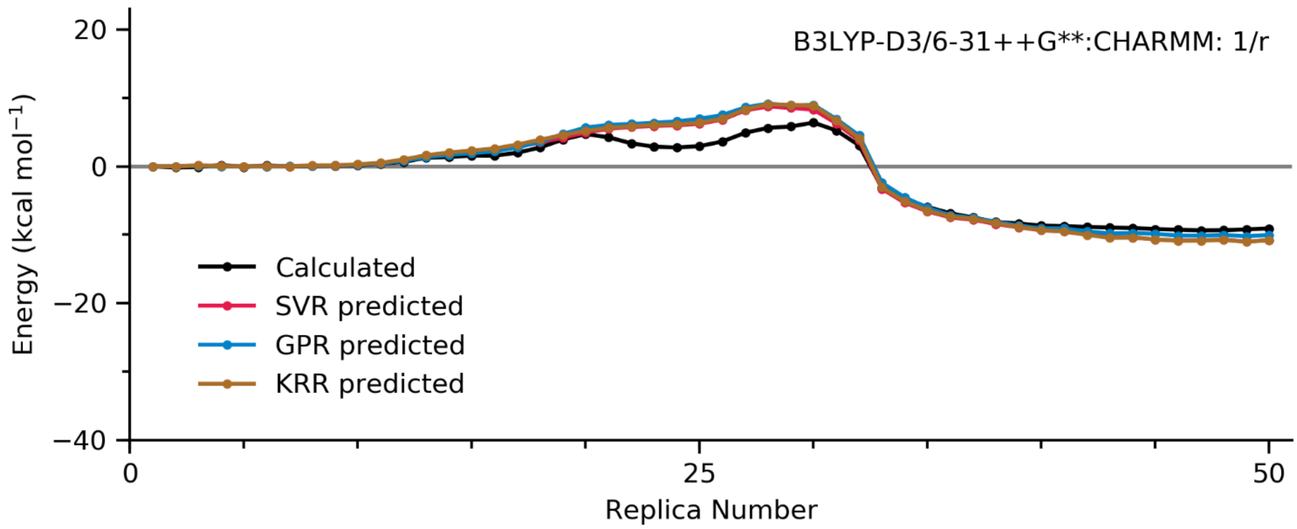

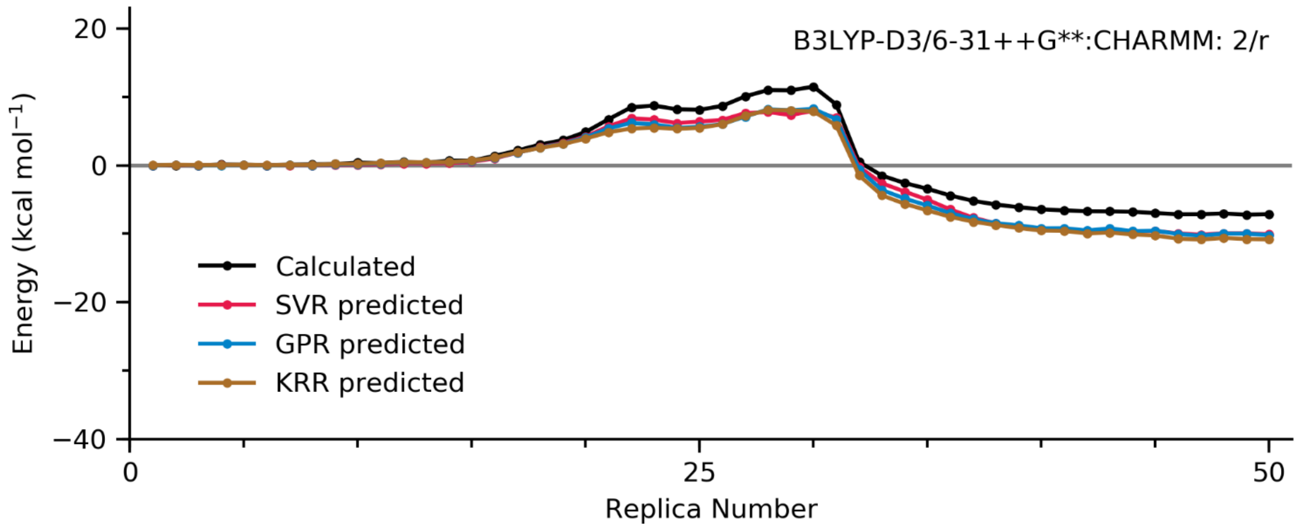

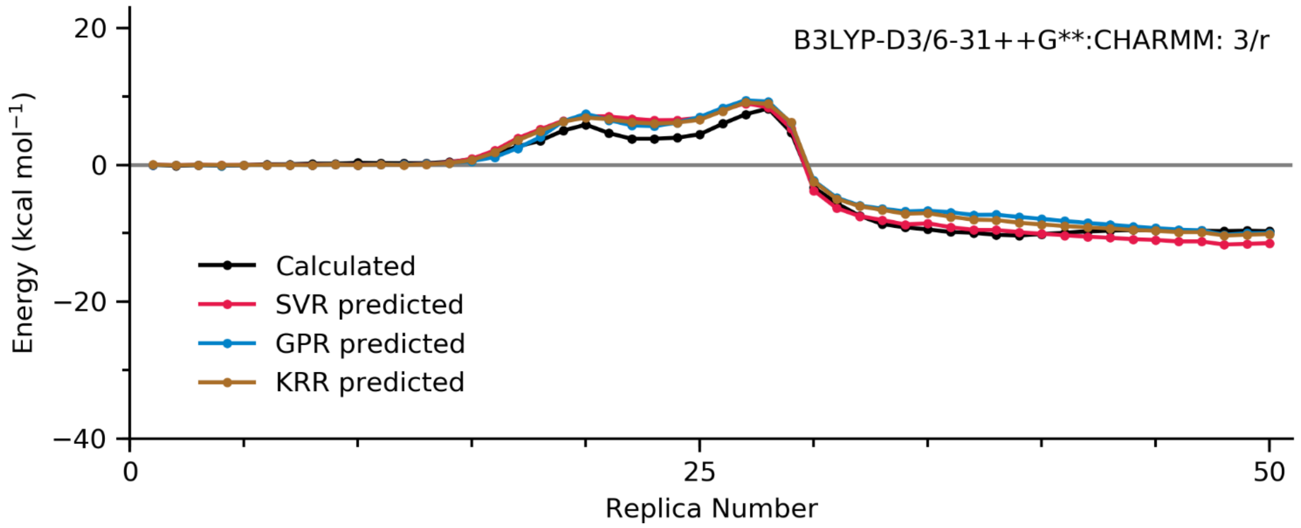
B3LYP-D3/6-31++G**:CHARMM: 3/r
Calculated
SVR predicted
GPR predicted
KRR predicted
Energy (kcal mol−1)
Replica Number
20
0
−20
−40
0
25
50

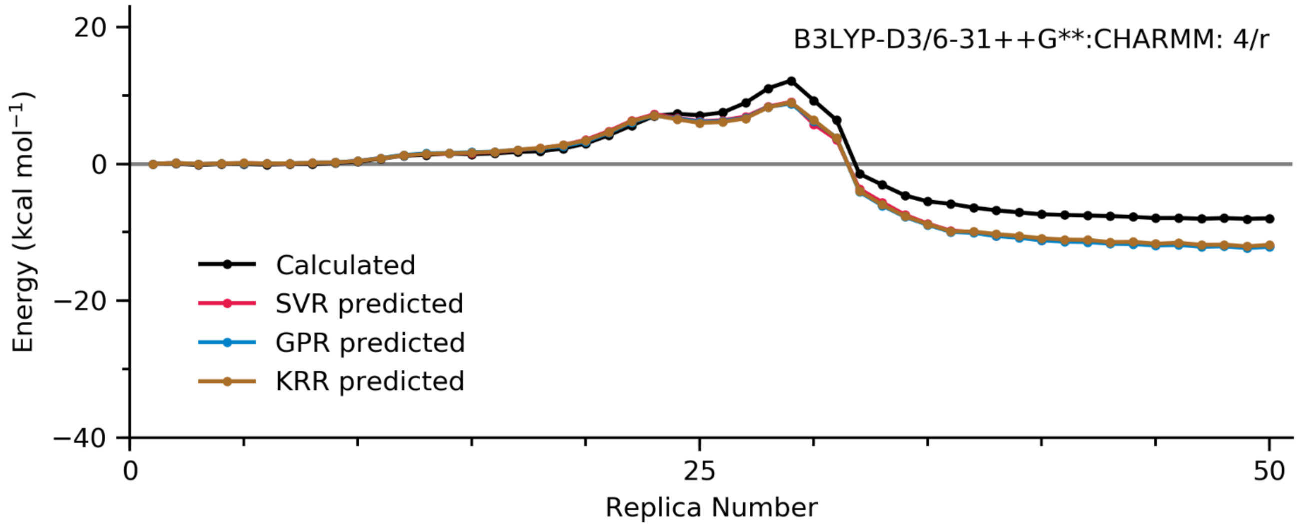
B3LYP-D3/6-31++G**:CHARMM: 4/r
Calculated
SVR predicted
GPR predicted
KRR predicted
Energy (kcal mol−1)
Replica Number
20
0
−20
−40
0
25
50

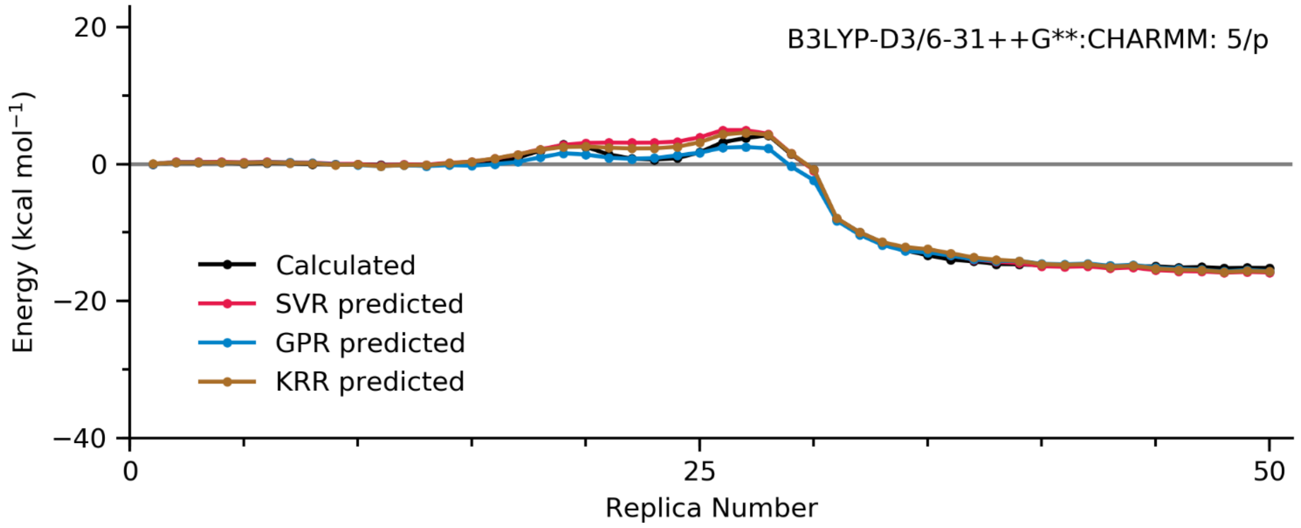
B3LYP-D3/6-31++G**:CHARMM: 5/p
Calculated
SVR predicted
GPR predicted
KRR predicted
Energy (kcal mol−1)
Replica Number
20
0
−20
−40
0
25
50

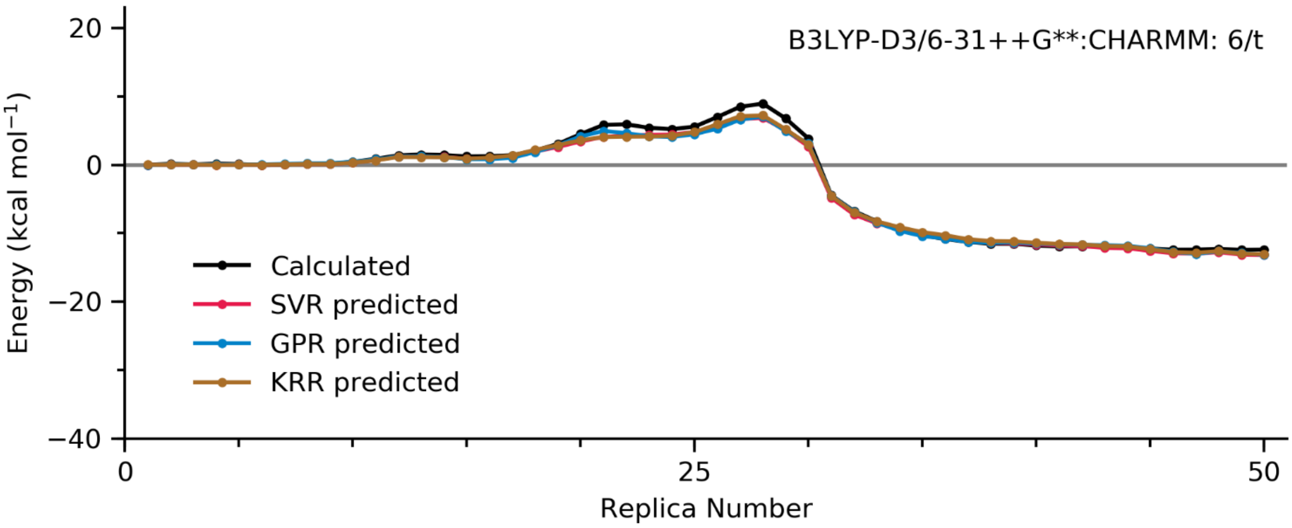
B3LYP-D3/6-31++G**:CHARMM: 6/t
Energy (kcal mol−1)
20
0
−20
−40
0
25
50
Replica Number
Calculated
SVR predicted
GPR predicted
KRR predicted

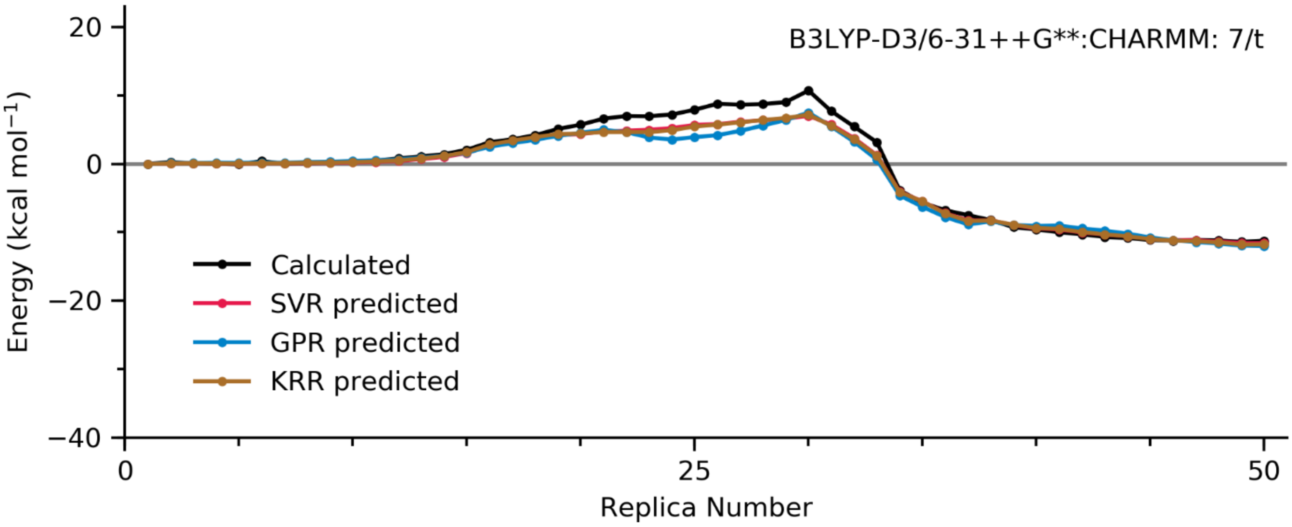
B3LYP-D3/6-31++G**:CHARMM: 7/t
Energy (kcal mol−1)
20
0
−20
−40
0
25
50
Replica Number
Calculated
SVR predicted
GPR predicted
KRR predicted

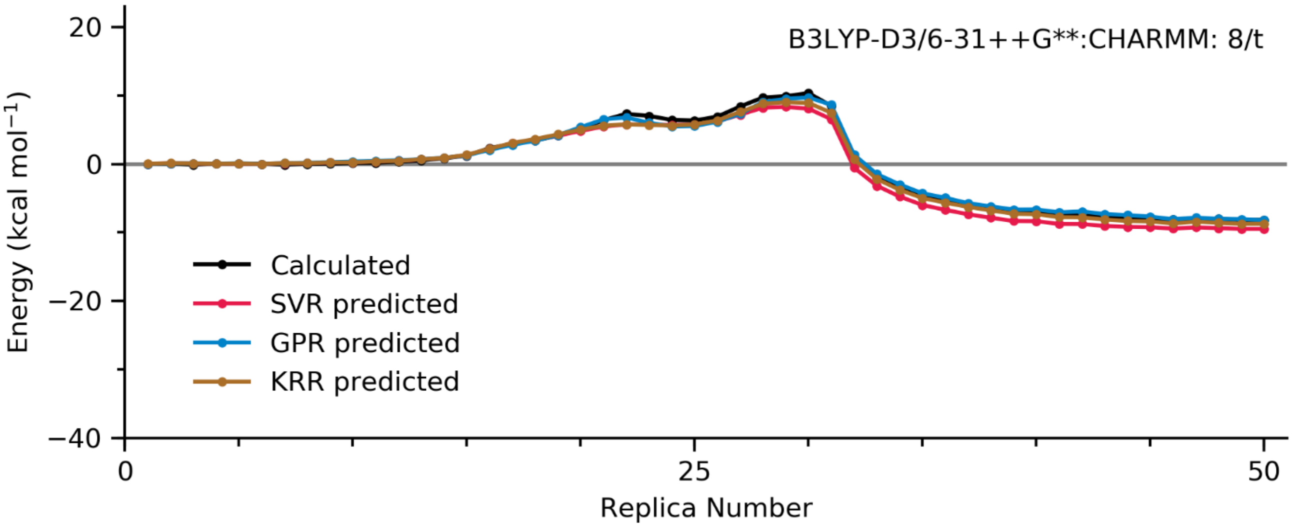
B3LYP-D3/6-31++G**:CHARMM: 8/t
Energy (kcal mol−1)
20
0
−20
−40
0
25
50
Replica Number
Calculated
SVR predicted
GPR predicted
KRR predicted

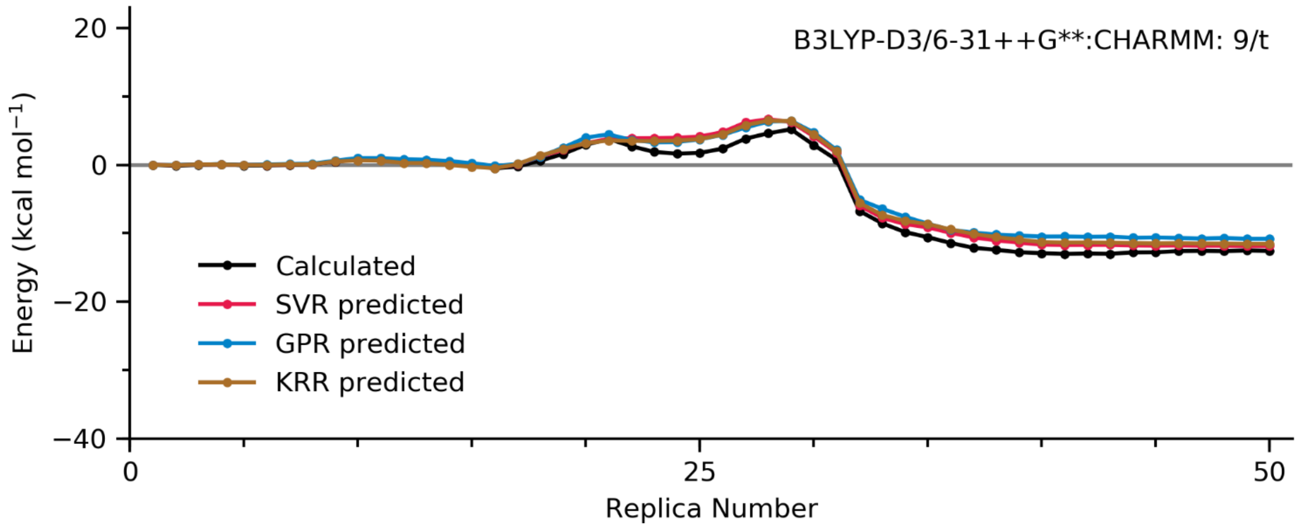
B3LYP-D3/6-31++G**:CHARMM: 9/t
Energy (kcal mol^{-1})
20
0
−20
−40
0
25
50
Replica Number
Calculated
SVR predicted
GPR predicted
KRR predicted

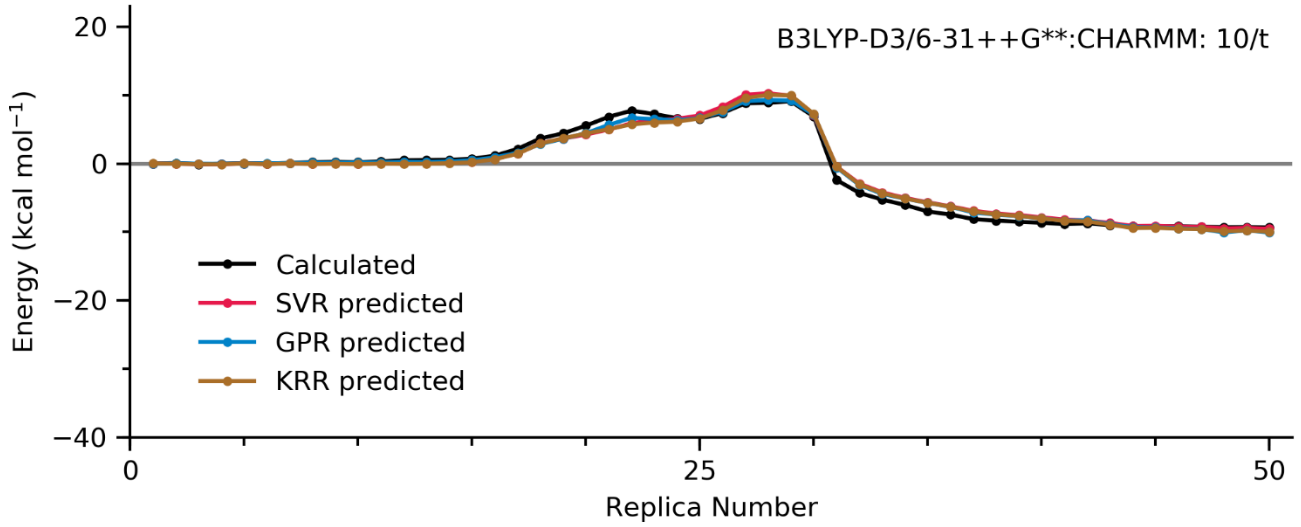
B3LYP-D3/6-31++G**:CHARMM: 10/t
Energy (kcal mol^{-1})
20
0
−20
−40
0
25
50
Replica Number
Calculated
SVR predicted
GPR predicted
KRR predicted

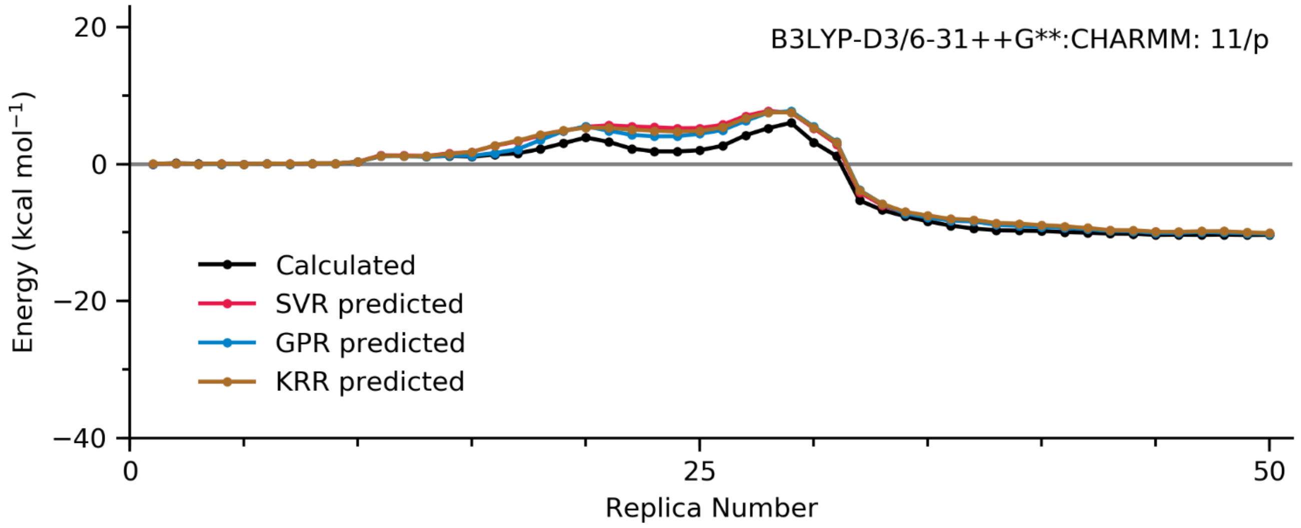
B3LYP-D3/6-31++G**:CHARMM: 11/p
Energy (kcal mol^{-1})
20
0
−20
−40
0
25
50
Replica Number
Calculated
SVR predicted
GPR predicted
KRR predicted

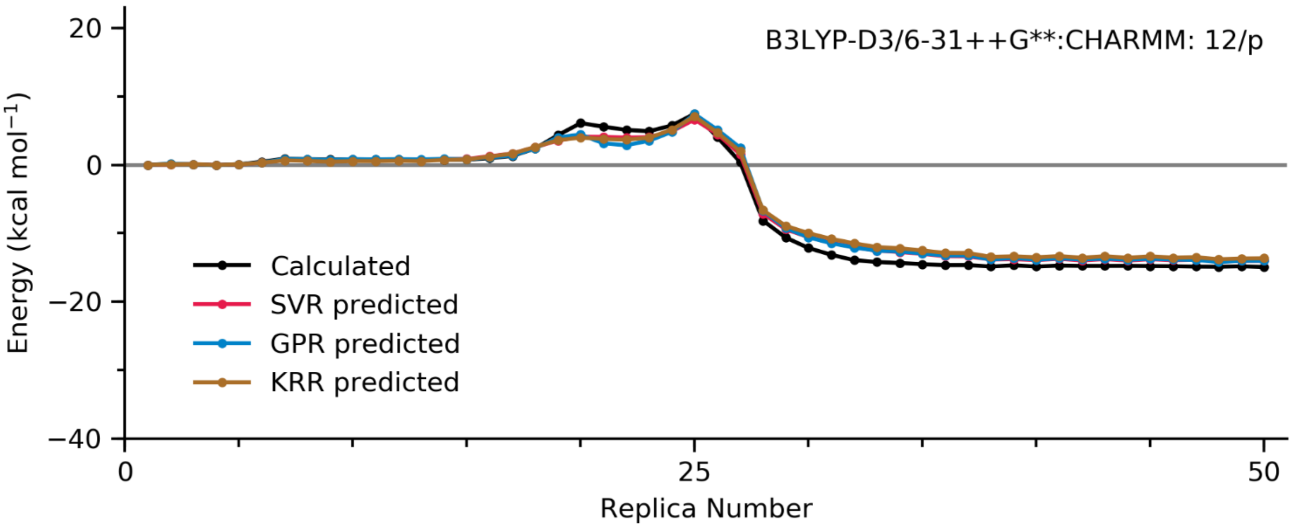

B3LYP-D3/6-31++G**:CHARMM: 12/p
Calculated
SVR predicted
GPR predicted
KRR predicted
Energy (kcal mol−1)
Replica Number
20
0
−20
−40
0
25
50

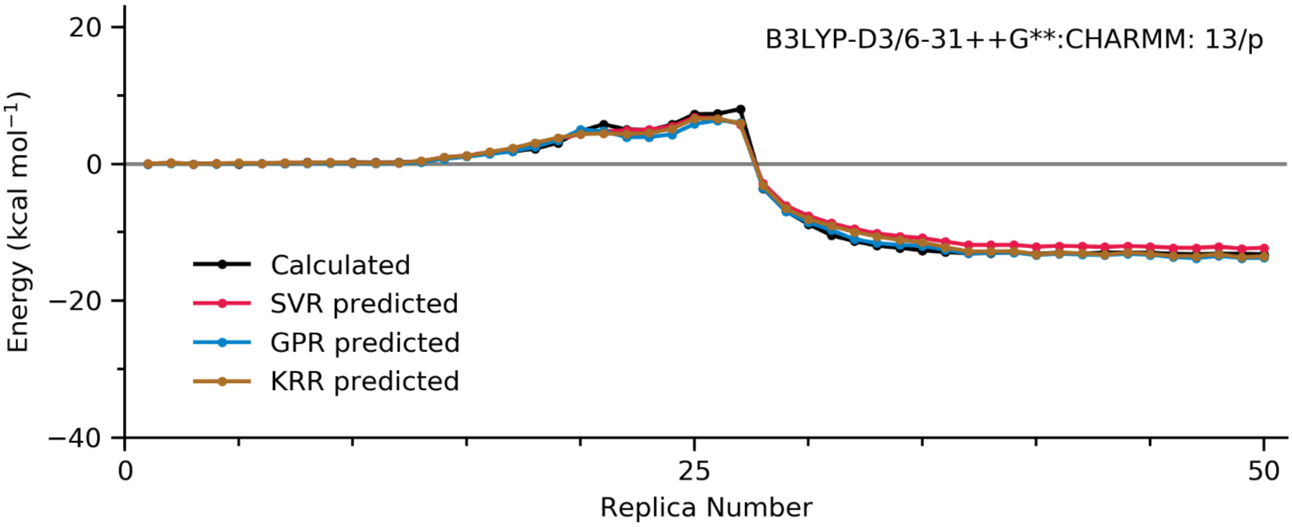

B3LYP-D3/6-31++G**:CHARMM: 13/p
Calculated
SVR predicted
GPR predicted
KRR predicted
Energy (kcal mol−1)
Replica Number
20
0
−20
−40
0
25
50

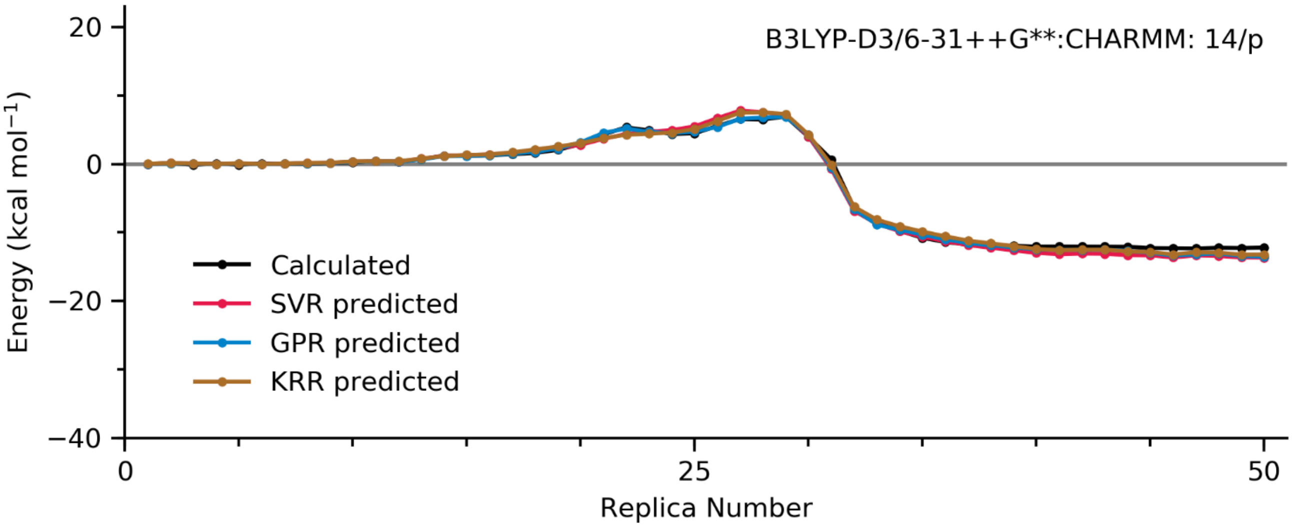

B3LYP-D3/6-31++G**:CHARMM: 14/p
Calculated
SVR predicted
GPR predicted
KRR predicted
Energy (kcal mol−1)
Replica Number
20
0
−20
−40
0
25
50

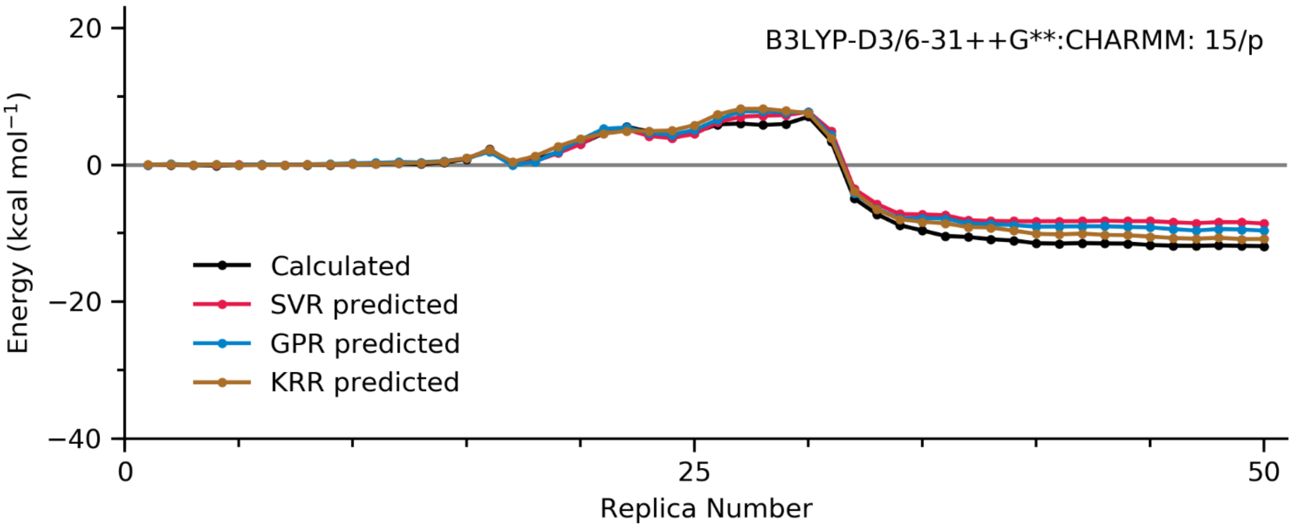
B3LYP-D3/6-31++G**:CHARMM: 15/p
Energy (kcal mol^{-1})
Replica Number
Calculated
SVR predicted
GPR predicted
KRR predicted
20
0
−20
−40
0
25
50

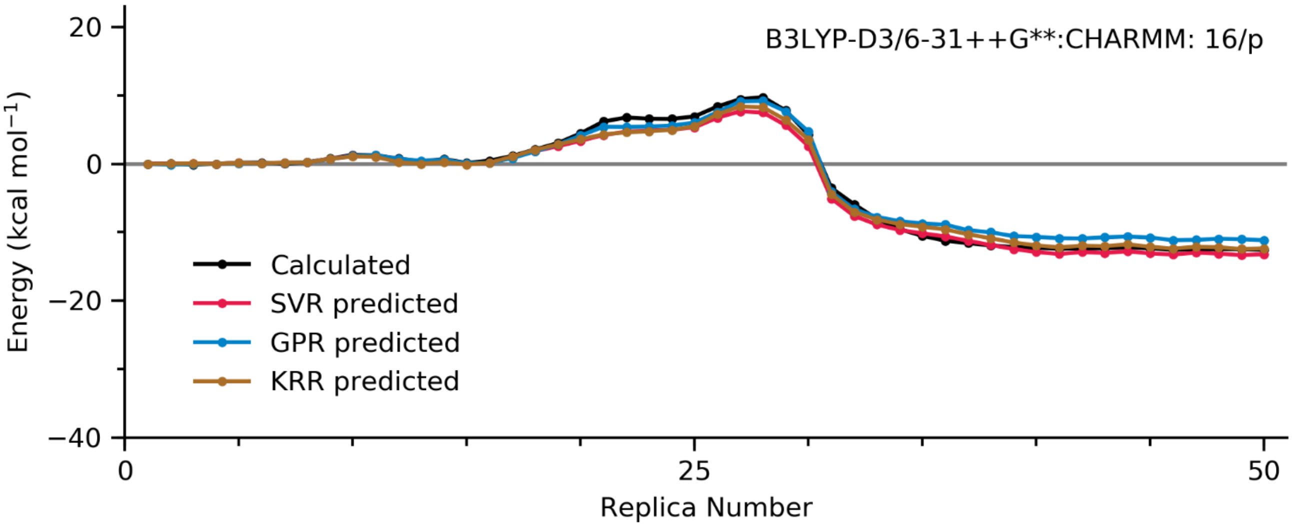
B3LYP-D3/6-31++G**:CHARMM: 16/p
Energy (kcal mol^{-1})
Replica Number
Calculated
SVR predicted
GPR predicted
KRR predicted
20
0
−20
−40
0
25
50

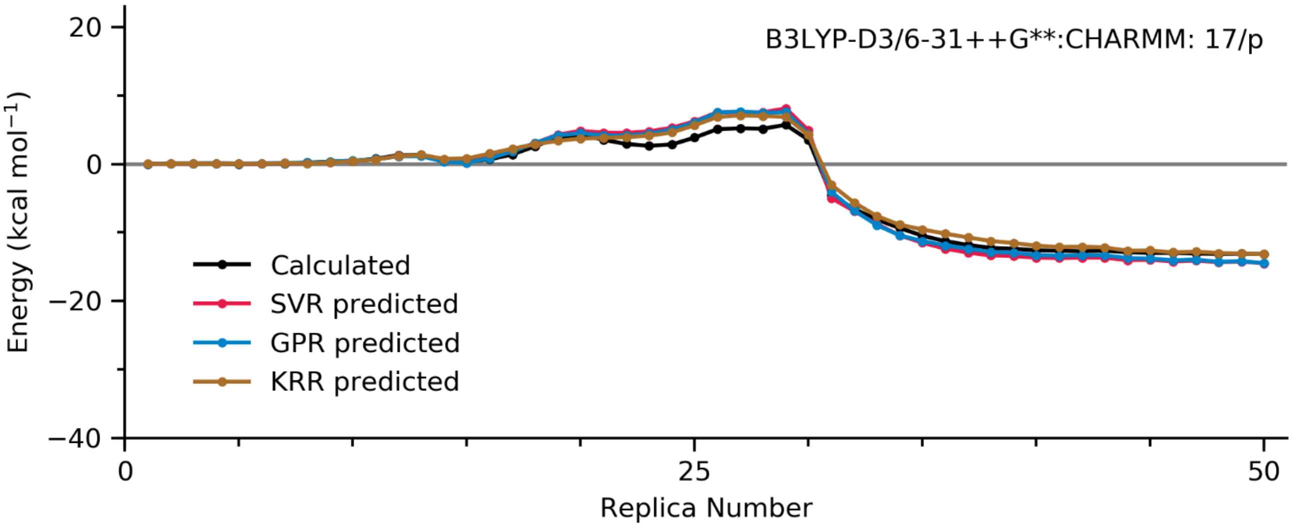
B3LYP-D3/6-31++G**:CHARMM: 17/p
Energy (kcal mol^{-1})
Replica Number
Calculated
SVR predicted
GPR predicted
KRR predicted
20
0
−20
−40
0
25
50

Supplementary Figure 107 to 124. B3LYP/6-311++G**:CHARMM pathways

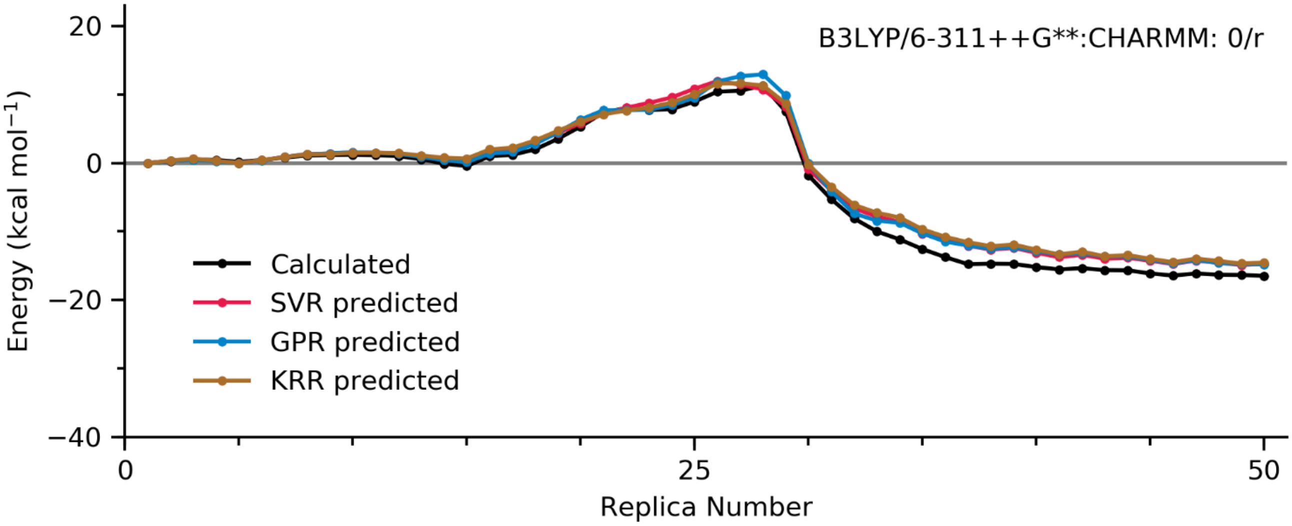

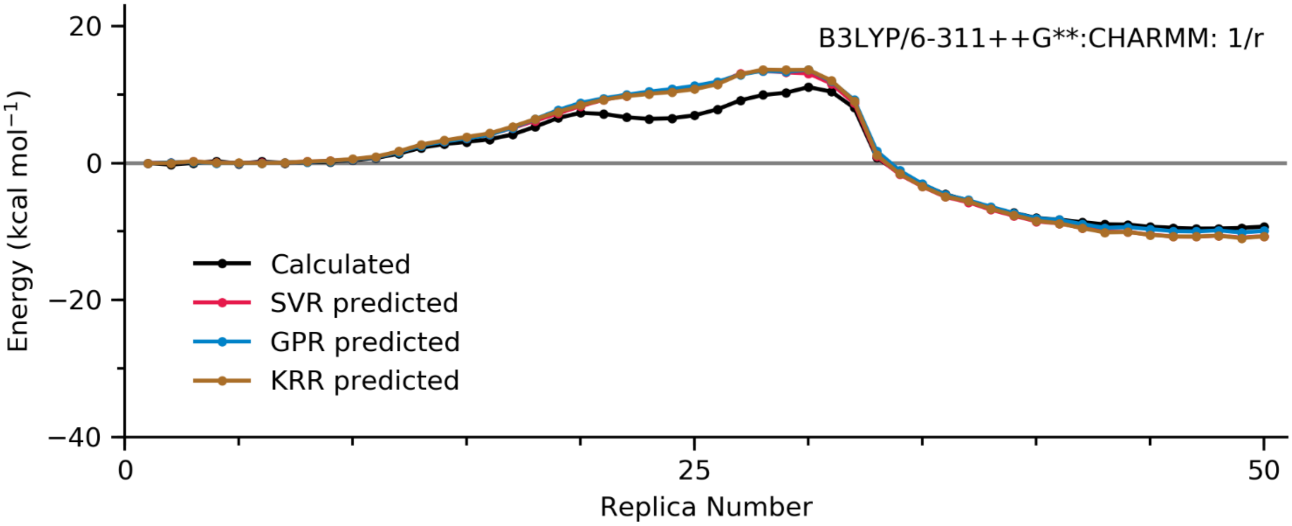

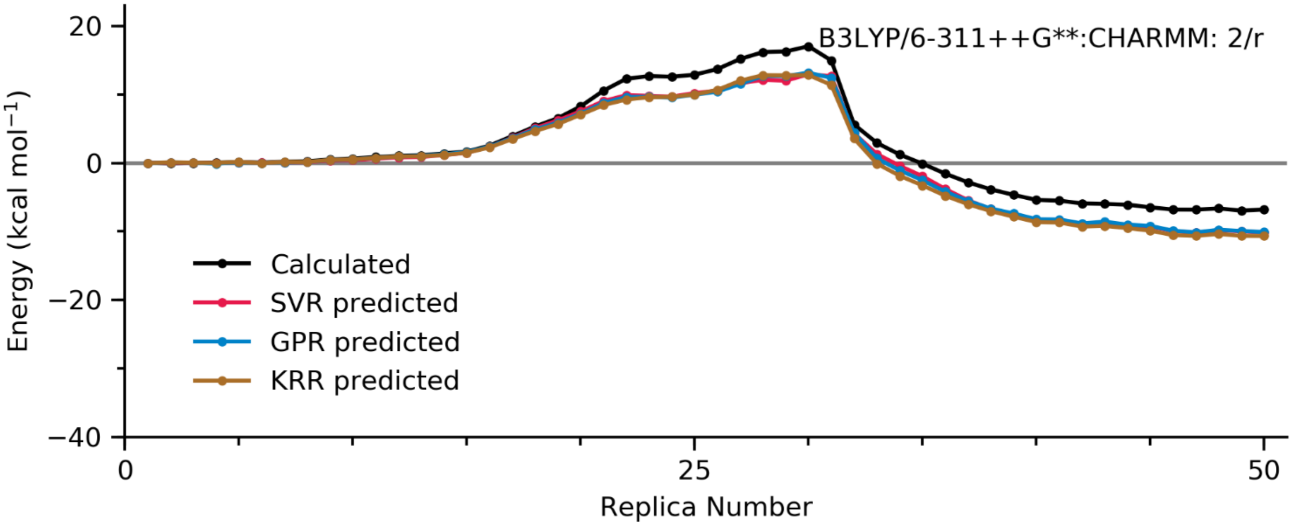

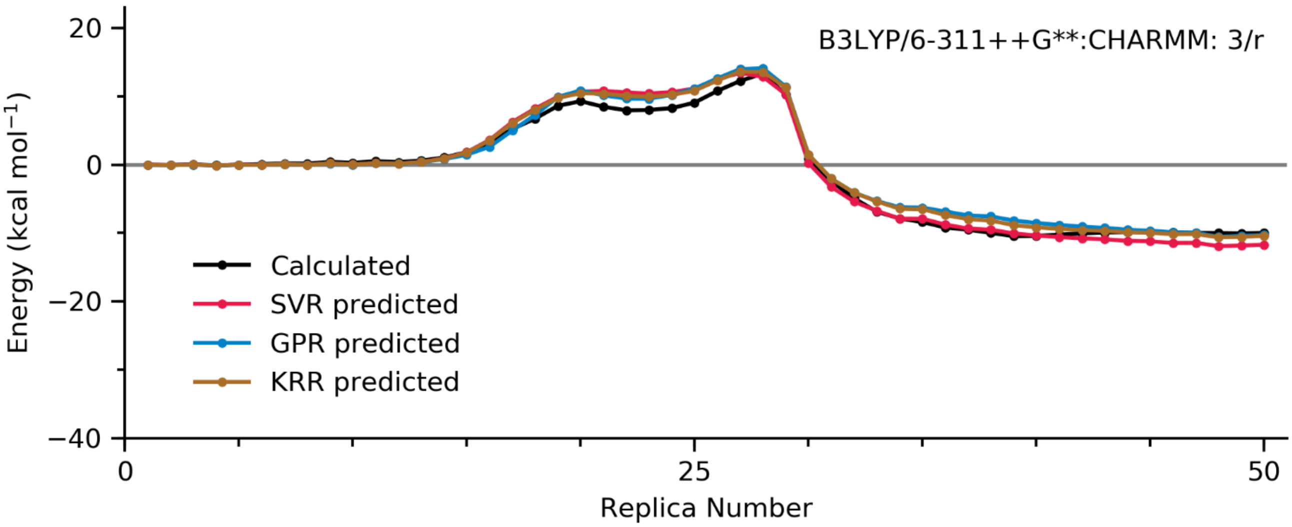
B3LYP/6-311++G**:CHARMM: 3/r
Energy (kcal mol^{-1})
20
0
−20
−40
0
25
50
Replica Number
Calculated
SVR predicted
GPR predicted
KRR predicted

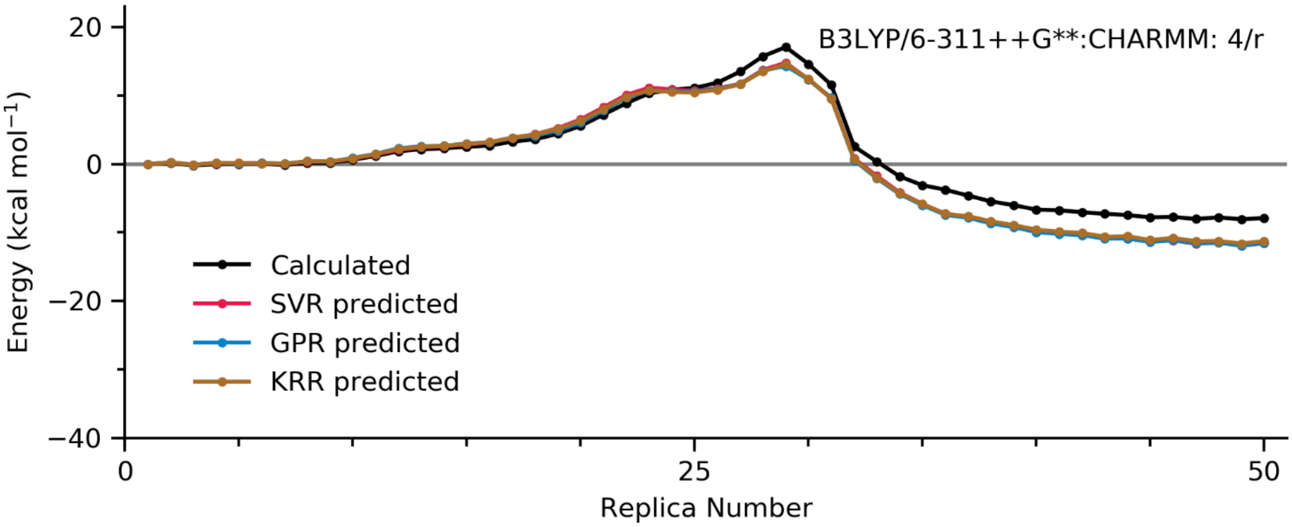
B3LYP/6-311++G**:CHARMM: 4/r
Energy (kcal mol^{-1})
20
0
−20
−40
0
25
50
Replica Number
Calculated
SVR predicted
GPR predicted
KRR predicted

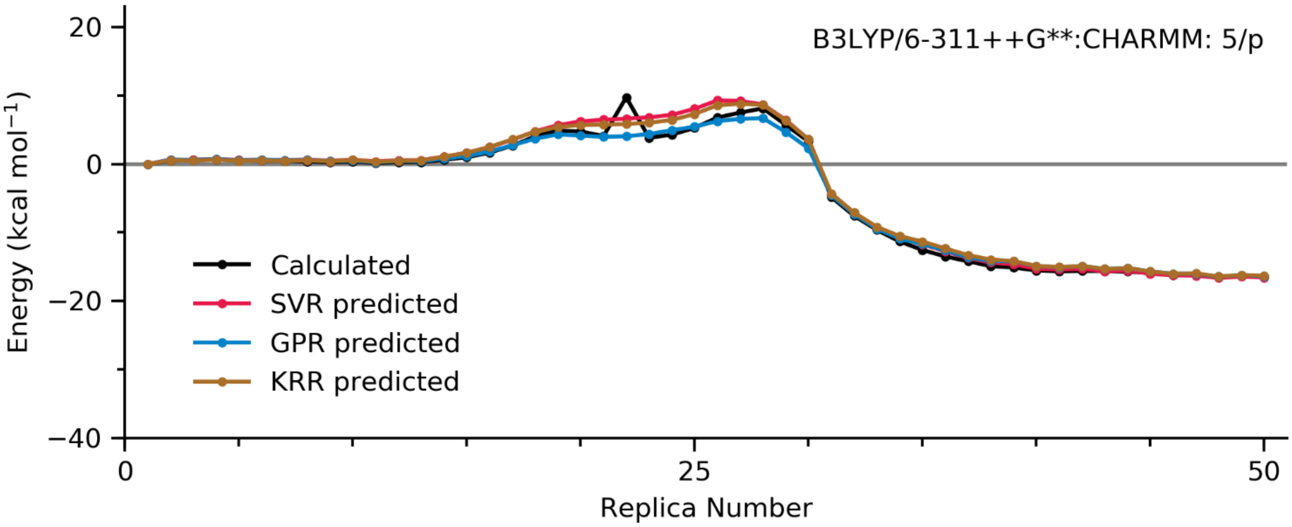
B3LYP/6-311++G**:CHARMM: 5/p
Energy (kcal mol^{-1})
20
0
−20
−40
0
25
50
Replica Number
Calculated
SVR predicted
GPR predicted
KRR predicted

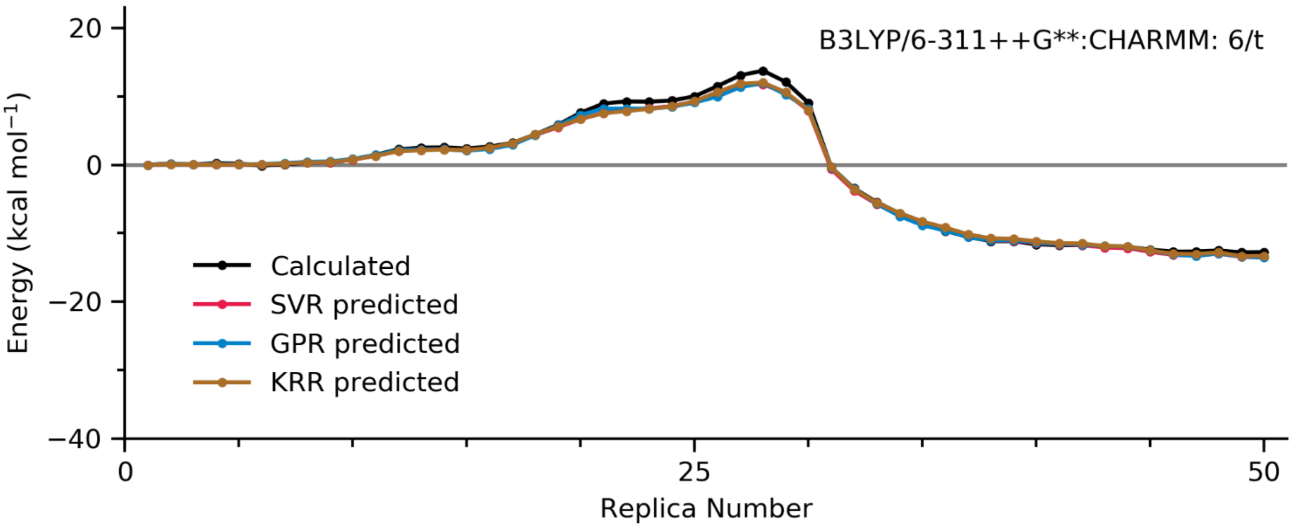
B3LYP/6-311++G**:CHARMM: 6/t
Energy (kcal mol^{-1})
Replica Number
Calculated
SVR predicted
GPR predicted
KRR predicted

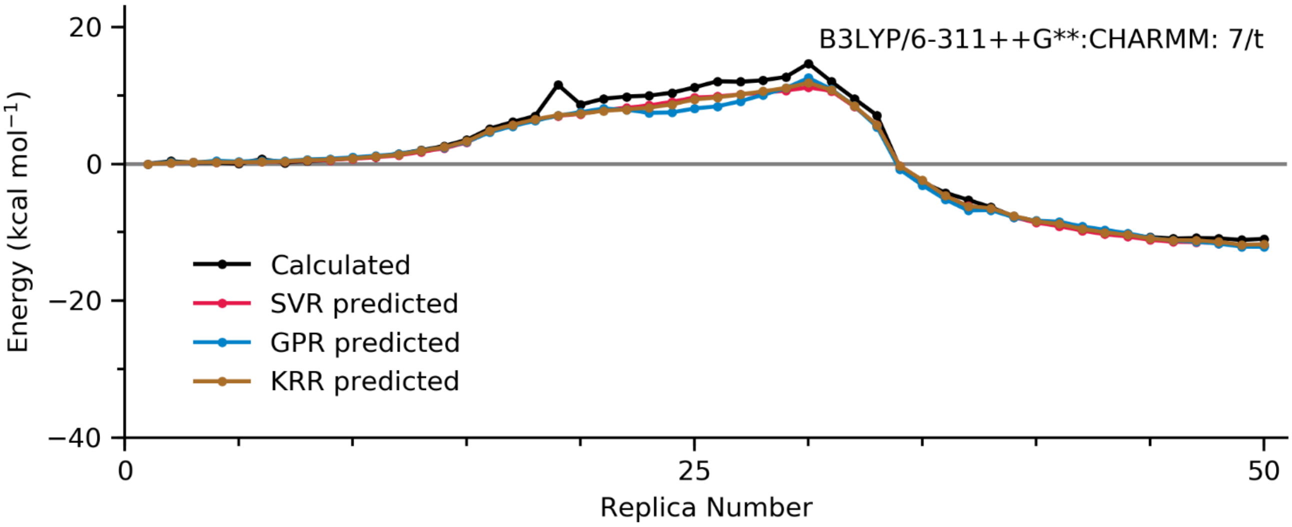
B3LYP/6-311++G**:CHARMM: 7/t
Energy (kcal mol^{-1})
Replica Number
Calculated
SVR predicted
GPR predicted
KRR predicted

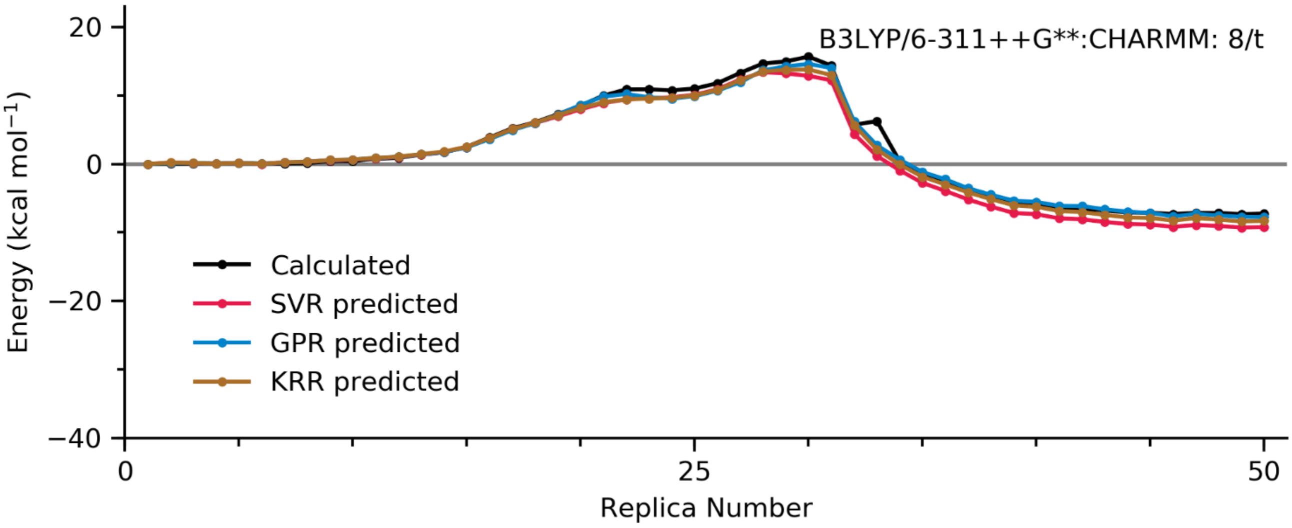
B3LYP/6-311++G**:CHARMM: 8/t
Energy (kcal mol^{-1})
Replica Number
Calculated
SVR predicted
GPR predicted
KRR predicted

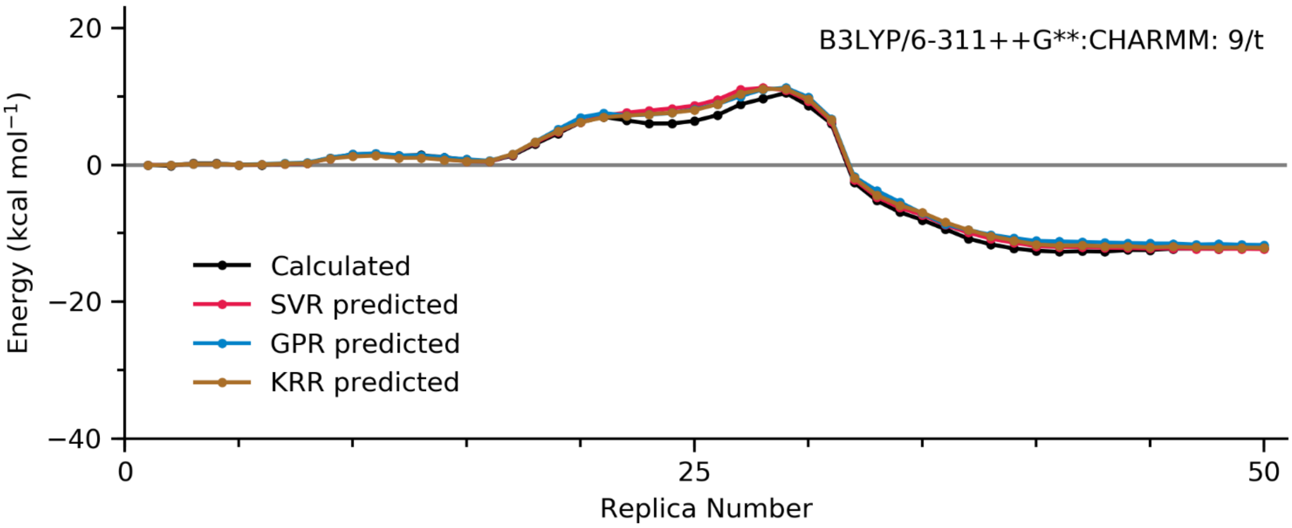

B3LYP/6-311++G**:CHARMM: 9/t
Energy (kcal mol^{-1})
Replica Number
Calculated
SVR predicted
GPR predicted
KRR predicted

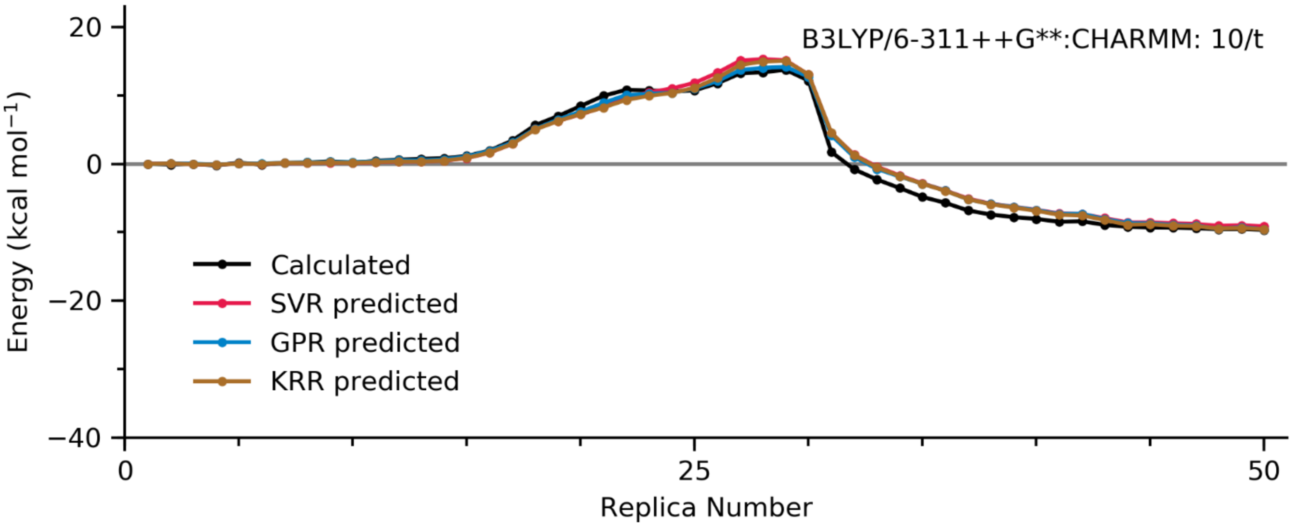

B3LYP/6-311++G**:CHARMM: 10/t
Energy (kcal mol^{-1})
Replica Number
Calculated
SVR predicted
GPR predicted
KRR predicted

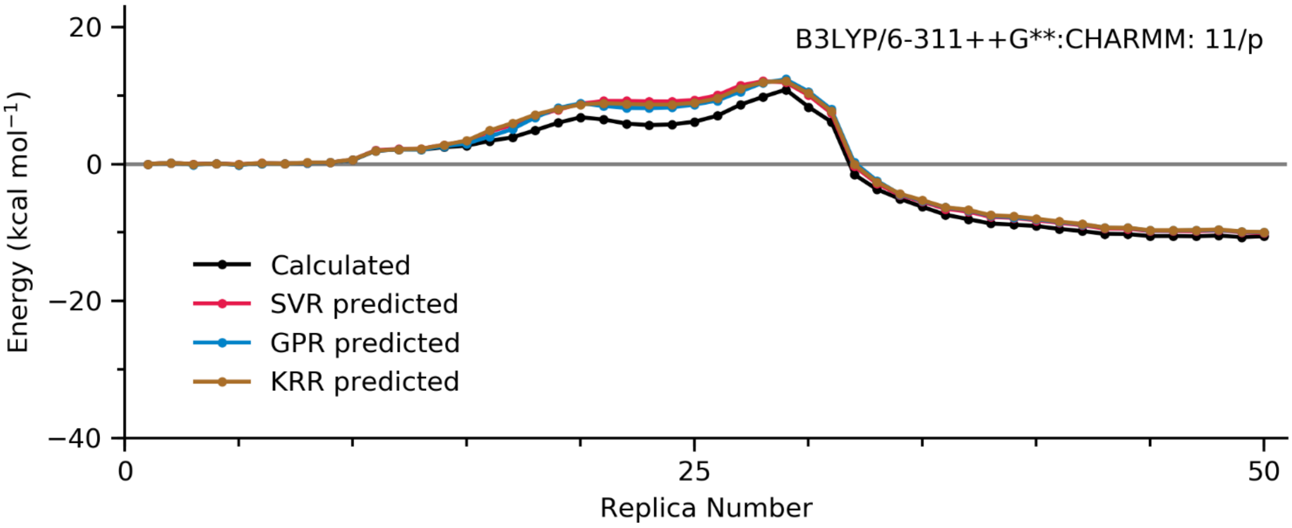

B3LYP/6-311++G**:CHARMM: 11/p
Energy (kcal mol^{-1})
Replica Number
Calculated
SVR predicted
GPR predicted
KRR predicted

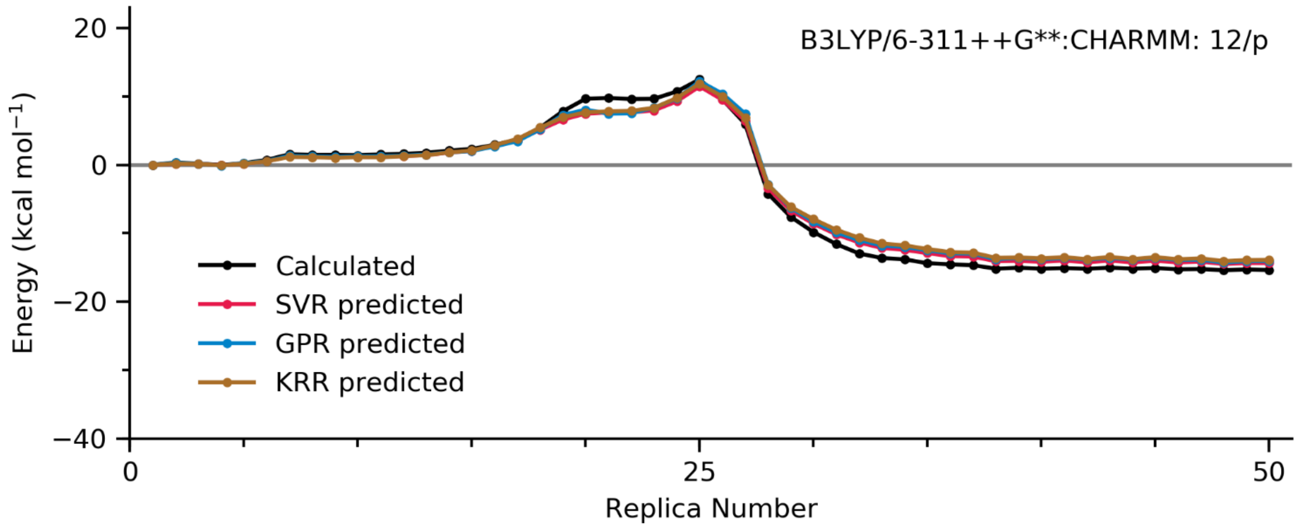
B3LYP/6-311++G**:CHARMM: 12/p
Calculated
SVR predicted
GPR predicted
KRR predicted
Energy (kcal mol^{-1})
Replica Number

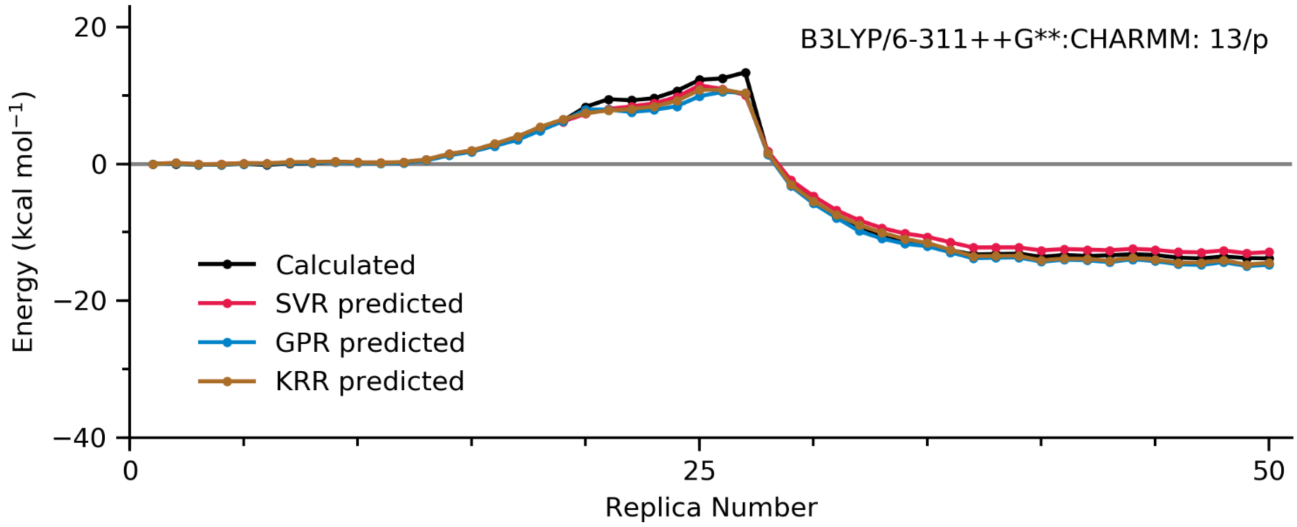
B3LYP/6-311++G**:CHARMM: 13/p
Calculated
SVR predicted
GPR predicted
KRR predicted
Energy (kcal mol^{-1})
Replica Number

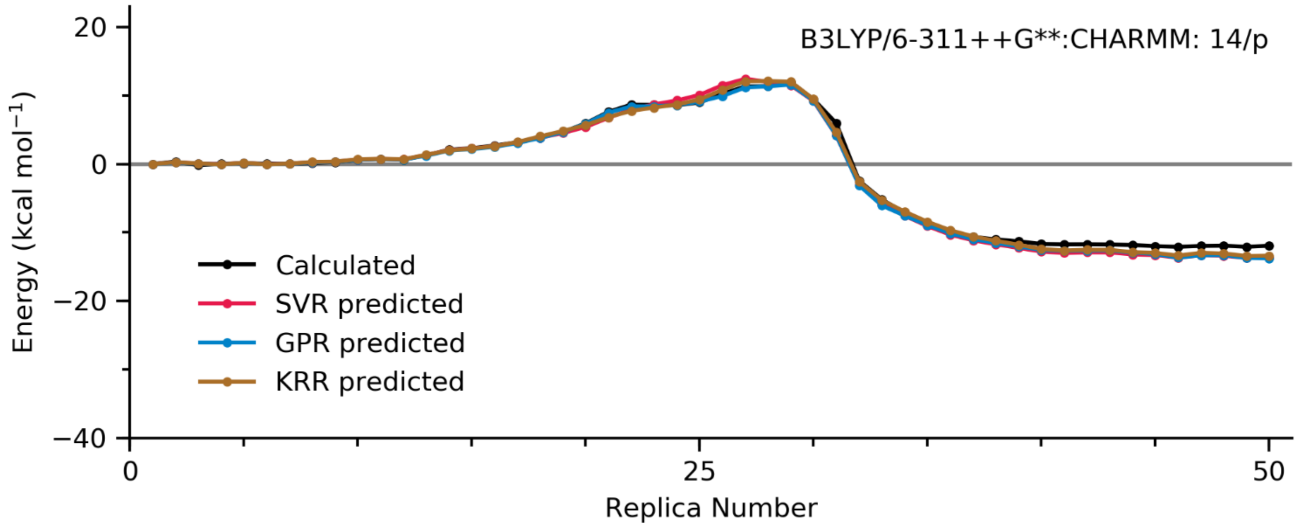
B3LYP/6-311++G**:CHARMM: 14/p
Calculated
SVR predicted
GPR predicted
KRR predicted
Energy (kcal mol^{-1})
Replica Number

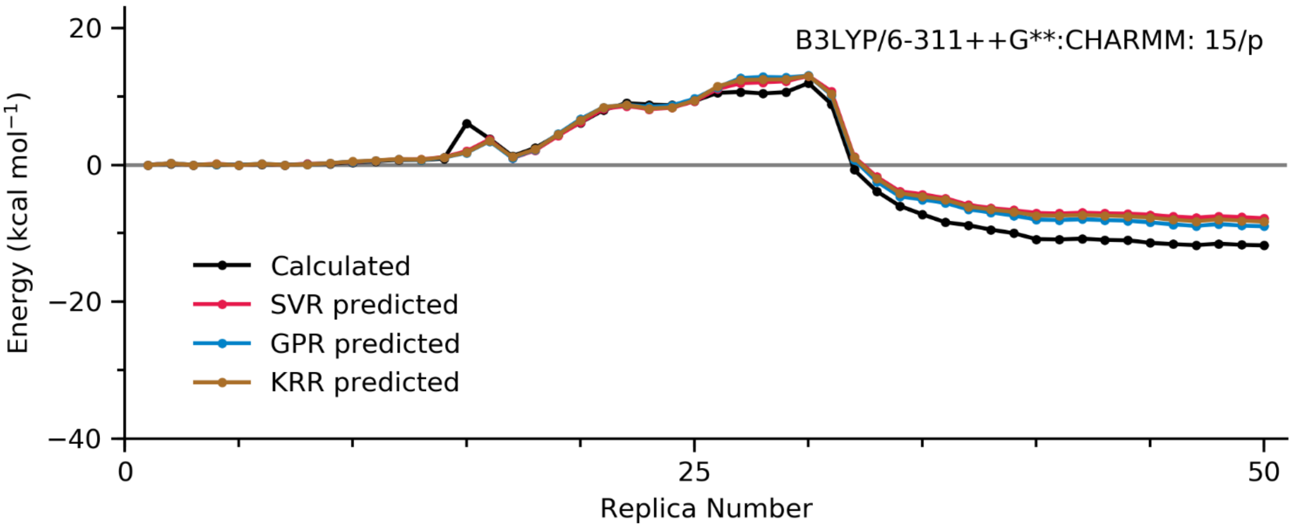
B3LYP/6-311++G**:CHARMM: 15/p
Energy (kcal mol^{-1})
Replica Number
Calculated
SVR predicted
GPR predicted
KRR predicted
20
0
−20
−40
0
25
50

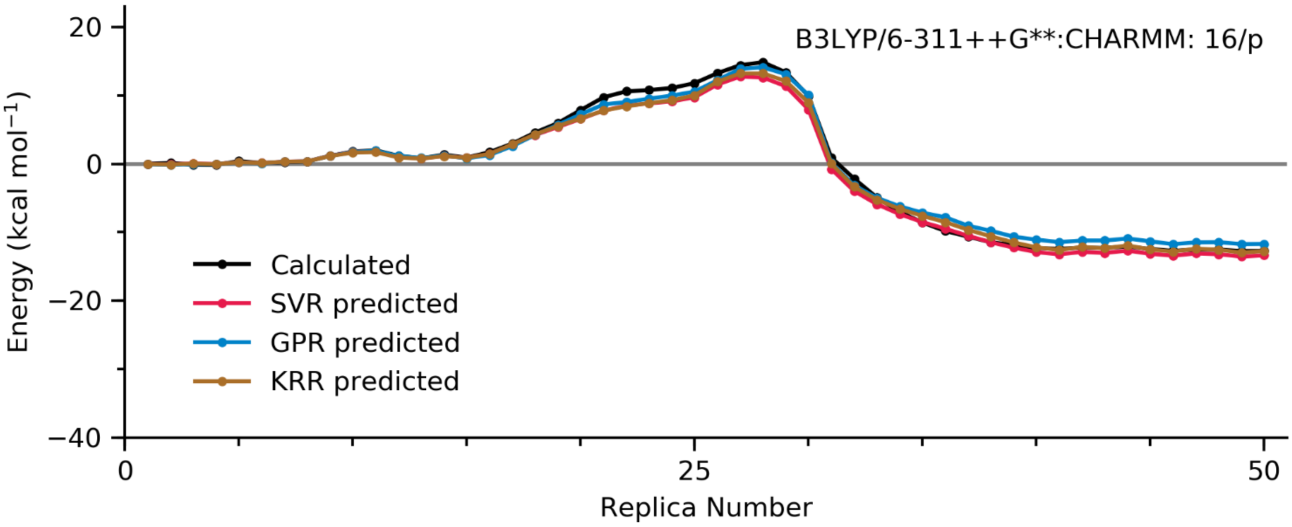
B3LYP/6-311++G**:CHARMM: 16/p
Energy (kcal mol^{-1})
Replica Number
Calculated
SVR predicted
GPR predicted
KRR predicted
20
0
−20
−40
0
25
50

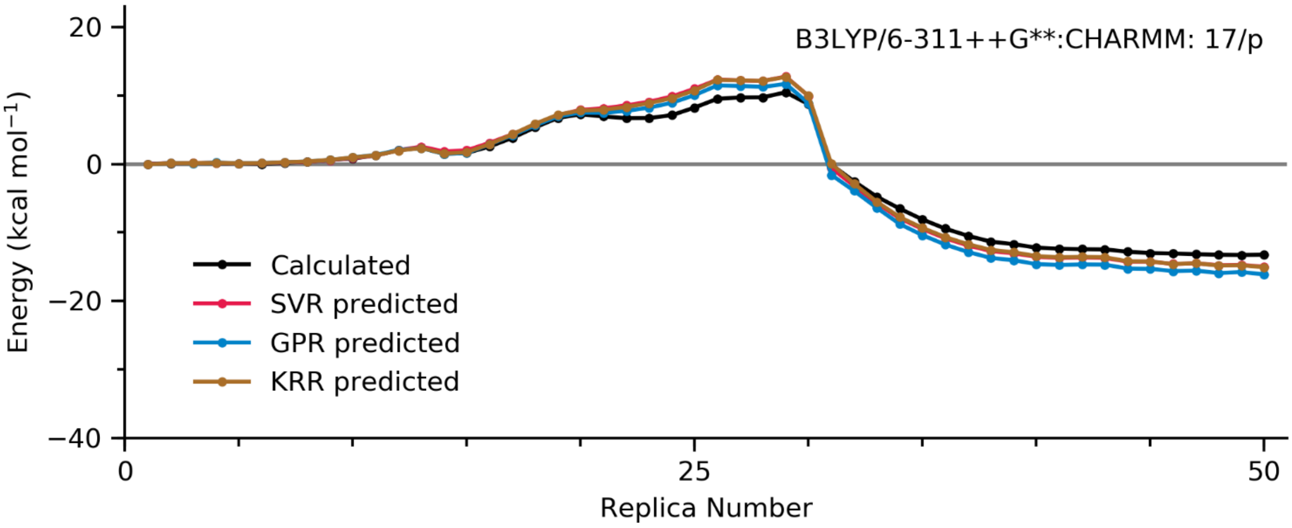
B3LYP/6-311++G**:CHARMM: 17/p
Energy (kcal mol^{-1})
Replica Number
Calculated
SVR predicted
GPR predicted
KRR predicted
20
0
−20
−40
0
25
50

Supplementary Figure 125 to 142. B3LYP-D3/6-311++G**:CHARMM pathways

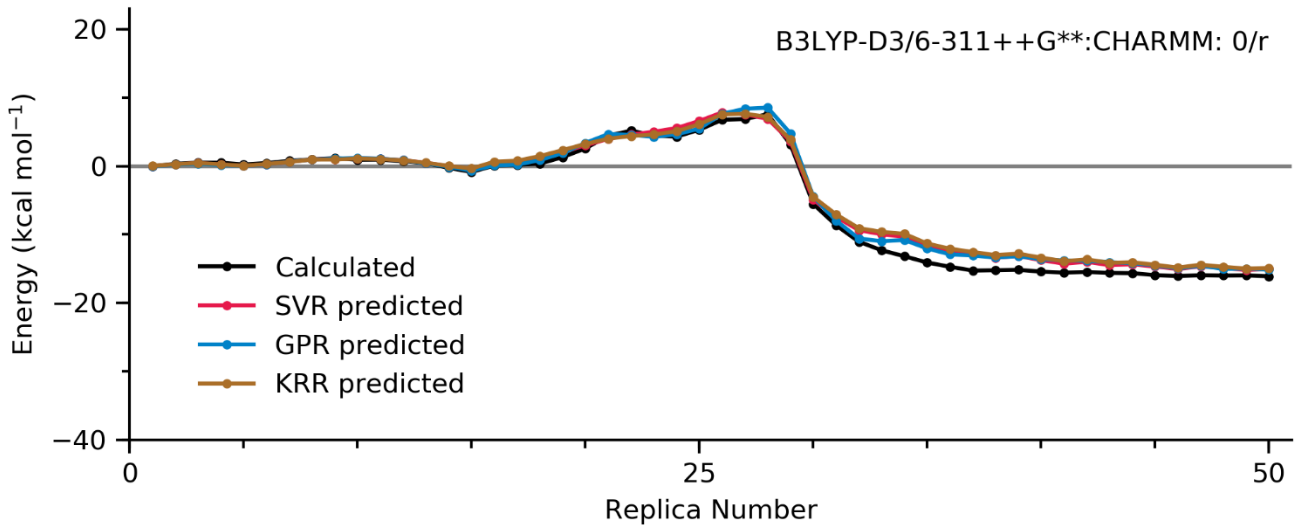

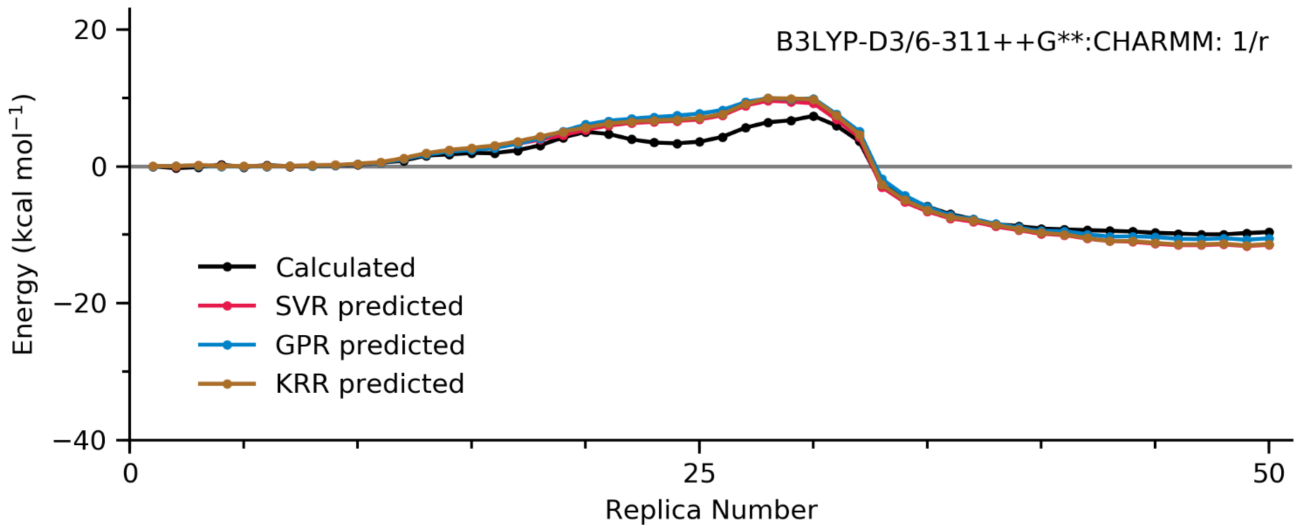

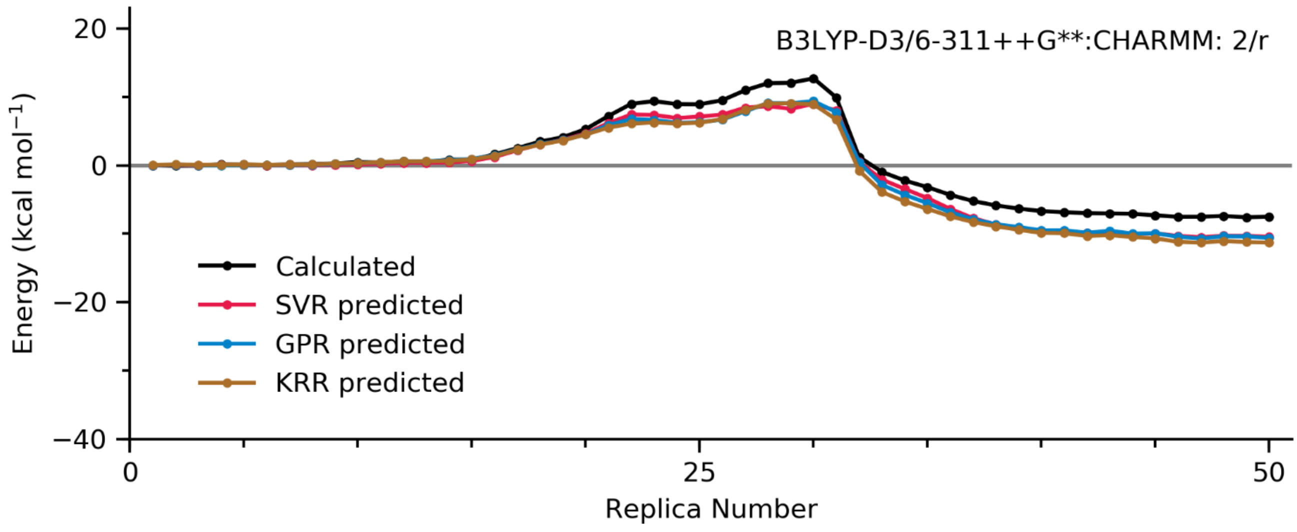

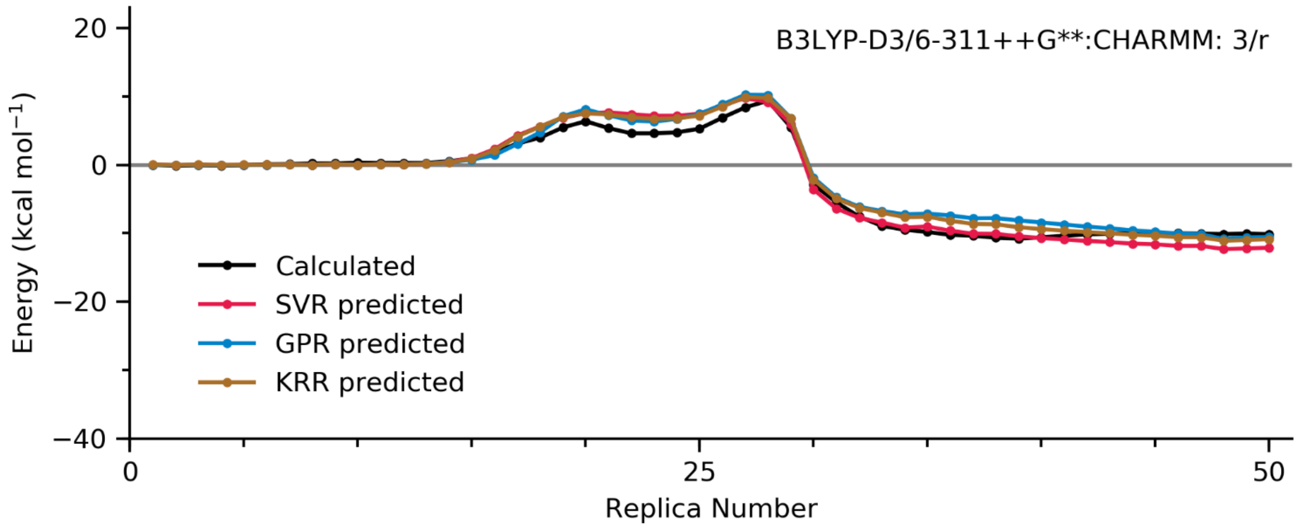
B3LYP-D3/6-311++G**:CHARMM: 3/r
Energy (kcal mol^{-1})
Replica Number
Calculated
SVR predicted
GPR predicted
KRR predicted

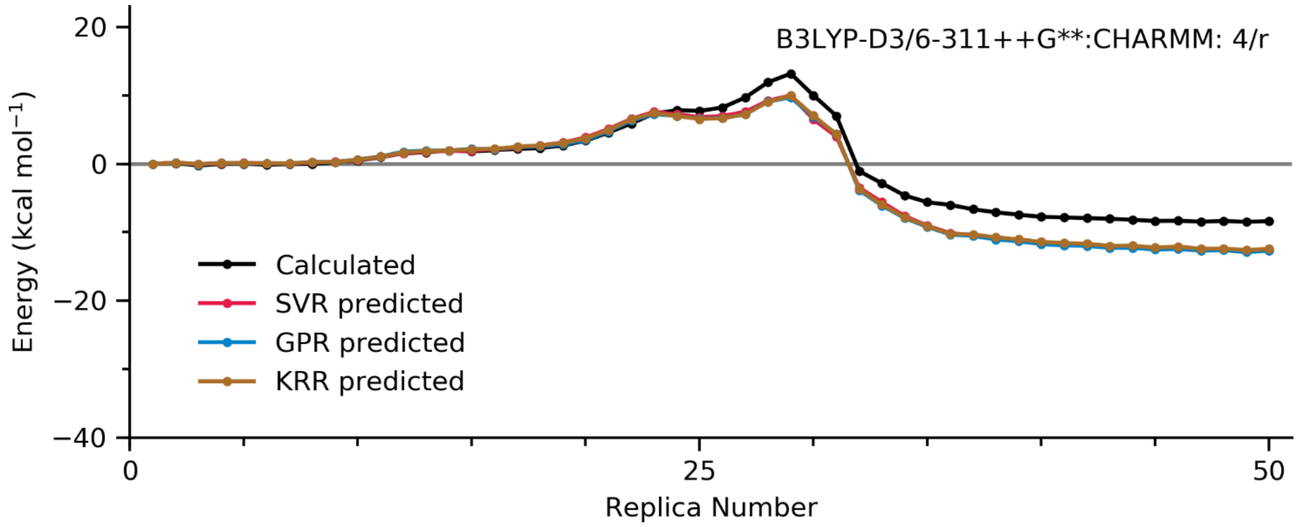
B3LYP-D3/6-311++G**:CHARMM: 4/r
Energy (kcal mol^{-1})
Replica Number
Calculated
SVR predicted
GPR predicted
KRR predicted

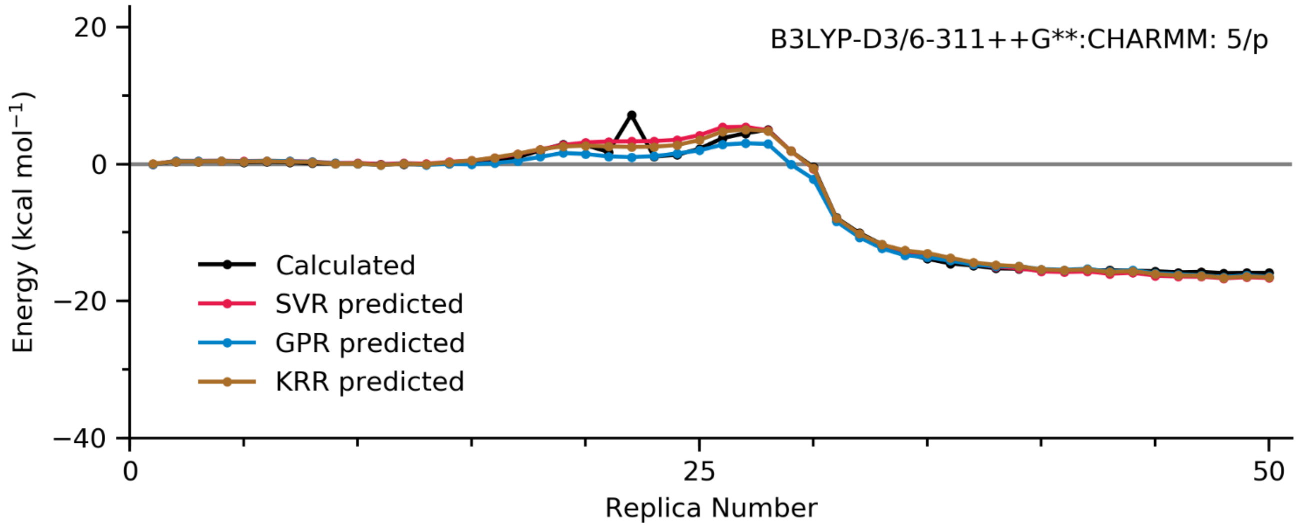
B3LYP-D3/6-311++G**:CHARMM: 5/p
Energy (kcal mol^{-1})
Replica Number
Calculated
SVR predicted
GPR predicted
KRR predicted

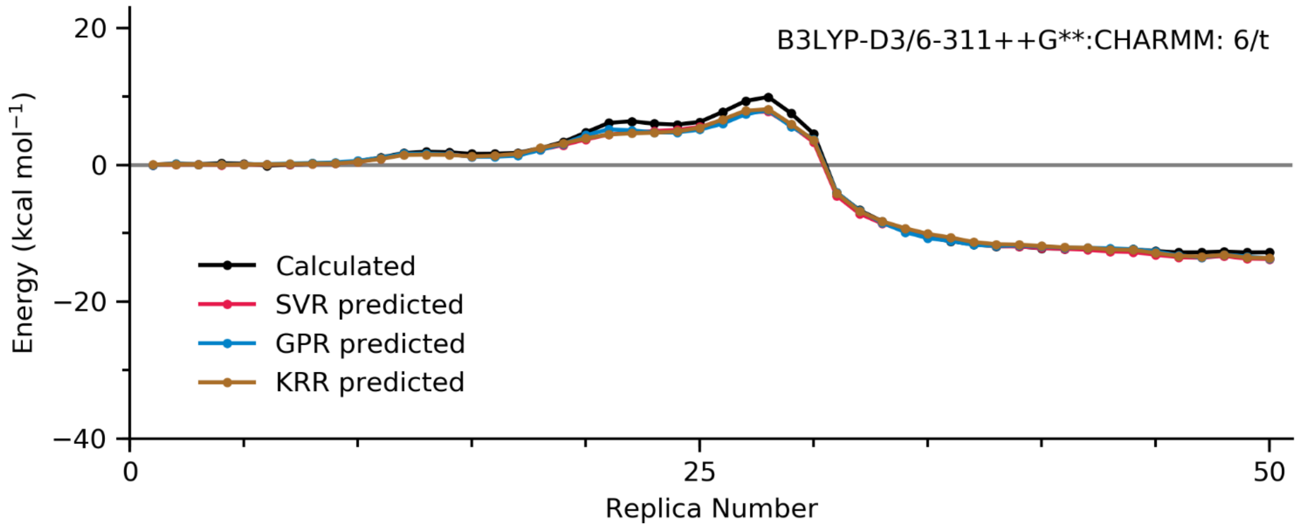

B3LYP-D3/6-311++G**:CHARMM: 6/t
Energy (kcal mol^{-1})
Replica Number
Calculated
SVR predicted
GPR predicted
KRR predicted

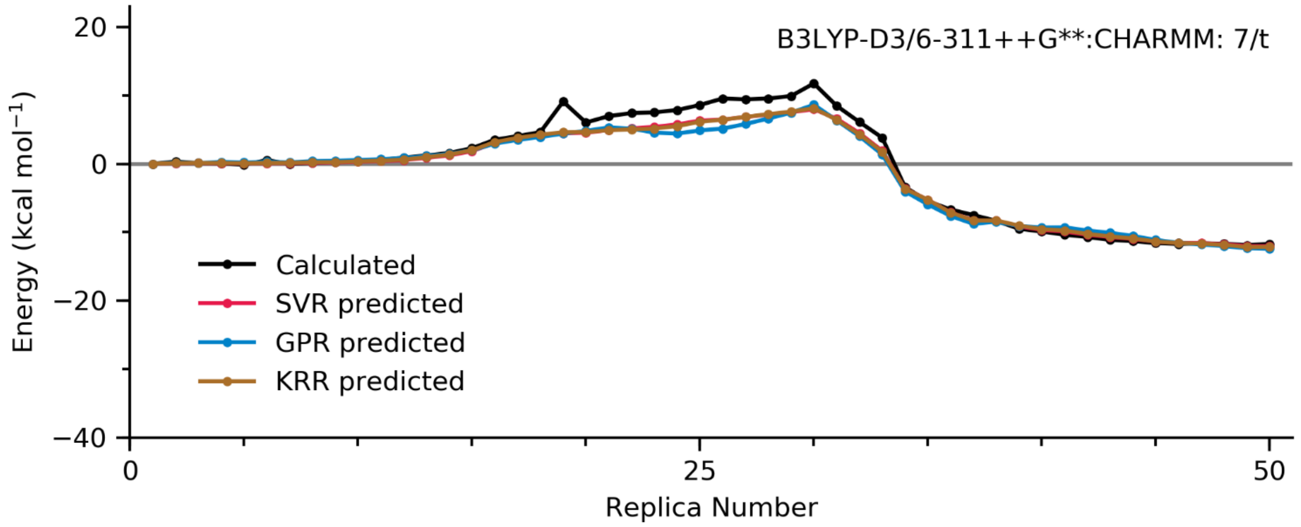

B3LYP-D3/6-311++G**:CHARMM: 7/t
Energy (kcal mol^{-1})
Replica Number
Calculated
SVR predicted
GPR predicted
KRR predicted

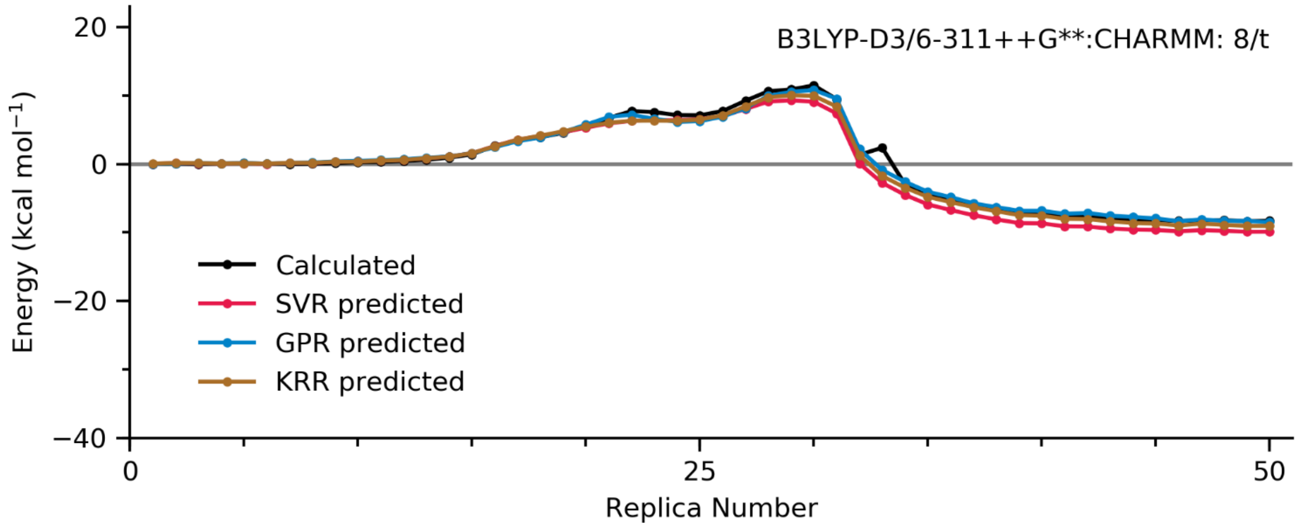

B3LYP-D3/6-311++G**:CHARMM: 8/t
Energy (kcal mol^{-1})
Replica Number
Calculated
SVR predicted
GPR predicted
KRR predicted

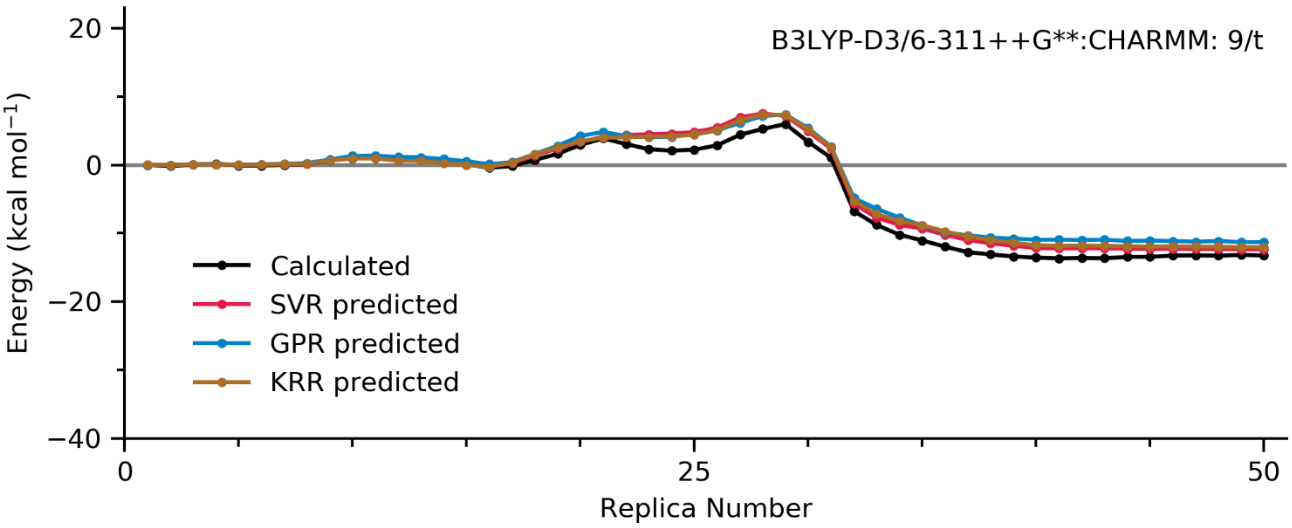
B3LYP-D3/6-311++G**:CHARMM: 9/t
Energy (kcal mol−1)
20
0
−20
−40
0
25
50
Replica Number
Calculated
SVR predicted
GPR predicted
KRR predicted

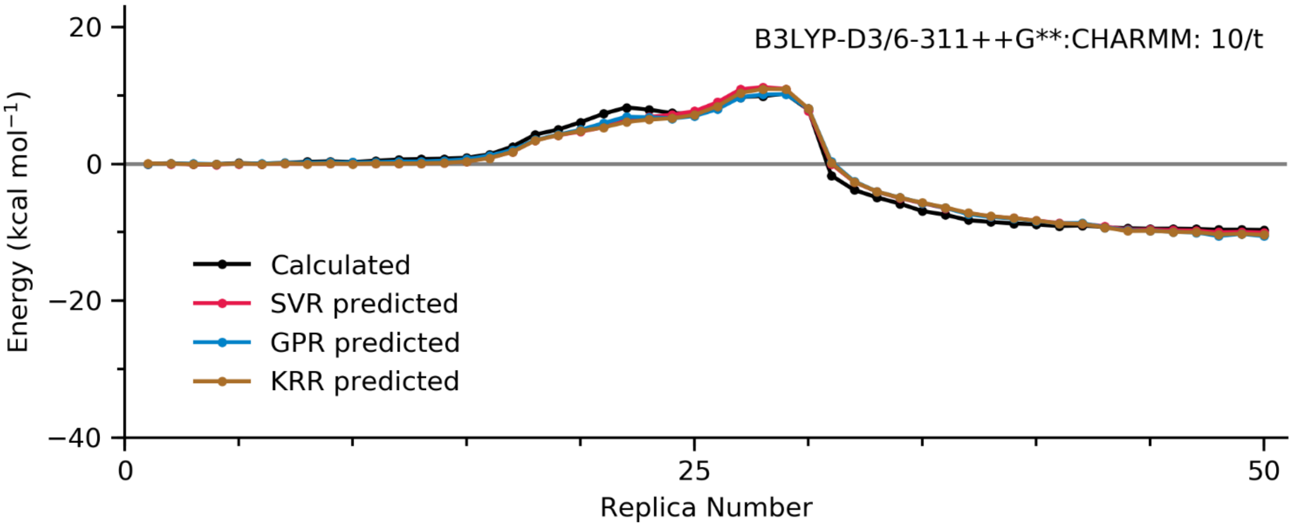
B3LYP-D3/6-311++G**:CHARMM: 10/t
Energy (kcal mol−1)
20
0
−20
−40
0
25
50
Replica Number
Calculated
SVR predicted
GPR predicted
KRR predicted

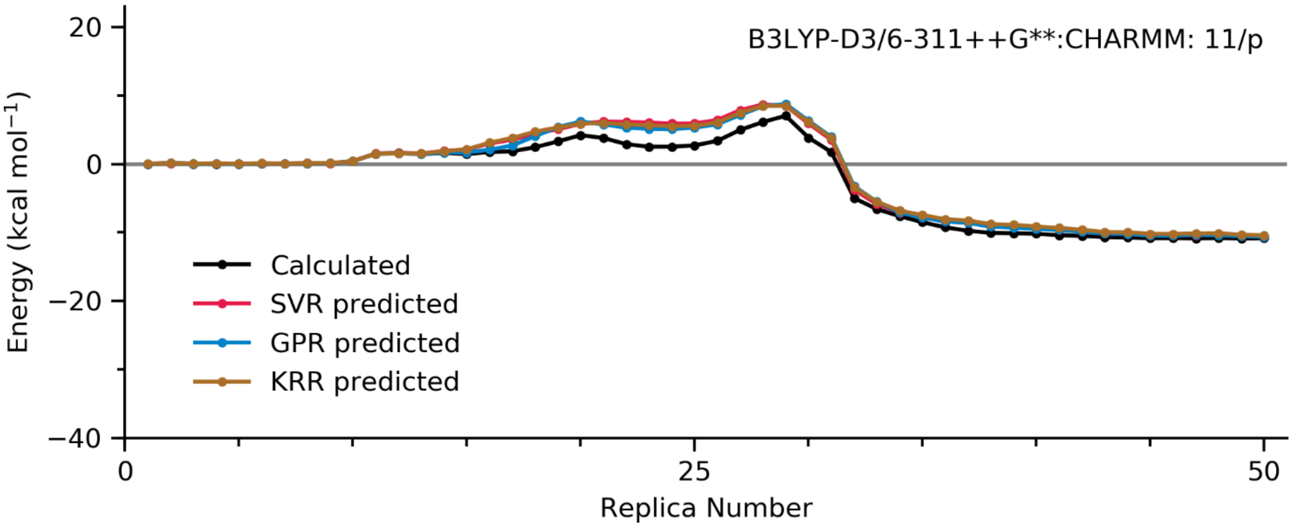
B3LYP-D3/6-311++G**:CHARMM: 11/p
Energy (kcal mol−1)
20
0
−20
−40
0
25
50
Replica Number
Calculated
SVR predicted
GPR predicted
KRR predicted

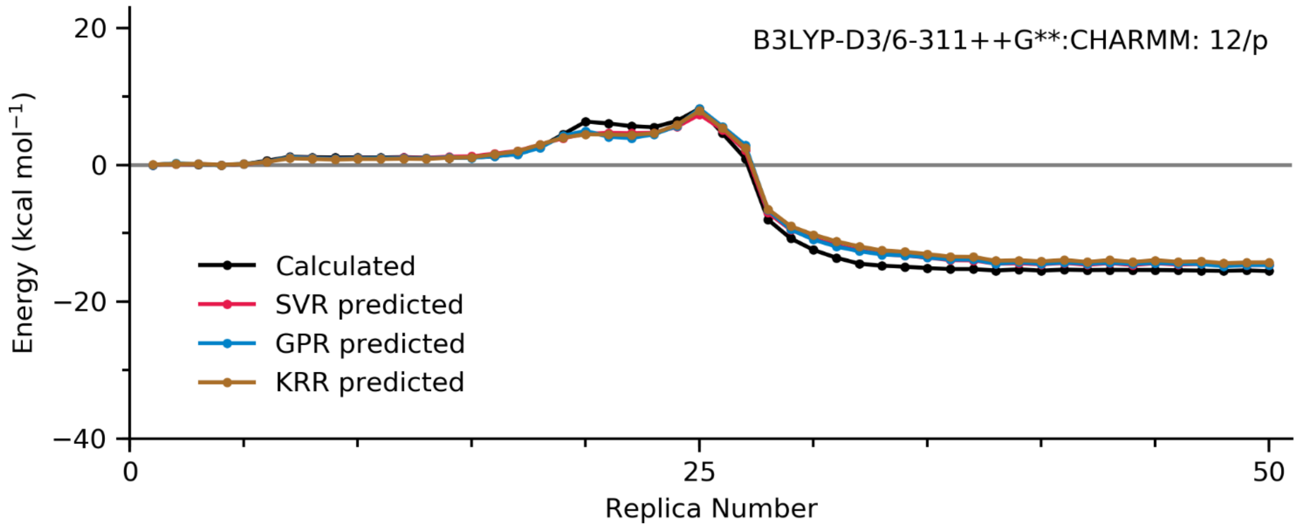

B3LYP-D3/6-311++G**:CHARMM: 12/p
Energy (kcal mol^{-1})
Replica Number
Calculated
SVR predicted
GPR predicted
KRR predicted

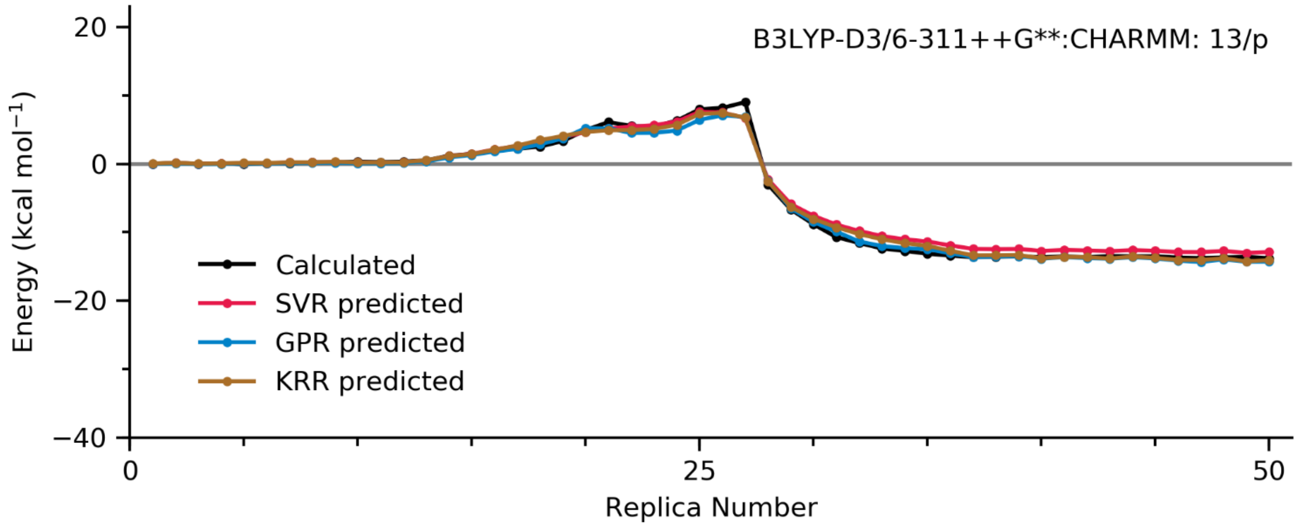

B3LYP-D3/6-311++G**:CHARMM: 13/p
Energy (kcal mol^{-1})
Replica Number
Calculated
SVR predicted
GPR predicted
KRR predicted

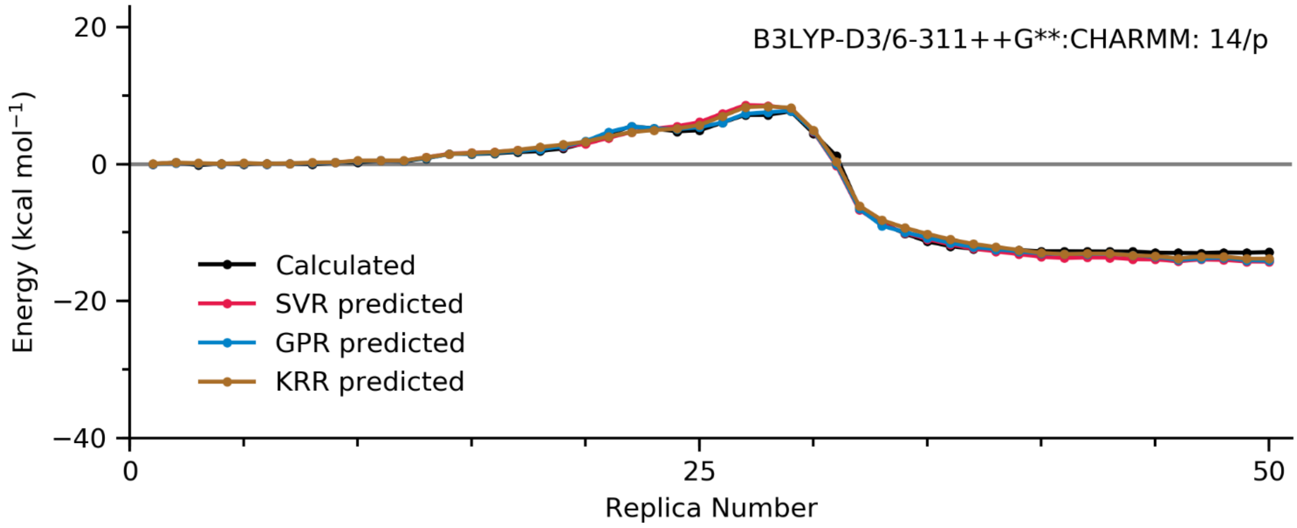

B3LYP-D3/6-311++G**:CHARMM: 14/p
Energy (kcal mol^{-1})
Replica Number
Calculated
SVR predicted
GPR predicted
KRR predicted

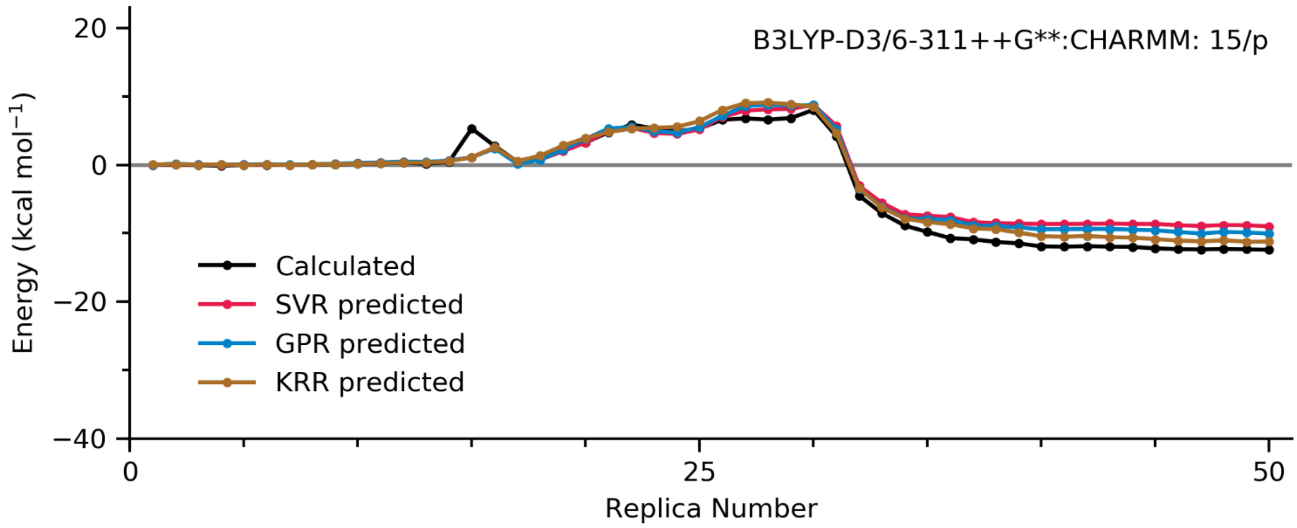

B3LYP-D3/6-311++G**:CHARMM: 15/p
Energy (kcal mol^{-1})
Replica Number
Calculated
SVR predicted
GPR predicted
KRR predicted

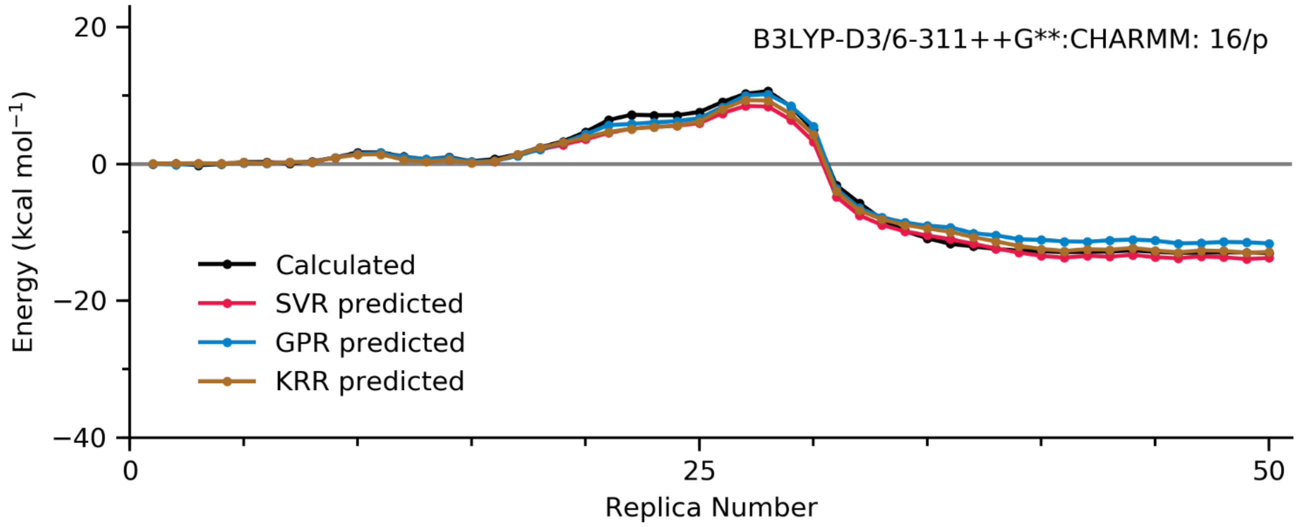

B3LYP-D3/6-311++G**:CHARMM: 16/p
Energy (kcal mol^{-1})
Replica Number
Calculated
SVR predicted
GPR predicted
KRR predicted

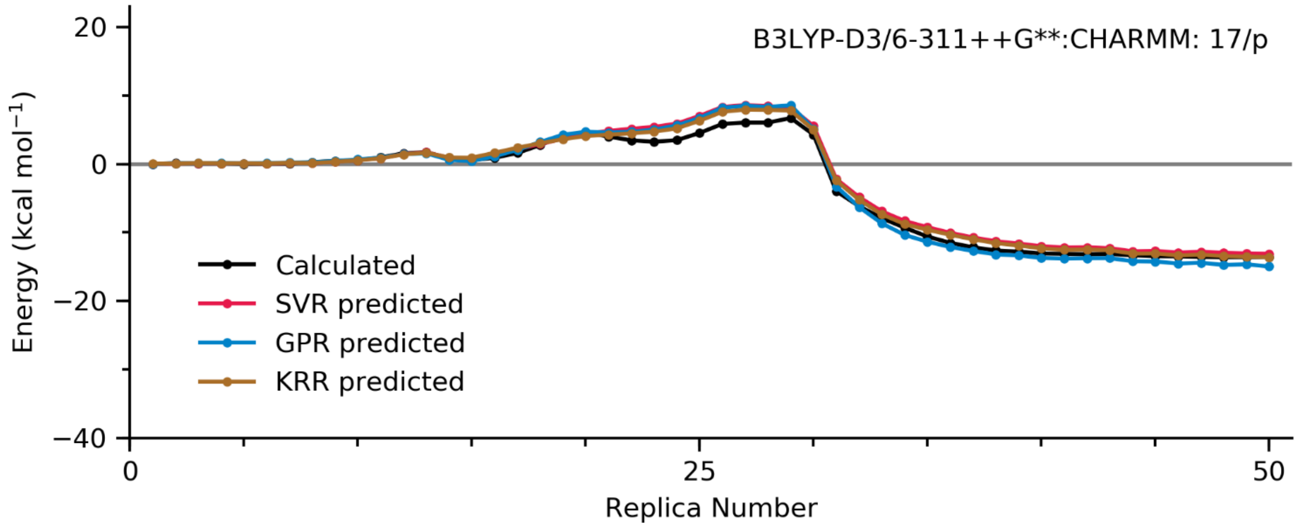

B3LYP-D3/6-311++G**:CHARMM: 17/p
Energy (kcal mol^{-1})
Replica Number
Calculated
SVR predicted
GPR predicted
KRR predicted

Supplementary Figure 143

Supplementary Figure 143. The Prediction quality of models trained with training sets that omitted certain feature groups (the $f_{\mathrm{a}=0}$ models, see main text equation 2).

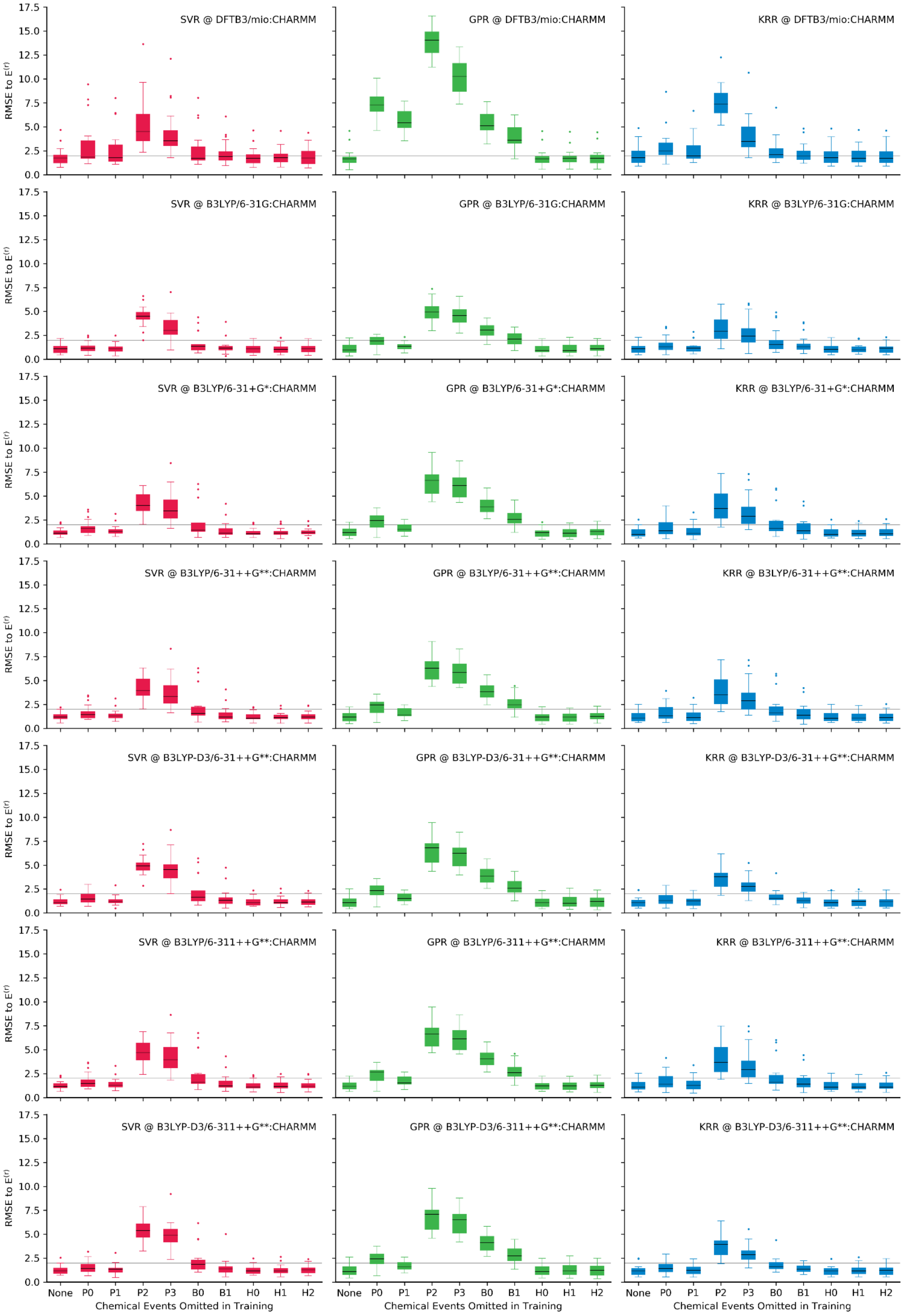
SVR @ DFTB3/mio:CHARMM
GPR @ DFTB3/mio:CHARMM
KRR @ DFTB3/mio:CHARMM
SVR @ B3LYP/6-31G:CHARMM
GPR @ B3LYP/6-31G:CHARMM
KRR @ B3LYP/6-31G:CHARMM
SVR @ B3LYP/6-31+G*:CHARMM
GPR @ B3LYP/6-31+G*:CHARMM
KRR @ B3LYP/6-31+G*:CHARMM
SVR @ B3LYP/6-31++G**:CHARMM
GPR @ B3LYP/6-31++G**:CHARMM
KRR @ B3LYP/6-31++G**:CHARMM
SVR @ B3LYP-D3/6-31++G**:CHARMM
GPR @ B3LYP-D3/6-31++G**:CHARMM
KRR @ B3LYP-D3/6-31++G**:CHARMM
SVR @ B3LYP/6-311++G**:CHARMM
GPR @ B3LYP/6-311++G**:CHARMM
KRR @ B3LYP/6-311++G**:CHARMM
SVR @ B3LYP-D3/6-311++G**:CHARMM
GPR @ B3LYP-D3/6-311++G**:CHARMM
KRR @ B3LYP-D3/6-311++G**:CHARMM
RMSE to E(r)
0.0 2.5 5.0 7.5 10.0 12.5 15.0 17.5
None P0 P1 P2 P3 B0 B1 H0 H1 H2
Chemical Events Omitted in Training

The 'None' label on the X-axis denotes the regression model was trained with full feature set (the f models) and the box in here corresponds to the RMSEs in Figure 3d. We herein demonstrate further information on the intrinsic energy contribution measurement. The intrinsic energy contribution is defined as the decrease of prediction quality to the "predicted" energy profile, as shown in Figure 5, Supplementary Figure 9 and 10. We herein provide Supplementary Figure 143 to demonstrate the decrease of prediction quality to the "calculated" energy profile. We note that Supplementary Figure 9 and 143 fundamentally illustrate different quantities. Each box contains n = 18 testing cases, the IQR noted by the boxes are divided by the median (black lines), and the whiskers mark the first datum that are larger than 1.5 * IQR. See also Supplementary Table 1.

Supplementary Note 1

Discussions regarding previous studies on the acylation profile

The discussion on the benzylpenicillin acylation profiles is essentially a revisiting to the acylation energy profiles and the thermal stability of the meta-stable states (the tetrahedral intermediate).

In the main text, the deviation between the data reported by Hermann *et al.* (ref. 17) and Meroueh *et al.* (ref. 18) is concluded as the consequence of excluding P2 and P3 during the scan of the tetrahedral formation PES. In their studies, P2 and P3 as critical RCs would very likely be discontinuous in the transition from the reactant to the tetrahedral intermediates. In this case, the barrier height or the thermal stability of meta-stable states would depend on the initial configuration where the PES scans started, and/or how the PES scans were conducted.

Most importantly, as stated in the main text, the general mechanistic insights from the above-mentioned studies remain solid and complete, as the most critical RCs were used in the PES scan at each stage of the acylation.

Additionally, we also note that in Ref 18, Meroueh *et al.* did comment on the actual rate limiting event during the acylation, as we quote:

"Intuitively, the largest barrier in this mechanism is not tetrahedral formation but the delivery of the proton to the very weakly basic amide of the β-lactam."

Albeit their intuitive insights are not accompanied by any data, we note that their comment based on their intuition is proven to be correct in the current study, as we showed that P2 and P3 are the rate limiting events during the acylation (Figure 5, Supplementary Figure 10).
