# Supplementary material for: Unraveling the energetic significance of chemical events in enzyme catalysis via machine-learning based regression approach
Source: Commun Chem. 2020 Oct 8;3:134. doi: 10.1038/s42004-020-00379-w (PMC9814854; doi:10.1038/s42004-020-00379-w)
Supplement: Supplementary file 2 — Description of Additional Supplementary Files [file 42004_2020_379_MOESM2_ESM.pdf]

Description of Additional Supplementary Files

File Name: Supplementary Data 1
Description: The pathways coordinates are named by “ID/state”, e.g., “path_0_r.cor” is the coordinate file for pathway 0 which is sampled from the reactant QM state. All the pathway coordinate files share the same protein structure file, path.psf.
